# Shifts in plant foliar and floral metabolomes in response to the suppression of the associated microbiota

**DOI:** 10.1186/s12870-016-0767-7

**Published:** 2016-04-06

**Authors:** Albert Gargallo-Garriga, Jordi Sardans, Míriam Pérez-Trujillo, Alex Guenther, Joan Llusià, Laura Rico, Jaume Terradas, Gerard Farré-Armengol, Iolanda Filella, Teodor Parella, Josep Peñuelas

**Affiliations:** CSIC, Global Ecology Unit CREAF- CSIC-UAB, Cerdanyola del Vallès, Catalonia 08193 Spain; CREAF, Cerdanyola del Vallès, Catalonia 08193 Spain; Service of Nuclear Magnetic Resonance, Universitat Autònoma de Barcelona, Cerdanyola del Vallès, Catalonia 08913 Spain; Pacific Northwest National Laboratory, Richland, WA 99354 USA; Department BABVE, Universitat Autònoma de Barcelona, Barcelona, Catalonia 08913 Spain

**Keywords:** Epiphytic and endophytic microbiota, metabolites, antibiotics, *Sambucus nigra*

## Abstract

**Background:**

The phyllospheric microbiota is assumed to play a key role in the metabolism of host plants. Its role in determining the epiphytic and internal plant metabolome, however, remains to be investigated. We analyzed the Liquid Chromatography-Mass Spectrometry (LC-MS) profiles of the epiphytic and internal metabolomes of the leaves and flowers of *Sambucus nigra* with and without external antibiotic treatment application.

**Results:**

The epiphytic metabolism showed a degree of complexity similar to that of the plant organs. The suppression of microbial communities by topical applications of antibiotics had a greater impact on the epiphytic metabolome than on the internal metabolomes of the plant organs, although even the latter changed significantly both in leaves and flowers.

The application of antibiotics decreased the concentration of lactate in both epiphytic and organ metabolomes, and the concentrations of citraconic acid, acetyl-CoA, isoleucine, and several secondary compounds such as terpenes and phenols in the epiphytic extracts. The metabolite pyrogallol appeared in the floral epiphytic community only after the treatment. The concentrations of the amino acid precursors of the ketoglutarate-synthesis pathway tended to decrease in the leaves and to increase in the foliar epiphytic extracts.

**Conclusions:**

These results suggest that anaerobic and/or facultative anaerobic bacteria were present in high numbers in the phyllosphere and in the apoplasts of *S. nigra.* The results also show that microbial communities play a significant role in the metabolomes of plant organs and could have more complex and frequent mutualistic, saprophytic, and/or parasitic relationships with internal plant metabolism than currently assumed.

**Electronic supplementary material:**

The online version of this article (doi:10.1186/s12870-016-0767-7) contains supplementary material, which is available to authorized users.

## Background

Distinct microbial communities hosted in and on plant organs are especially important in roots [1, 2] but also in leaves [3, 4]. Epiphytic organisms, such as bacteria and fungi, colonize the surfaces of aerial plant organs. Microbes can arrive to or depart from surfaces of leaves through the action of rain, wind, or insects [5].

For phyllospheric microorganisms, the potential benefits of living on leaves are obvious and include supplies of nutrients [6, 7] and carbon [7, 8]. The bacteria themselves could also influence substrate availability by producing substances that increase substrate leaching from plant organs to the surface [9]. The advantages provided by phyllospheric inhabitants to their host plants, however, are not as apparent.

Some reports have shown that both internal and external foliar microbiotas exert several effects on plants, including indirect protection against pathogens [10–12], protecting plants from diseases and promoting plant growth by various mechanisms [6, 13], and plant communication by affecting emissions of volatile organic compounds [11, 12, 14]. The relationships between microorganisms and their hosts include parasitic, commensal, and mutualistic interactions [15]. The classification of these relationships can be difficult, principally the discrimination between commensals and mutualistic symbionts, which represent a continuum [16]. Many members of the human gut bacterial community were previously considered commensals but are now regarded as beneficial symbionts because of their contributions to host metabolism and immunity [17]. Similar questions of host benefit and microorganism-microorganism interactions should be asked about the microbial communities associated with plants [18, 19].

Foliar surfaces are habitually poor in nutrient availability, but significant amounts of organic carbon have been detected, including carbohydrates, amino acids, organic acids, and sugar alcohols [20–22]. The heterogeneous nature of nutrient availability has been clearly observed on foliar surfaces [23, 24]. The correlations of foliar mass per area, and nitrogen and phosphorus concentrations with foliar bacterial community structure have been well documented [25, 26]. In addition to the carbon sources, volatile plant-derived metabolic substrates, including isoprenes and C1 compounds [27], have been identified on foliar surfaces. Methanol, that is a primarily by-product of cell-wall metabolism by pectin methyl esterases, is a prominent C1 source for phyllospheric microorganisms and is released in diurnal cycles [27]. Methanol can serve as a substrate for a methylotrophic epiphytic bacterium (*Methylobacterium extorquens*) that confers a growth advantage to these organisms *in situ* [28, 29]. Bacterial communities on well-fertilized plants may be limited primarily by carbon availability and only secondarily by nitrogen availability [30]. Bacteria can use several nitrogen sources, including organic nitrogenous compounds such as amino acids, which could be valuable sources of nitrogen for phyllospheric bacteria. Ammonia may also be used as a nitrogen source in the phyllosphere [31], and nitrogen fixation by phyllospheric bacteria has been reported [4, 32]. Phyllospheric bacteria also need to take up other macro- and microelements for growth.

Plants produce a wide range of secondary metabolites with antimicrobial activity [33], and microorganisms can also produce antimicrobial metabolites [34]. Competition for space and nutrient resources, the production of antibiotics, and interference with cell-signaling systems in microbial communities are the principal mechanisms by which epiphytic bacteria and fungi antagonize each other [30, 35, 36].

The complete set of metabolites of the epiphytic habitat, however, has not yet been analyzed. Ecometabolomics [37–39] could provide such information. A metabolome is the entirety set of the small molecules in an organism as the final expression of its genotype [40] and can be considered as the organism’s chemical phenotype [37, 38]. Metabolomic techniques could be combined with the application of antibiotics against bacteria and fungi to discern the role in plant metabolism of microbial communities living on and in plant organs. In this way we aimed to determine the effect of microorganisms living into and on to plants on overall plant metabolism.

We have analyzed the metabolomes of the epiphytic habitats of leaves and flowers and of the organs themselves of the species *Sambucus Nigra* L. submitted to the application of antibiotics against bacteria and fungi. Our detailed objectives were: (i) to determine the changes in the metabolic profile of the plant surface when epiphytic microorganisms are suppressed, (ii) to determine the changes in the metabolic profile inside the plant organs when epiphytic and likely also endophytic microorganisms are suppressed, and (iii) to study the similarities and differences between the internal and epiphytic metabolomes. These aims also allowed us (iv) to investigate the synergies and antagonisms between the metabolic functions of the plants and the epiphytic microorganisms.

## Results

### Univariate analyses

#### Antibiotic assesment

Chloramphenicol and streptomycin were present in all organ and epiphytic samples of the antibiotic-treated plants from day 1. The concentration of the streptomycin decreased with time and was no longer detected at day 15 in the organs and epiphytic extracts. Chloramphenicol, however, was detected throughout the monitored period (30 days), though at day 30 it was detected only in leaves. Oxytetracycline was found only in the epiphytic extracts and only until day 15 (Additional file 1: Table S1).

### Organ versus epiphytic extracts

The concentrations of 80 % of the detected metabolites differed significantly between the leaves and their epiphytic extracts (1020 of the 1277) and between the flowers and their epiphytic extracts (1014 of the 1271). More metabolites were detected in the plant organs than in the epiphytic biofilms. A total of 1626 metabolic variables were detected, 1546 in the plant organs and 1220 in the epiphytic extracts (Additional file 1: Tables S2 and S3). A total of 1140 metabolites were detected in both the organs and the epiphytic extracts; 80 were detected in the epiphytic extracts but not in the organs, and 406 were detected in the organs but not in the epiphytic extracts. A total of 1277 metabolites were detected in leaves and in foliar epiphytic extracts, 196 (including aspartic acid, fisetin, nicotine, rhamnetin, and vitexin) were detected in leaves but not in foliar epiphytic extracts, and 28 were detected only in foliar epiphytic extracts (Additional file 1: Table S1). A total of 1271 metabolites were detected in flowers and floral epiphytic extracts, 194 (including aconitic acid and L-ornithine) were detected in flowers but not in their epiphytic extracts, and 28 (including adenosine and glycerol 3-phosphate) were detected only in floral epiphytic extracts (Additional file 1: Table S3).

### Effects of antibiotic treatment

All metabolites detected in leaves were found in control and in treated samples. The antibiotic treatment caused a shift in the concentrations of 118 of the 1277 (9.2 %) metabolites detected in leaves (Additional file 1: Table S4). The concentrations of 55 metabolites (including secondary metabolites such as caffeic acid) increased after treatment, and the concentrations of the other 63 metabolites decreased.

All except two of the detected metabolites in the foliar epiphytic extracts were detected in both control and treated samples. The concentrations of 133 of the 1132 (11.8 %) detected metabolites changed after the antibiotic treatment (Additional file 1: Table S5). The concentrations of 33 metabolites increased after the treatment, including d-tocopherol, glucose, a non-determined disaccharide, a non-determined hexose, raffinose pentahydrate-maltotriose, and glutamine (Additional file 1: Table S5).

The antibiotic treatment affected 97 of the 1271 (7.6 %) metabolic variables detected in the flowers (Additional file 1: Table S6). All of these metabolites were found in control and treated samples, except for two that were in the control but not the treated samples. The concentrations of 25 compounds (including a non-determined pentose, pyridoxine, loganin, catechin, threonine, phenylalanine, saponarin, and citrate) were higher in the antibiotic-treated than in the control plants (Additional file 1: Table S6).

The antibiotic treatment affected 74 of the 1271 (7.6 %) metabolites detected in the floral epiphytic extracts (Additional file 1: Table S7). All of these metabolites were found in the control and treated samples, except three metabolites that were in control but not the treated samples. Pyrogallol was present in treated but not control samples. The concentrations of six unidentified metabolites (X254, X92, X1338, X1576, X1329, and X1068) were higher in antibiotic-treated than control plants (Additional file 1: Table S7).

**Fig. 1:**
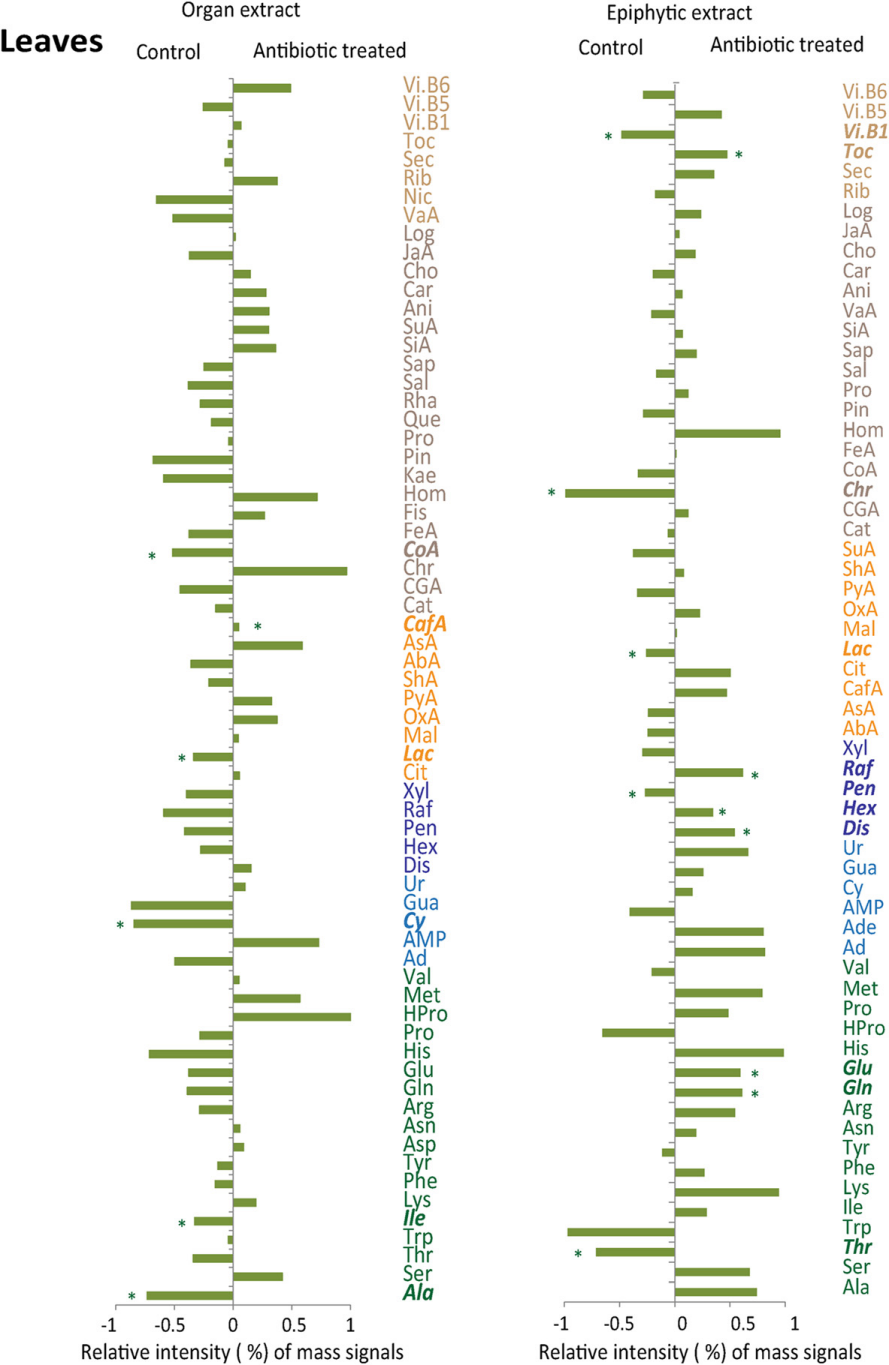
Differences between the standardized signal intensities of the identified metabolites in the LC-MS profiles of the antibiotic-treated and control leaves. The various metabolomic families are represented by different colors: green, amino acids; yellow, compounds associated with the metabolism of amino acids and sugars; cyan, nucleotides; brown, terpenes and phenolics; dark blue, sugars; dark brown, others. Metabolites: amino acids: Glu, glutamic acid; Asp, aspartic acid; Ala, alanine; Arg, arginine; Asn, asparagine; Gln, glutamine; His, histidine; HPro, hydroxyproline; Ile, isoleucine; Lys, lysine; Met, methionine; Phe, phenylalanine; Pro, proline; Ser, serine; Thr, threonine; Trp, tryptophan; Tyr, tyrosine. Nucleobases: Ad, adenine; Ur, uracil. Nucleosides: Ade, adenosine; Cy, cytidine; Gua, guanosine; Ur, uridine. Nucleotide: AMP, adenosine monophosphate. Compounds associated with the metabolism of amino acids and sugars: Cit, citric acid; Lac, lactic acid; Mal, malic acid; OxA, oxaloacetic acid; PyA, pyruvic acid; ShA, shikimic acid; SuA, succinic acid; AbA, abscisic acid (ABA); AsA, ascorbic acid (vitamin C); Cat, catechin. Others: Ani, adonitol (ribitol); Toc, d-tocopherol; JaA, jasmonic acid; Vi. B6, pyridoxine (vitamin B6); Rib, riboflavin (vitamin B2, formerly vitamin G); Vit, vitexin; Car, carvone; Sec, secologanin; Log, loganin; Cho, choline; Nic, nicotine; Vi. B5, pantothenic acid (vitamin B5); Vit. B6p, pyridoxine (vitamin B6); Vi. B1, thiamine (vitamin B1). Terpenes and phenolics: CafA, caffeic acid; CGA, chlorogenic acid; Chr, chrysin; CoA, coumaric acid; Pin, d-pinitol; FeA, ferulic acid; Hom, homoorientin; Kae, kaempferol; Pro, protocatechuic acid; Que, quercetin; Rha, rhamnetin; Sap, saponarin; SiA, sinapinic acid; Sal, sodium salicylate; VaA, vanillic acid; Fis, fisetin; Rhap, rhamnetin. Sugars: Dis, disaccharides; Hex, hexoses; Pen, pentoses; Raf, raffinose pentahydrate - maltotriose; Xyl, xylitol - arabitol. Asterisks and bold italic text indicate statistical significance (*P* < 0.05) in one-way ANOVAs

The concentration of only one identified metabolite (caffeic acid) was higher in leaves after the antibiotic treatment, whereas the concentrations of five identified metabolites were lower (Fig. 1). The antibiotic treatment caused the decrease of the concentrations of acetyl-CoA and some of the related amino acids such as alanine. The concentrations of all amino acids involved in the ketoglutarate pathway also tended to decrease, as did the concentration of lactate. In contrast, the concentrations of the amino acids glutamic acid and glutamine involved the ketoglutarate pathway tended to increase in the foliar epiphytic extracts after antibiotic application. Concentrations of vitamin B5 and some hexoses increased, while concentrations of vitamin B1 and pentoses decreased in the foliar epiphytic extracts under antibiotic treatment.

**Fig. 2:**
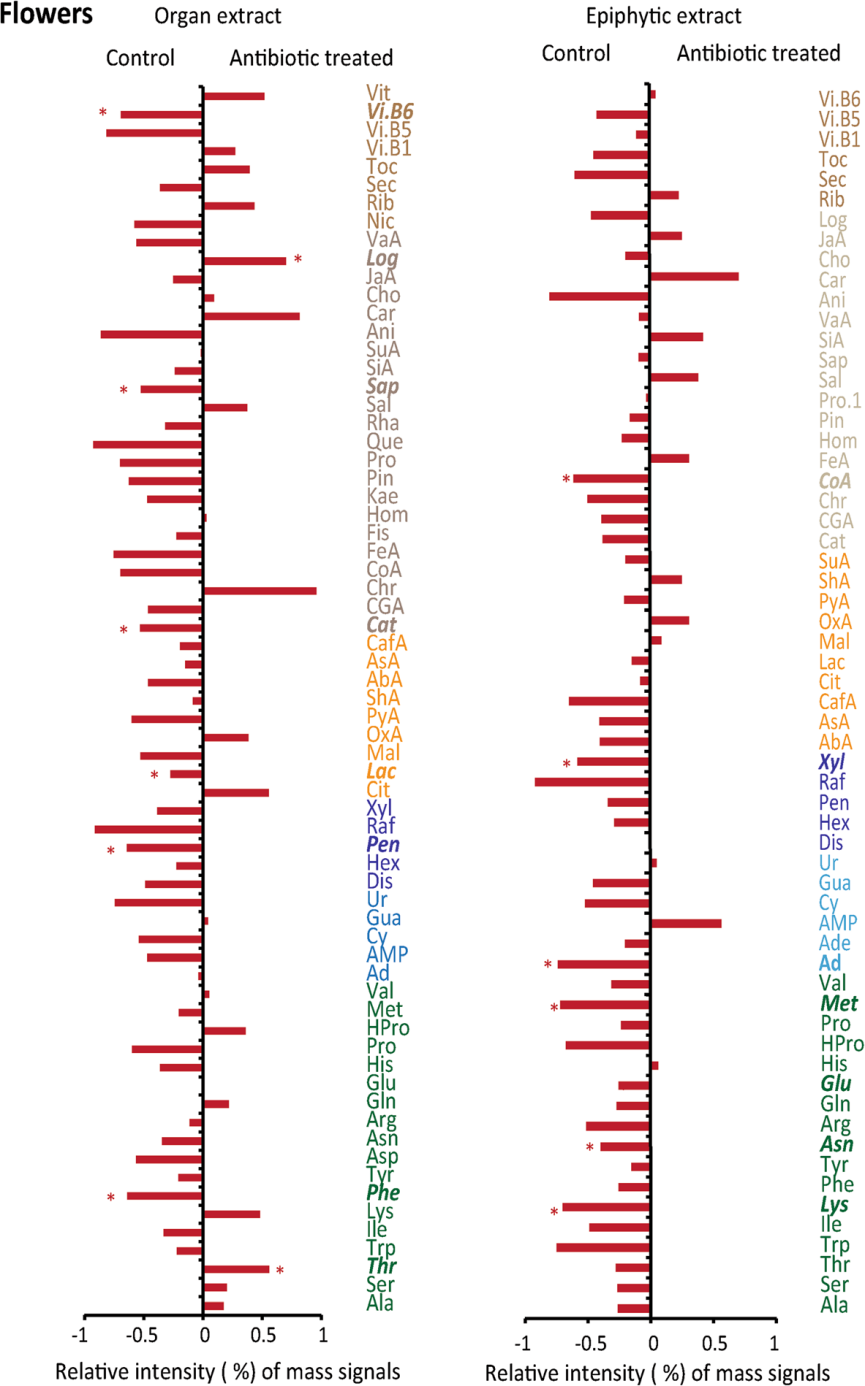
Differences between standardized signal intensities of the identified metabolites in the LC-MS profiles of the antibiotic-treated and control flowers. Variables are colored and labelled as described for Fig. 1. Asterisks and bold italic text indicate statistical significance (*P* < 0.05) in one-way ANOVAs

The effects of the antibiotic treatment on the identified metabolites were even stronger in flowers and in floral epiphytic extracts, with a general trend towards lower concentrations (Fig. 2). The concentration of only one identified metabolite, the iridoid loganin, increased after antibiotic treatment, whereas the concentrations of most of the other identified secondary compounds such as terpenes and phenols and sugars clearly tended to decrease. Concentrations decreased significantly for phenylalanine but tended to increase for the amino acids associated to the pyruvate pathway (serine, alanine, glycine, and threonine, the latter significantly) in the floral metabolomic profile after antibiotic application. The concentrations of the identified metabolites in the floral extracts did not increase significantly after the antibiotic application, but the concentrations of the identified sugars and amino acids tended to decrease.

### Multivariate analyses

**Fig. 3:**
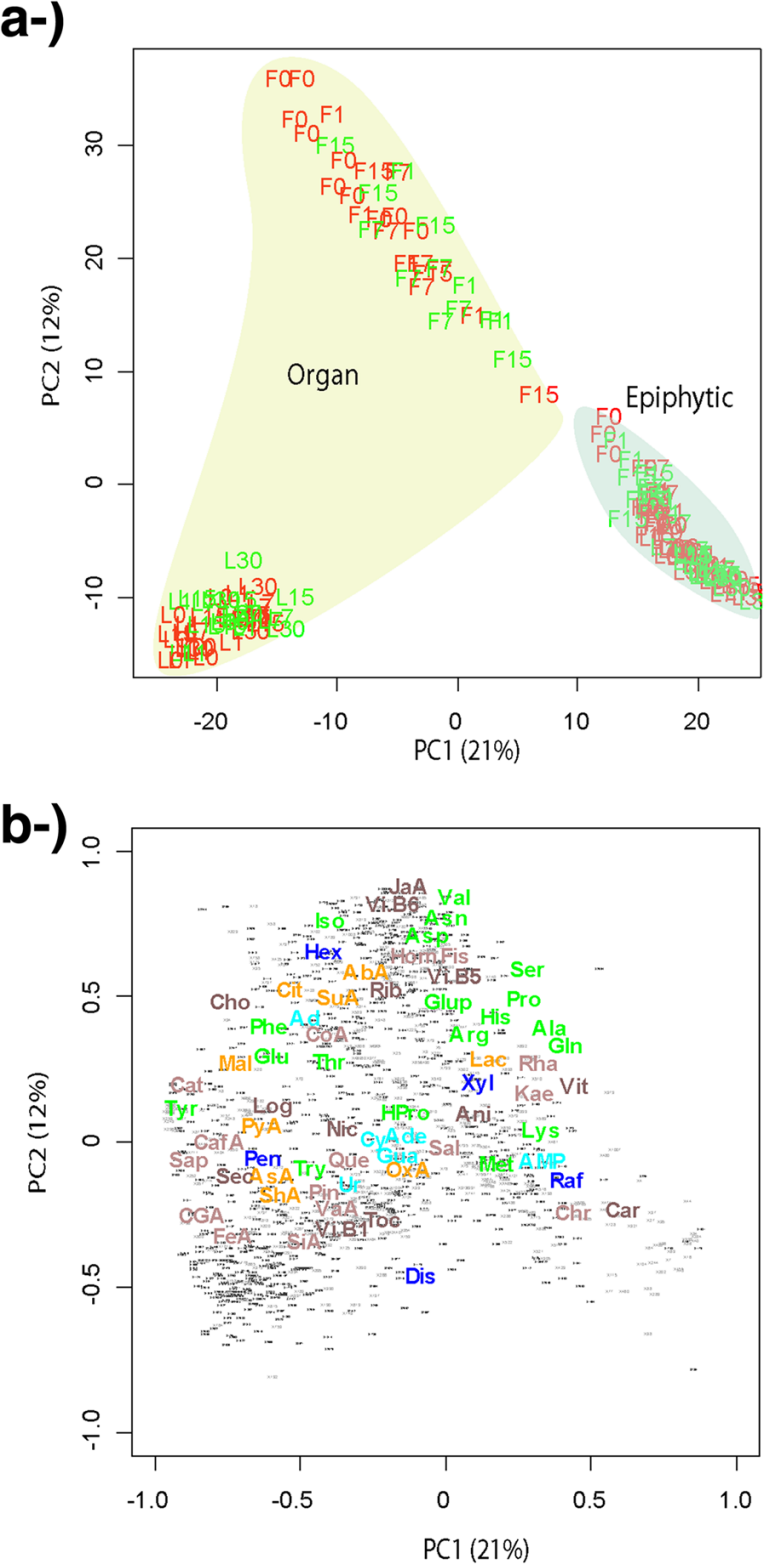
Case scores (**a**) and metabolite loading (**b**) of the PCA conducted with the variables of the metabolomes. Letters indicate different organs: F, flowers; L, leaves) and colors indicate different treatments (green, control; red, antibiotic treated). Numbers indicate the day the samples were collected (0 without treatment and 1, 7, 15, and 30 days after treatment). Variables are colored and labeled as described for Fig. 1

**Fig. 4:**
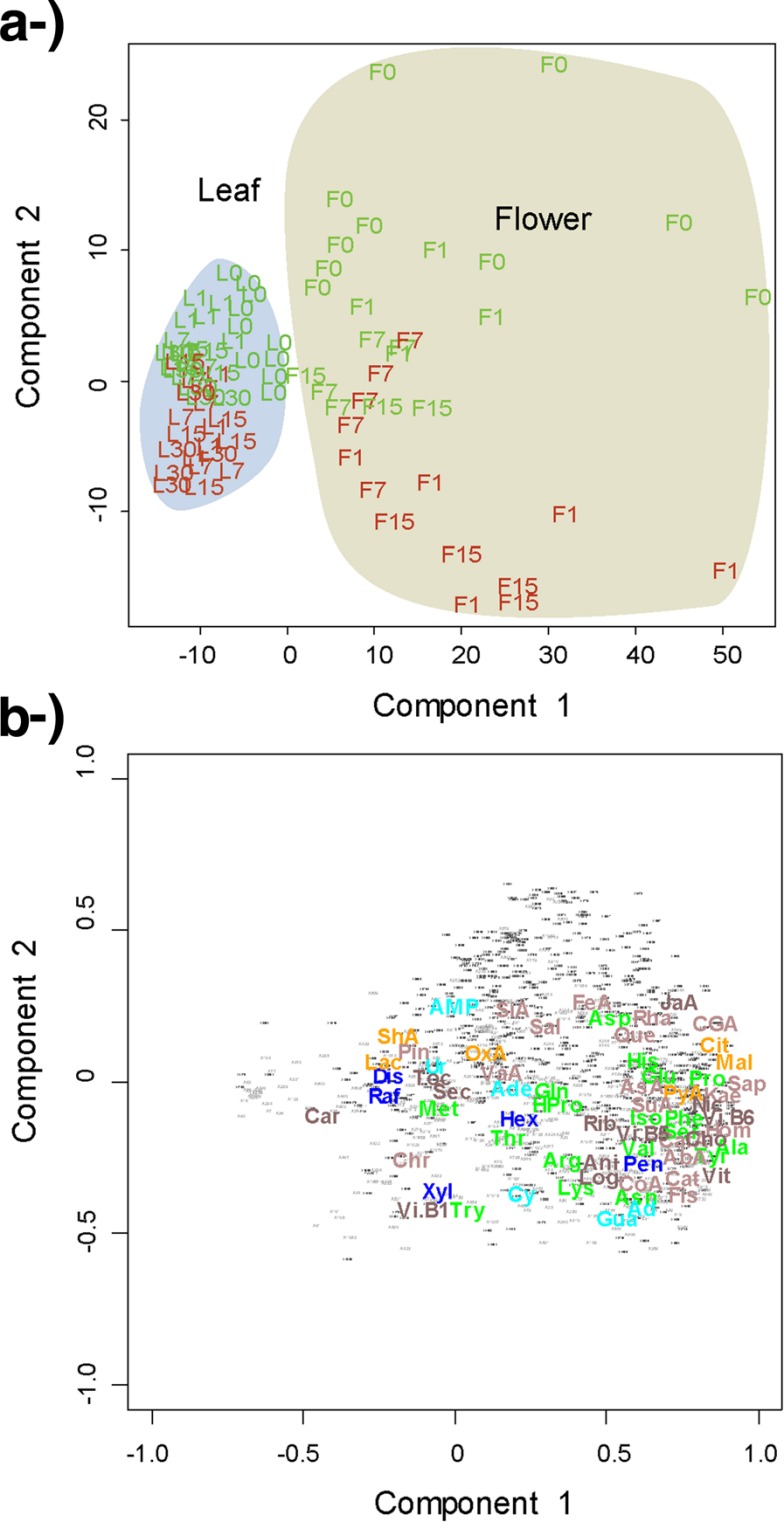
Component 1 *vs* component 2 of the partial least squares discriminant analysis (PLS-DA) of the changes of the metabolomes of the epiphytic extracts in response to the antibiotic treatment. Case scores are represented in (**a**-) and metabolite loading in (**b**-). Letters indicate different organs (F, flowers; L, leaves), and colors indicate different treatments (green, control; red, antibiotic treated). Numbers indicate the day the samples were collected (0 without treatment and 1, 7, 15, and 30 days after treatment). Variables are colored and labeled as described for Fig. 1

**Fig. 5:**
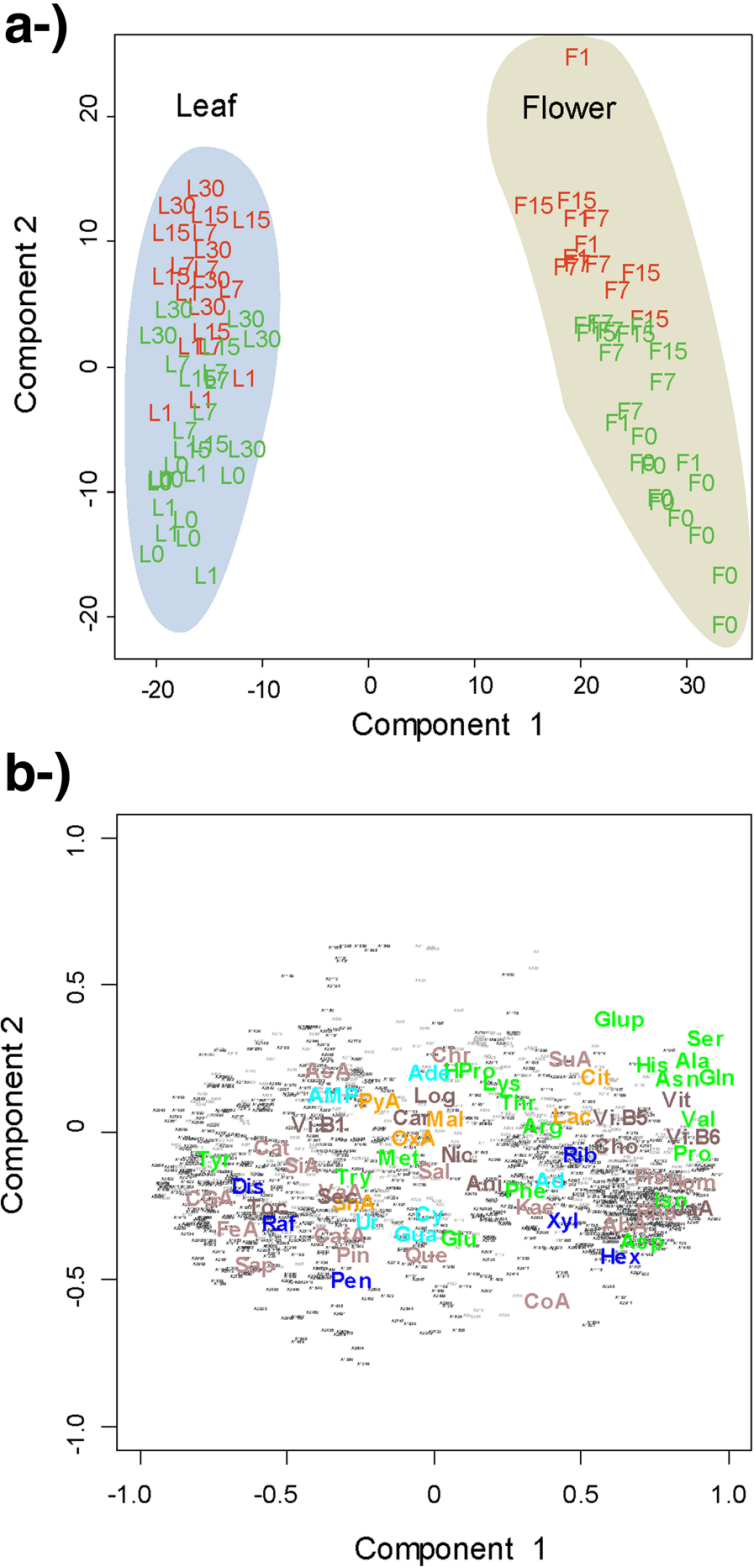
Component 1 *vs* component 2 of the partial least squares discriminant analysis (PLS-DA) of the changes of the metabolomes of the plant organs extracts in response to the antibiotic treatment. Case scores are represented in (**a**-) and metabolite loading in (**b**-). Letters indicate different organs (F, flowers; L, leaf), and colors indicate different treatments (green, control; red, antibiotic treated). Numbers indicate the day that samples were collected (0 without treatment, 1, 7, 15, and 30 days after the treatment). Variables are colored and labeled as described for Fig. 1

The metabolic profiles clearly differed between the plant organs and their microbial epiphytic communities (Fig. 3). The PCAs (Principal Components Analysis) of all the metabolic data analysis showed that the samples of epiphytic microbial and organ metabolic profiles were separated along PC1 for both organs. The changes epiphytic in metabolome structure between epiphytic community and the correspondence internal organ were more significant between the leaves and their epiphytic extracts than the observed in flowers. The metabolic profiles of the flowers and leaves samples were separated along PC2 for both the organs and their epiphytic microbial communities (Figs. 3, 4 and 5). The PERMANOVA analysis confirmed these results, indicating different metabolomes between the organs and the epiphytic extracts (pseudo-F = 361; *P* < 0.001). The overall metabolomes also differed significantly depending on the organ (pseudo-*F* = 159; *P* < 0.001) date of sampling (pseudo-*F* = 22.7; *P* < 0.001), individual plant (pseudo-*F* = 6.61; *P* < 0.001), and antibiotic treatment (pseudo-*F* = 5.00; *P* < 0.01) (Table 1). Some two-level interactions between factors were also significant: individual plant with plant organ and epiphytic environment (pseudo-*F* = 2.23; *P* < 0.05), date of sampling with plant organ (pseudo-*F* = 2.47; *P* < 0.05), date of sampling with organ and epiphytic environment (pseudo-*F* = 6.44; *P* < 0.01), and plant organ with organ and epiphytic environment (pseudo-*F* = 108; *P* < 0.001). The interaction between treatment with organ and epiphytic environment, however, was only marginally significant (pseudo-*F* = 1.74; *P* < 0.1).

**Table 1:** PERMANOVA results

	N-1	F.Model	R2	Pr( > f)
**PL(plant)**	**9**	**6.11**	**0.01145**	**<0.0001**
**DA(date)**	**4**	**22.71**	**0.02379**	**<0.0001**
**PA(Flowers and leaves)**	**1**	**159.3**	**0.018847**	**<0.0001**
**G(organ and epiphytic)**	**1**	**361.23**	**0.43018**	**<0.0001**
**TRT(Antibiotic treatment)**	**1**	**5**	**0.00237**	**0.007**
*PL:DA*	*35*	*2.23*	*0.02106*	*0.08*
*PL:PA*	*9*	*1.96*	*0.01253*	*0.056*
**PL:G**	*9*	*2.22*	*0.01033*	*0.028*
PL:TRT	9	1.83	0.00809	0.13
**DA:PA**	**4**	**2.47**	**0.00757**	**0.026**
**DA:G**	**4**	**6.4**	**0.01924**	**0.002**
DA:TRT	2	1.35	0.00069	0.25
**PA:G**	**1**	**108.99**	**0.00065**	**<0.0001**
PA:TRT	1	1.5	0.00058	0.15
*G:TRT*	*1*	*1.74*	*0.00048*	*0.098*
Residuals	168	4.616		
Total	268	51.043		

Bold type indicates significant effects (*P* < 0.05). Italics type indicates marginally significant effects (*P* < 0.1)

In the plot formed by the two first axes of the PCA, the metabolic profile of the flowers showed a higher proportion of most amino acids, some sugars such as hexoses and xylitol-arabitol, and some secondary metabolites such as terpenes and phenols (Fig. 3). The metabolic profile from the two first PCA axes of the leaves showed higher concentrations of some metabolites associated to the Krebs cycle such as malic acid, pyruvate, chlorogenic acid, quercetin, and oxaloacetate; nitrogenous bases such as adenosine, guanosine, and uridine; and most secondary metabolites (Fig. 3).

When all the data was analyzed at once, the metabolic profiles from the two first PCA axes of the plant organs showed higher proportions of most amino acids, some sugars such as hexoses and pentoses, and some secondary metabolites such as terpenes and phenols than the corresponding epiphytic communities. The epiphytic communities showed higher proportions of some amino acids such as lysine and methionine, some sugars such as raffinose, some secondary compounds such as chrysin and carvone, and of AMP than plant organs. The epiphytic communities showed notably higher concentrations of lactate (Fig. 3).

The epiphytic metabolomes were less variable than the organ metabolomes (Fig. 3). Epiphytic metabolomic variability was much less significant and lower between leaves and their epiphytic extracts than between flowers and their epiphytic extracts. The coefficients of variation of the PC2 scores were 16 % for leaves and 58 % for flowers.

The effect of the antibiotic treatment was greater in the epiphytic environment than in the corresponding organs metabolic profile, despite it was also significant in them. The PERMANOVA indicated overall shifts in the metabolomic profiles of leaves and flowers due to the treatment, being flowers more sensitive to the treatment than leaves. The decrease in lactate concentrations due to the antibiotic treatment was general in all samples, of organs and epiphytic extracts. Also, the antibiotic treatment caused the decrease of the concentrations of citraconic acid in the foliar and floral epiphytic communities and the presence of pyrogallol in the floral epiphytic community.

## Discussion

### Effects of suppression of the epiphytic community on metabolic profiles

The effect of the antibiotic treatment on the metabolic profiles was evident in both the epiphytic communities and the plant organs. The observed shifts in the metabolic profiles of both internal plant organs and their epiphytic communities emphasized the importance of the microbiota in the metabolic function of plants. This study is, to the best of our knowledge, the first work that demonstrates a shift in the global metabolomic expression of a plant due to the suppression of its microbial community. The antibiotics were applied to all plant surfaces with an expected direct impact on the surface of the plant, but this antibiotic application also affected the internal plant organ metabolome, suggesting some levels of impact on endophyte microbial communities. These results were consistent with previous studies, which reported that some target metabolites or specific functions of a plant are related to microbial communities living into plant organs [9, 11, 35]. Consistently with all this, some studies have shown that in internal plant organs it exists a wide microbial endophyte community, which is apparently not related to parasitism or symbiosis functions [41, 42].

The concentration of citraconic acid in the epiphytic extracts decreased after the antibiotic treatment. Citraconic acid is produced by microorganisms [43], particularly by the degradation of isoprenoid compounds [44]. This result was also consistent with the change in the molecular composition of terpene emissions observed after antibiotic treatment [45]. The decrease in terpene emissions reported by Peñuelas et al. [45] was likely due to the impact of the antibiotics on the floral epiphytic microbiota, reducing *de novo* biosynthesis [46–48] or biotransformation [3, 14, 49–51]. Terpene biosynthesis is common in microbial metabolism, but only a few bacterial and fungal *TPS* genes have yet been reported [51, 52], likely due to the low identities of the amino acid sequences of these enzymes compared with those in eukaryotes [14]. For example, the presence of some epiphytic microbes can induce an immune response by the plant and the subsequent emission of defensive terpenes from flowers to control their microbial communities [10].

Another interesting result was the decrease in the proportion of lactate in both organs and epiphytic extracts after the antibiotic treatment, suggesting an inhibition of fermentation. In fermentation, electrons are transferred from reduced substrates to oxidized intermediates to generate reduced fermentation products such as lactate [53, 54]. Our results, thus, strongly suggest that the anaerobic and/or facultative anaerobic bacteria are present in significant proportions in the phyllosphere and also in the endophytic microbial communities of *S. nigra*. A quantitative and qualitative study of the phyllospheric microflora of *Aloe vera* showed that bacteria and yeast densities were relatively high, and bacteria were represented mainly by facultative anaerobic genera, dominated by Enterobacteriaceae [55].

The metabolite pyrogallol appeared in the floral epiphytic community after the antibiotic treatment. Pyrogallol is a phenolic compound synthesized in plants by the shikimate pathway and is believed to function as a defensive agent against invading microbes and as a signal molecule in plant interactions with pathogens [56]. The presence of microorganisms may thus have either inhibited the synthesis of pyrogallol or biotransformed it, or, alternatively, the plant may have produced more when the potentially defensive role of the microbes was suppressed.

The antibiotic treatment caused the decrease of the concentrations of acetyl-CoA and its derived amino acid isoleucine in the leaves. Isoleucine is a precursor in the synthesis of several secondary compounds, many of which decreased in concentration after antibiotic application. The concentrations of the amino acid precursors of the ketoglutarate-synthesis pathway tended to decrease in the leaves and to increase in the foliar epiphytic extracts in response to antibiotic application. The concentrations of most of the detected metabolites generally decreased in flowers after antibiotic application, though the concentration of the others increase, such is the case of the amino acids associated to the pyruvate-synthesis pathway. These results indicated that the impacts of the antibiotic treatment on the metabolic profile of the internal plant were also significant, even though the impacts were stronger on the metabolic profile of the epiphytic. As previously mentioned, we observed that the concentration of lactate increase in the internal plant organs after being treated by the antibiotic. The environment of apoplasts (the intercellular space surrounding plant cells) would be competitive for oxygen. Fermentation is favored in these anaerobic situations, and high concentrations of lactate would be produced by the microorganisms. Our results further suggest that 1) the antibiotics penetrated the interiors of the leaves and 2) the antibiotic treatment may have had a direct effect on plant metabolism [18, 19]. Unfortunately, this study did not allow to determine the cause of these effects; for instance, if they are the results of the internal microbial communities that respond to the antibiotic present in the plant cells. We cannot exclude also a potential antibiotic-induced damage to the plant tissues that further affects metabolic profile but no visual symptoms were detected nor there changes in exchange of CO_2_ and water.

### Metabolic profile differences between leaves and flowers

The two plant organs studied had different metabolic profiles, with flowers having the more variable profile. The differences between these two organs explained the 58% of the total variance of the metabolomes. These results were consistent with those of previous studies showing different metabolic profiles among organs of the same plant, for example between shoot and root biomass in grasses [57, 58]. Functional specialization among plant organs is thus high and should be taken into account in ecometabolomic studies, because the metabolic functional response and the relationships between environmental variables and shifts in plant metabolomes can be very different, and even opposite, depending on the organ [57, 58].

*S. nigra* is pollinated by insects (mainly hoverflies), so the higher concentrations of some sugars and amino acids in the flowers than the leaves may be related to nectar synthesis and to spore and gamete formation. Floral secondary metabolites such as terpenes and phenols are produced to attract insects [59–61]. Leaves are the main photosynthetic tissue in trees and thus the site of primary production of the main biomolecules. This is consistent with the higher concentrations in leaves than in flowers of some metabolites, associated with the Krebs cycle (malic acid, pyruvate, chlorogenic acid, quercetin, and oxaloacetate, and the nitrogenous bases adenosine, guanosine, and uridine). Foliar secondary metabolites such as terpenes and phenols are produced in defensive reactions [62, 63].

### Organ versus epiphytic metabolism

The high percentage of the detected metabolomic variables that differed significantly between the organs and epiphytic extracts, and the low percentage of compounds in the organs but not in the epiphytic extracts, or in the epiphytic extracts but not in the organs, indicated similar metabolomes, (with many common metabolites but with different relative proportions of these common metabolites), as indicated by the PCA.

The complex metabolic profiles were more similar between the two epiphytic communities than between the corresponding metabolic profiles of the organs. The metabolism of the epiphytic extracts may thus be much more conservative and homeostatic than that of the organs, and the metabolism of the flowers may be more variable than that of the leaves. The metabolomes of the two epiphytic microbial communities, however, differed significantly in some aspects.

Plant organs have complex functions, and the leaves and flowers in our study had higher proportions of most amino acids, some sugars such as hexoses and pentoses, and some secondary metabolites such as terpenes and phenols than the corresponding epiphytic communities. The large variety of these compounds are thus provided by the plants and not by the microorganisms [20, 64, 65]. The epiphytic community, however, had a higher proportion of some amino acids such as lysine and methionine, some sugars such as raffinose. In especial, the increase of the concentration of some secondary compounds such as chrysin and carvone indicates that the microorganisms play an important role in the plant and can produce some metabolites for the immune response of the plant [10].

## Conclusions

The antibiotic treatments changed the phyllospheric production of metabolites thus indicating a key functional importance of microorganism in plant epiphytic environment. We also observed that the antibiotic penetrated the plant organs, and their effects also suggested an important role of the microbiota in the metabolome of the organs. The differences in the metabolomic compositions between flowers and leaves were greater than the difference between their corresponding epiphytic environments. of internal plant organ.

The concentrations of citraconic acid, acetyl-CoA, isoleucine, and several secondary compounds such as terpenes and phenols in the epiphytic extract decreased after the antibiotic treatment. The metabolite pyrogallol appeared in the floral epiphytic community only after the treatment. The concentrations of the amino acid precursors of the ketoglutarate-synthesis pathway tended to decrease in the leaves and to increase in the foliar epiphytic extracts. The proportion of lactate interestingly decreased in both the organs and their epiphytic extracts after the antibiotic treatment, suggesting a decrease in fermentation when the microbial populations were suppressed.

Our results showed that microbial communities can thus play a role in the epiphytic and internal metabolomes of plant tissues and organs and could have more complex and frequent mutualistic, saprophytic, and/or parasitic relationships with internal morphological structures than currently known. A clear classification of these relationships can be difficult, particularly in terms of discriminating between commensals and mutualistic symbionts [16]. This study highlights the large complexity of the phyllosphere, the existence of internal microbial communities, and the strong relationships between the structure and function of the internal and external plant metabolomes. These results thus warrant further study of the specific relationships between plants and the microbial communities living on and in them.

## Methods

Experimental research was conducted on nursery plants complying with national guidelines. We used 24 year-old potted *Sambucus nigra* L. plants, grown in a nursery (Tres Turons S.C.P., Castellar del Vallès, Catalonia, Spain) in 15-L pots with a 2:1 peat:sand substrate and maintained with regular irrigation under outdoor Mediterranean ambient conditions to ensure that the substrate was held at field capacity throughout the experimental period. Ten of the plants were fumigated during 1 min with 1600 ppm streptomycin, 400 ppm oxytetracycline, and 200 ppm chloramphenicol in 50 mL of H_2_O with 1 % glycerol to eliminate the floral and foliar epiphytic microbiota. These antibiotics are used in agriculture mainly in prophylactic treatments [66]. The other 10 plants were kept as control plants and were sprayed with 50 mL of H_2_O with 1 % glycerol but without antibiotics.

### Collection and preparation of tissue samples

Samples of leaves and flowers were collected in spring before treatment and after 1, 7, 15, and 30 days of fumigation. The samples were washed in glasses with water for 2 min. The water was immediately frozen at −80 °C, and the flowers and leaves were lyophilized to avoid leaching. The experimental design contained a total of 200 samples: five sample-collection days, two organs per plant (leaves and flowers), two fractions (epiphytic and plant), two treatments (fumigated and unfumigated), and five replicates. The sample preparation is described in detail by Rivas-Ubach et al. [67]. Briefly, the flowers and leaves were frozen immediately in liquid nitrogen and then lyophilized and stored in plastic cans at −80 °C. The samples were then ground with a ball mill (Mikrodismembrator-U, B. Braun Biotech International, Melsungen, Germany) at 1700 rpm for 4 min, producing a fine powder that was stored at −80 °C. The metabolomes of the solid contents of the lyophilized solutions from the washed floral and foliar surfaces were extracted by the same methodology as for the organs. See the supplementary material of Gargallo-Garriga et al. [58] for details.

### Analysis by liquid chromatography-mass spectrometry (LC-MS)

The LC-MS platform (all from ThermoFisher Scientific, San Jose, CA, unless otherwise noted) consisted of an Accela U-HPLC system with quaternary pumps, an HTC PAL autosampler (CTC Analytics AG, Zwingen, Switchland), a Keystone hot pocket column heater, and an Exactive Orbitrap mass spectrometer controlled by Xcalibur 2.1. Reversed-phase LC separation used a Synergy Hydro-RP column (100 × 2 mm, 2.5 μm particle size, Phenomenex, Torrance, CA) with the ion-pairing agent tributylamine in the aqueous mobile phase to enhance retention and separation. The LC used a column with a small particle size (2.5 μm instead of 4 μm) to reduce peak widths and expedite analysis. The total run time was 25 min, and the flow rate was 200 μL/min. Solvent A was 97:3 water:methanol with 10 mM tributylamine and 15 mM acetic acid; solvent B was methanol. The gradient was 0 min, 0 % B; 2.5 min, 0 % B; 5 min, 20 % B; 7.5 min, 20 % B; 13 min, 55 % B; 15.5 min, 95 % B; 18.5 min, 95 % B; 19 min, 0 % B; 25 min, 0 % B, and the column was then washed and stabilized for 5 min before the next sample was injected. Other LC parameters were: autosampler temperature, 4 °C; injection volume, 10 μL; and column temperature, 25 °C. HESI (heated electrospray ionization) was used for MS detection. All samples were injected twice, once with the ESI operating in negative ionisation mode (^−^H) and once in positive ionisation mode (^+^H). The Orbitrap mass spectrometer was operated in FTMS (Fourier Transform Mass Spectrometry) full-scan mode with a mass range of 50–1000 m/z and high-mass resolution (60,000). The resolution and sensitivity of the spectrometer were monitored by injecting a caffeine standard after every 10 samples, and the resolution was further monitored with lock masses (phthalates). Blank samples were also analyzed during the sequence. The assignment of the metabolites was based on standards, with the retention time and mass of the assigned metabolites in both positive and negative ionisation modes.

### Statistical analyses

The LC-MS data were analyzed by univariate and multivariate statistical analyses. Permutational multivariate analyses of variance (PERMANOVAs) [68] were conducted using the Euclidean distance, with organ (flowers and leaves), treatment (control and antibiotic treated), date (pre-treatment, 1, 7, 15, and 30 days after antibiotic treated), and fraction (epiphytic and plant) as fixed independent factors and individuals as random independent factors. Multivariate ordination principal component analyses (PCAs) (based on a matrix of correlations) and partial least squares discriminant analyses (PLS-DAs) were also performed to detect patterns of sample ordination in the metabolomes. The PCAs and PLS-DAs initially analyzed the HPLC-MS data with the various detected and quantified metabolites as variables and different samples as cases. The detailed analysis of the data from the above set of experiments allowed the analysis of the differences between the metabolic profiles and enabled the identification of clusters, groups, outliers, and, in general, the differences between the metabolic profiles of the plant tissues and between those of the tissues and the phyllosphere with and without antibiotic treatment (Fig. 1). A Kolmogorov-Smirnov (KS) test was performed on each variable for normality. All identified and unidentified metabolites were normally distributed. The PERMANOVA, PLS-DAs, ANOVAs, post-hoc tests, and KS tests used R software (R Development Core Team 2008) and were performed to detect shifts in both the metabolomes and individual metabolites and in the variables controlling them.

### Availability of supporting data

Data is available at demand of everybody interested in globalecology.creaf.cat.

## Additional file

Additional file 1:
**Table S1.** One-way ANOVAs of identified metabolites in leaf organ and epispheric. **Table S2** One-way ANOVAs of identified metabolites in flowers organ and epispheric. **Table S3** One-way ANOVAs of identified metabolites in in leaf organ in different antibiotic treatment within the plants receiving control levels of water in leaf. **Table S4** One-way ANOVAs of identified metabolites in identified in leaf epispheric in different antibiotic treatment within the plants receiving control levels of water in leaf. **Table S5** One-way ANOVAs of identified metabolites in in flowers organ in different antibiotic treatment within the plants receiving control levels of water in leaf. **Table S6** One-way ANOVAs of identified metabolites in in flowers epispheric in different antibiotic treatment within the plants receiving control levels of water in leaf. **Table S7** Abbreviation, family and the real name of the metabolites detected. (PDF 3529 kb)

## Abbreviations

ESI: Electrospray ionization
FTMS: Fourier transform mass spectrometry
HESI: Heated electrospray ionization
LC-MS: Liquid chromatography-mass spectrometry
PCA: Principal components analysis
PERMANOVAs: PERmutational ANOVAs
PLS-DA: Partial least squares discriminant analysis
TPS: Terpene synthase
