# Supplementary material for: Shifts in plant foliar and floral metabolomes in response to the suppression of the associated microbiota
Source: BMC Plant Biol. 2016 Apr 6;16:78. doi: 10.1186/s12870-016-0767-7 (PMC4822282; doi:10.1186/s12870-016-0767-7)
Supplement: Additional file 1: — Table S1. One-way ANOVAs of identified metabolites in leaf organ and epispheric. Table S2 One-way ANOVAs of identified metabolites in flowers organ and epispheric. Table S3 One-way ANOVAs of identified metabolites in in leaf organ in different antibiotic treatment within the plants receiving control levels of water in leaf. Table S4 One-way ANOVAs of identified metabolites in identified in leaf epispheric in different antibiotic treatment within the plants receiving control levels of water in leaf. Table S5 One-way ANOVAs of identified metabolites in in flowers organ in different antibiotic treatment within the plants receiving control levels of water in leaf. Table S6 One-way ANOVAs of identified metabolites in in flowers epispheric in different antibiotic treatment within the plants receiving control levels of water in leaf. Table S7 Abbreviation, family and the real name of the metabolites detected. (PDF 3529 kb) [file 12870_2016_767_MOESM1_ESM.pdf]

Supplementary Material

Shifts in plant foliar and floral metabolomes in response to the suppression of the associated microbiota

Albert Gargallo-Garriga, Jordi Sardans, Míriam Pérez-Trujillo, Alex Guenther, Joan Llusià, Laura Rico, Jaume Terradas, Gerard Farré-Armengol, Iolanda Filella, Teodor Parella, Josep Peñuelas.

Table S1. Antibiotic trends in different plant organs during the experimental time. The "X" symbols mean that the corresponding antibiotic was detected in the corresponding day and plant organ.

Table S2 One-way ANOVAs of identified metabolites in leaf organ and epispheric.

Table S3 One-way ANOVAs of identified metabolites in flowers organ and epispheric

Table S4 One-way ANOVAs of identified metabolites in leaf organ in different antibiotic treatment within the plants receiving control levels of water in leaf**.**

Table S5 One-way ANOVAs of identified metabolites in identified in leaf epispheric in different antibiotic treatment within the plants receiving control levels of water in leaf.

Table S6 One-way ANOVAs of identified metabolites in flowers organ in different antibiotic treatment within the plants receiving control levels of water in leaf.

Table S7 One-way ANOVAs of identified metabolites in flowers epispheric in different antibiotic treatment within the plants receiving control levels of water in leaf**.**

Table S8 Abbreviation, family and the real name of the metabolites detected.

Table S1. Antibiotic trends in different plant organs during the experimental time. The "X" symbols mean that the corresponding antibiotic was detected in the corresponding day and plant organ.

			Days			
Antibiotic	Aplication	Organ	1	7	15	30
Chloramphenicol	Organ	Leaves	X	X	X	X
		Flowers	X	X	X	
	Epiphytic extracts	Leaves	X	X	X	
		Flowers	X	X	X	
streptomycin	Organ	Leaves	X	X		
		Flowers	X	X		
	Epiphytic extracts	Leaves	X	X		
		Flowers	X	X		
Oxytetracycline	Organ	Leaves				
		Flowers				
	Epiphytic extracts	Leaves	X	X	X	
		Flowers	X	X	X	

Table S2. Deconvoluted total intensities (mean ± S.D.) of all identified in leaf organ and epiphytic. The statistically significant differences between organ and epiphytic detected by Tukey's HSD post-hoc tests are indicated by bold letters ($P < 0.05$). Metabolites as in Fig. 1 caption, the X represent the unknown metabolites.

Leaf							
**Metabolites**	**Organ**			**Epiphytic**			
	**Mean**	**S.D.**		**Mean**	**S.D.**		**Pr(>F)**
Ala	608080	88710	b	2970857	433381	a	0,00
Arg	161102	57860	b	1944535	1087024	a	0,11
Asn	11273001	4619509	b	13988279	3643229	a	0,65
Asp	6111352	378513	a	0	0	b	0,00
Gln	320989	32400	b	21842404	6118521	a	0,00
Glu	5260092	445800	a	953571	259338	b	0,00
Glup	26483866	3824373	a	23687751	4606656	b	0,64
His	103058	24218	b	518767	224263	a	0,07
HPro	1787	1499	b	42114	6554	a	0,00
Iso	66304950	5023551	a	5453917	3577516	b	0,00
Lys	137841	41414	b	809205	742535	a	0,37
Met	328729	96576	b	2449185	1484102	a	0,16
Phe	77295029	5281840	a	5421191	2880184	b	0,00
Pro	7893161	1397210	b	10945548	1224818	a	0,11
Ser	737298	258349	b	3601027	1456982	a	0,06
Thr	26293768	2820350	a	5640909	2114969	b	0,00
Try	79880069	13365419	a	562351	163524	b	0,00
Tyr	336661237	3768038	a	41668671	5063853	b	0,00
Val	31126179	2641375	b	37266142	7132504	a	0,42
Ad	55380791	14138332	a	357297	195472	b	0,00
Ade	0	0	b	491953	411917	a	0,24
AMP	218497	92587	b	678504	234121	a	0,07
Cy	109237	50042	a	38082	10814	b	0,17
Gua	1480092	911663	a	50928	15093	b	0,12
Ur	1025442	404939	a	48286	14082	b	0,02
Dis	6818610	598329	a	5229512	840120	b	0,13
Hex	27206522	3369976	a	9894239	814257	b	0,00
Pen	4725204	605542	a	433676	39361	b	0,00
Raf	37052	7295	b	22545987	5061247	a	0,00
Xyl	3498087	585723	b	3605594	743752	a	0,91
AbA	1185314	155821	a	35218	13144	b	0,00
Ani	1637618	579697	a	1251811	485065	b	0,61
AsA	9369389	1696343	a	201996	59064	b	0,00
CafA	7682645	693849	a	48221	20348	b	0,00
Car	505615	194329	b	9989737	1806789	a	0,00
Cho	394673069	18189622	a	30811481	2154768	b	0,00
Cit	60008580	5569428	a	7749005	2984084	b	0,00
JaA	83464	23742	a	8972	4056	b	0,00

Lac	9342772	1211187	b	16791384	2732532	a	0,02
Log	1861718	277892	a	38023	7554	b	0,00
Mal	318524792	20947015	a	34105105	6368660	b	0,00
OxA	176157	98098	a	52987	10282	b	0,22
PyA	7183022	849725	a	127877	36248	b	0,00
ShA	3623163	532514	a	623109	96028	b	0,00
SuA	44728936	4392685	a	8364015	1132702	b	0,00
Cat	56827081	960368	a	2581987	475190	b	0,00
CGA	773350753	36238554	a	15193314	2742079	b	0,00
CGAp	288660533	9984471	a	753128	344094	b	0,00
Chr	52708	33723	b	160925	78139	a	0,21
CoA	3492647	500600	a	240766	60184	b	0,00
FeA	45530342	3775678	a	1760266	470260	b	0,00
Fis	218592	73147	a	0	0	b	0,00
Hom	3026063	905191	a	6621	4502	b	0,00
Hom.1	2431043	222165	a	400446	121469	b	0,00
Kae	147738	41717	a	471	343	b	0,00
Pin	1927629	428222	a	224054	47859	b	0,00
Prot	595568	77405	a	39675	6606	b	0,00
Que	3766478	973974	a	1511	697	b	0,00
Rha	17569	5526	a	0	0	b	0,00
Sal	363074	70250	a	315057	78228	b	0,65
Sap	163583485	8133430	a	1616257	372038	b	0,00
SiA	3906764	708481	a	5783	830	b	0,00
VaA	3906710	1077343	a	64207	9876	b	0,00
Nic	1412571	340956	a	0	0	b	0,00
Rib	1059734	105493	a	117084	30026	b	0,00
Sec	33382987	3436009	a	5019025	1393592	b	0,00
Toc	20462346	1018747	a	14848710	1803450	b	0,01
Vi.B1	2574199	566887	a	51937	7592	b	0,00
Vi.B5	220816	36051	a	74353	33452	b	0,00
Vi.B6	558765	108248	a	36916	9282	b	0,00
Vit	20641	7310	a	0	0	b	0,01
X1	203792975	3588958	a	40768932	3121133	b	0,00
X2	11016756	893941	a	840684	126811	b	0,00
X3	14536981	845095	a	949353	136642	b	0,00
X4	17825257	797200	b	38440704	2615409	a	0,00
X5	69715	6806	b	76378	7148	a	0,50
X6	154251517	26406445	a	124532812	14842231	b	0,33
X7	416670	285828	b	179989251	11881234	a	0,00
X8	873284515	34609391	a	133145150	31590598	b	0,00

X9	239680	64282	b	509246	347736	a	0,45
X10	83387441	1208842	a	12836657	1335680	b	0,00
X11	71492348	3200703	b	74699523	3464042	a	0,50
X13	65860141	16788833	a	59709	36845	b	0,00
X14	354940	209174	a	0	0	b	0,10
X16	136073	30532	b	68391403	3362996	a	0,00
X19	1801780	384093	a	0	0	b	0,00
X20	5660916	594933	a	1889643	220107	b	0,00
X21	9403479	802729	a	2071408	267757	b	0,00
X22	2953983658	38089382	a	751848083	45622013	b	0,00
X23	23753939	1682904	a	5126195	710665	b	0,00
X24	7907574	680100	a	1918336	258064	b	0,00
X25	668570	215490	a	1702	745	b	0,00
X26	472890576	7051227	a	87629075	6282984	b	0,00
X27	191352635	9868912	b	454356609	27495259	a	0,00
X28	0	0	b	49035	8094	a	0,00
X29	406584947	5529613	a	100174657	6402949	b	0,00
X30	6881607	983261	a	657180	79823	b	0,00
X31	45435428	16751083	a	15136150	8149276	b	0,11
X32	2208936	1415406	a	844656	685030	b	0,39
X33	3117663	1854188	a	942472	849092	b	0,29
X34	57457481	3350601	a	14578662	1597969	b	0,00
X35	164600	27736	b	6593015	593799	a	0,00
X36	58872511	12157361	b	1692469399	125011659	a	0,00
X37	370388	88792	b	17480242	2041688	a	0,00
X38	5280	3147	b	2881616	251203	a	0,00
X39	23102439	830582	a	705319	83686	b	0,00
X40	297904	50457	b	117120703	5425119	a	0,00
X41	997517002	10748140	a	178864160	14392180	b	0,00
X42	110667296	1228236	a	13951707	2072539	b	0,00
X43	9074	994	a	7906	973	b	0,40
X44	20905847	1858227	b	159729826	10192648	a	0,00
X45	63433	12173	b	29458747	1535945	a	0,00
X46	134152630	1598745	a	20131748	2242676	b	0,00
X47	38084	11648	b	590172	116744	a	0,00
X48	136393	73484	b	30927598	11221195	a	0,01
X49	170802	70287	a	152912	19726	b	0,81
X50	215158	154948	b	637955	240861	a	0,15
X52	4802300	2695341	a	1283244	1011879	b	0,23
X53	165054	43137	a	143012	18738	b	0,64
X55	395490	167298	b	4417916	1134764	a	0,00

X58	2876	1657	b	41599	12153	a	0,00
X59	4428192	425112	b	4532869	390946	a	0,86
X60	117074726	16777162	a	98816247	11608260	b	0,37
X61	46745420	764750	b	51878751	1103372	a	0,00
X62	112391203	5883943	a	89965180	8475520	b	0,03
X63	80050	18341	b	30860823	1934228	a	0,00
X64	42636	10372	b	11931806	834204	a	0,00
X67	4237732	332132	a	1287606	124583	b	0,00
X68	6125356	417747	a	1819024	243120	b	0,00
X69	2494481121	33028896	a	619911717	37327494	b	0,00
X70	34824074	3261095	a	13211928	1831510	b	0,00
X71	6607227	532092	a	1834008	200997	b	0,00
X72	483904733	6411282	a	91938832	7817513	b	0,00
X73	352736972	4331740	a	81501225	5392237	b	0,00
X74	2556218	215625	a	562388	67089	b	0,00
X75	8346	7100	b	1522681	949839	a	0,12
X76	8960213	624779	a	753638	91060	b	0,00
X77	0	0	b	5728109	549437	a	0,00
X78	24464664	2751958	b	26727360	3408994	a	0,61
X79	238958	163636	a	42642	21724	b	0,24
X81	10882459	6187829	a	3661803	2693935	b	0,29
X82	11999849	5906509	a	3901463	2771242	b	0,22
X83	2100	767	b	4453	975	a	0,06
X84	28612466	6873755	b	1121620887	52063105	a	0,00
X85	9302395	2122828	b	9703491	2503551	a	0,90
X86	117481	25836	b	86261589	4357994	a	0,00
X87	25642843	1327948	a	1198726	180751	b	0,00
X88	15703	5464	b	12587536	1038202	a	0,00
X90	6650140	2358773	a	2838758	412880	b	0,12
X92	118760784	1862910	b	120854948	1710299	a	0,41
X95	10148785	815885	a	302306	32786	b	0,00
X96	260451	156280	b	45841982	16616442	a	0,01
X98	3308297	858558	a	72582	9197	b	0,00
X99	17164	4701	b	726566	516784	a	0,18
X100	129568484	16354034	a	102129334	13363753	b	0,20
X101	263419234	3100376	b	281036986	4175082	a	0,00
X102	9922	3561	a	9131	1672	b	0,84
X104	47898	13508	b	17413476	1530838	a	0,00
X105	92982254	1138559	b	100308188	1609220	a	0,00
X106	13494	9425	b	5022390	3358259	a	0,14
X107	241594	76555	a	146803	23691	b	0,24

X109	5812	2256	a	2235	1498	b	0,19
X111	1297823	462999	b	1764642	620430	a	0,55
X112	267017	118742	a	266442	128018	b	1,00
X114	307612	143401	a	57393	31777	b	0,09
X115	13761	9906	b	11767950	1405772	a	0,00
X118	15641928	2177608	a	147870	21712	b	0,00
X119	1243499	261510	a	35856	4289	b	0,00
X120	97447039	1153509	a	4917039	778874	b	0,00
X121	403521	44873	b	493579	50814	a	0,19
X122	0	0	b	130096	31825	a	0,00
X124	183028	47628	a	23486	5758	b	0,00
X125	9911394	2347575	a	3499	1363	b	0,00
X126	39330913	10351044	b	52988778	7275350	a	0,28
X127	423177	337264	a	218246	56602	b	0,55
X128	232226	38627	b	4896285	437921	a	0,00
X129	195171	87374	b	243763	112966	a	0,73
X131	17329773	5094620	a	0	0	b	0,00
X132	25984360	8485759	a	0	0	b	0,00
X133	1020413	519061	a	10471	3718	b	0,06
X135	42331365	13554766	a	52638	6899	b	0,00
X138	1346057	418882	b	4659022	1256461	a	0,02
X140	3785754	1344325	b	4199066	657567	a	0,78
X141	421064	57308	b	854801	812412	a	0,60
X143	766040384	46238012	a	0	0	b	0,00
X145	9697	2342	a	0	0	b	0,00
X147	46132652	3696857	a	0	0	b	0,00
X151	187081	70501	a	150691	27252	b	0,63
X152	1469	910	b	457984	373127	a	0,23
X153	11020674	4131279	a	0	0	b	0,01
X155	118397	30585	b	110973615	6052666	a	0,00
X157	7250501	715902	a	350272	51354	b	0,00
X158	90863384	8083780	a	247933	143865	b	0,00
X159	46840	13389	a	11858	1891	b	0,01
X160	4948914	984532	a	0	0	b	0,00
X161	2308280	451276	a	43603	8194	b	0,00
X164	182773	15485	b	223247	22671	a	0,15
X167	23318942	7038537	a	600238	186792	b	0,00
X171	700189	232123	b	835112	243656	a	0,69
X172	5197	1518	b	11993	2954	a	0,05
X180	1177016	531939	a	16742	6473	b	0,03
X182	62746877	6464652	a	265142	87025	b	0,00

X183	3263432	440942	a	0	0	b	0,00
X185	1465729	240963	a	1547	1262	b	0,00
X190	5920752	799467	a	0	0	b	0,00
X192	2906034228	164326192	a	0	0	b	0,00
X197	245158117	14597564	a	0	0	b	0,00
X198	100061	43115	b	2996163	921459	a	0,00
X201	117045	15112	a	25570	5725	b	0,00
X202	104002	30581	b	70223038	4598196	a	0,00
X209	1196273	289138	a	618666	213441	b	0,11
X211	11838973	1907251	a	2953	980	b	0,00
X212	38684409	8279433	a	25401	7293	b	0,00
X216	1022529	374247	a	12625	5072	b	0,01
X217	6279996	415946	a	688498	110013	b	0,00
X218	31275	6402	a	0	0	b	0,00
X219	5417	2101	b	1020910	540379	a	0,07
X220	304199	77146	a	4933	1235	b	0,00
X221	13917	2966	a	0	0	b	0,00
X223	387112	363076	a	1862	1615	b	0,29
X228	5078788	3659086	a	1519784	1337060	b	0,36
X229	173628	20265	a	98686	8667	b	0,00
X230	9573211	2542982	a	4793556	1434852	b	0,11
X232	5888	2264	a	0	0	b	0,01
X237	203911913	19854660	a	1332122	445228	b	0,00
X240	12614824	1557886	a	31667	10044	b	0,00
X241	1842923	894508	a	153521	92078	b	0,07
X242	25400	4338	a	7471	1775	b	0,00
X243	5227014	1589006	a	802131	489772	b	0,01
X244	58463	16787	b	44992771	2978641	a	0,00
X246	32701	6082	a	0	0	b	0,00
X248	4368508	317897	a	849959	100214	b	0,00
X249	1183045037	15600727	a	216590289	17157645	b	0,00
X250	5361240	462072	a	843223	118857	b	0,00
X252	87041079	4742416	a	3903375	539461	b	0,00
X253	121485390	1693560	a	6853746	993168	b	0,00
X254	65705460	1212772	b	66961253	1045013	a	0,44
X259	3274222	968592	a	5988	2795	b	0,00
X260	1685713	667789	a	4609	1447	b	0,01
X261	28866015	3062773	a	11597	4262	b	0,00
X263	48189321	6239274	a	25562	12199	b	0,00
X264	7623	6343	b	433861	314857	a	0,18
X265	546933	263017	a	689	396	b	0,04

X266	30472527	5348219	a	0	0	b	0,00
X267	20430369	4038424	a	192999	29420	b	0,00
X269	10889470	1713680	a	24394	4644	b	0,00
X274	13884196	3772053	a	10630597	1361313	b	0,42
X276	3082954	279262	b	3098011	381088	a	0,97
X279	148178	21921	a	6535	2305	b	0,00
X281	2036171	309743	a	22558	7528	b	0,00
X284	3425122	1053897	b	582197522	25784593	a	0,00
X285	530758	153079	b	4794575	508384	a	0,00
X287	4802149	505291	b	5639052	602671	a	0,29
X289	30542	8362	b	6877956	530086	a	0,00
X291	15078523	3290351	a	35688	7078	b	0,00
X296	78550456	1757034	a	2464752	377852	b	0,00
X298	57073349	5605465	a	2138058	322849	b	0,00
X301	72923192	9422410	a	69930	46402	b	0,00
X310	3336953	740652	a	25814	3887	b	0,00
X313	918826	226077	a	616627	305841	b	0,43
X315	28995800	3727495	a	46618	5240	b	0,00
X316	84406867	15592627	a	0	0	b	0,00
X319	20334	5379	a	0	0	b	0,00
X321	322597	64337	b	19299791	1871515	a	0,00
X330	47150	10972	a	12342	3342	b	0,00
X332	16584983	4694944	a	1121	558	b	0,00
X335	1007266	160606	a	73608	14031	b	0,00
X337	16762165	3064808	a	6904	2568	b	0,00
X339	260773	43408	b	182779384	9089373	a	0,00
X342	3211186	1420465	a	5958	2495	b	0,03
X343	448370	105273	a	38044	5103	b	0,00
X346	6533178	1260415	a	0	0	b	0,00
X347	54439254	13503284	a	0	0	b	0,00
X350	301911763	28798290	a	2179600	745361	b	0,00
X351	257122	125701	a	4363	4012	b	0,05
X354	548215184	85891846	a	0	0	b	0,00
X356	26499732	3011533	a	89817	34038	b	0,00
X357	17076465	993135	a	738145	103577	b	0,00
X358	52528195	10524441	a	0	0	b	0,00
X359	826626	271332	a	4970	1106	b	0,00
X360	11106311	1820458	b	11513024	1938544	a	0,88
X364	38235971	3735815	a	69483	31429	b	0,00
X366	54197	16843	a	0	0	b	0,00
X368	1003137	670533	a	2822	984	b	0,14

X374	2120839	1119905	a	0	0	b	0,06
X378	4304091	1801994	a	15457	13603	b	0,02
X379	32020534	12619769	a	0	0	b	0,01
X381	36189	20933	a	27947	10855	b	0,73
X384	7650802	2800979	a	1338	1156	b	0,01
X390	16989066	2925303	a	1117	628	b	0,00
X391	35588498	7800093	a	16450751	4245269	b	0,04
X392	4937529	1039817	a	0	0	b	0,00
X393	23256140	5997885	a	0	0	b	0,00
X397	763983	335165	a	0	0	b	0,03
X399	657191079	153780765	a	0	0	b	0,00
X401	96458800	24444150	a	0	0	b	0,00
X404	10758167	1237083	a	541158	82045	b	0,00
X415	16366014	4169637	a	0	0	b	0,00
X420	3281236	1493800	a	7753	2755	b	0,03
X422	238893	58856	b	1228459	378432	a	0,01
X423	11987184	1567868	b	13023597	1787703	a	0,66
X424	9879	6469	b	515832	131569	a	0,00
X426	107783	22977	b	22305535	1794553	a	0,00
X427	533627	258455	a	0	0	b	0,04
X429	0	0	b	443118	56559	a	0,00
X430	47520	7477	b	54755	7886	a	0,51
X433	13824429	2584890	a	35431	8311	b	0,00
X436	41311	9064	a	0	0	b	0,00
X437	70817	9874	b	1677340	748636	a	0,04
X439	119792	27952	a	767	443	b	0,00
X441	9832636	668180	a	466982	81480	b	0,00
X445	139901	68415	a	0	0	b	0,05
X458	3283103	1283773	a	8646	4118	b	0,01
X460	0	0	b	13742188	2260863	a	0,00
X461	15808	5476	b	4087408	1446843	a	0,01
X462	11500989	2122483	a	313754	36252	b	0,00
X463	0	0	b	259979	113885	a	0,03
X464	1949252	805431	a	0	0	b	0,02
X465	1881743	555851	a	6371	3488	b	0,00
X467	78856	15125	b	3602783	824939	a	0,00
X470	28035	7950	b	415162	238505	a	0,11
X471	95194	21303	b	199348	49084	a	0,06
X474	325355	76666	b	9812046	954495	a	0,00
X480	6599174	688913	b	11168836	504972	a	0,00
X485	5245060	1092831	a	795	553	b	0,00

X489	676054	82812	b	6938326	2204862	a	0,01
X490	6158504	1249832	a	0	0	b	0,00
X491	423516	182271	a	28599	9028	b	0,03
X495	1844	991	b	357201	283319	a	0,21
X496	632668	323137	a	11804	3480	b	0,06
X499	9013664	2200231	a	0	0	b	0,00
X500	1392	852	b	1404409	758872	a	0,07
X502	50726	10997	b	84466	30953	a	0,31
X504	45748	10037	a	21889	7910	b	0,07
X505	103962	31179	a	0	0	b	0,00
X509	41536	9711	b	134663	24440	a	0,00
X510	19113524	2478590	a	0	0	b	0,00
X511	1442619	293629	a	65273	35926	b	0,00
X513	7147478	1879266	a	0	0	b	0,00
X528	12096	6254	a	3534	2136	b	0,20
X536	484684	116438	a	0	0	b	0,00
X538	3442092	1332200	a	126707	27499	b	0,02
X540	8512	2745	a	612	432	b	0,01
X542	163474	38655	a	9711	3381	b	0,00
X543	99733	21783	a	5716	2301	b	0,00
X545	203419	38769	a	4224	1375	b	0,00
X546	22787	6206	a	5371	1375	b	0,01
X547	4751654	1195677	a	0	0	b	0,00
X552	4471888	1093280	a	1291	725	b	0,00
X567	202	115	b	7614	1304	a	0,00
X568	11477085	2075080	a	0	0	b	0,00
X569	20239892	4627230	a	0	0	b	0,00
X571	7973128	1578621	a	0	0	b	0,00
X573	443199	50700	b	1994724	559262	a	0,01
X576	34936	6090	b	119841	27130	a	0,00
X579	0	0	b	929420	354415	a	0,01
X586	9215929	1117659	a	0	0	b	0,00
X600	151399	44029	b	8284343	1134217	a	0,00
X601	724607	724358	a	7899	3111	b	0,33
X605	81482	25523	b	14558766	1935137	a	0,00
X606	351412	69752	a	7417	2037	b	0,00
X613	70467043	10828752	a	0	0	b	0,00
X614	69196	13497	b	565259	399673	a	0,22
X615	20837286	2430702	a	7926	6458	b	0,00
X617	8166122	2414977	a	0	0	b	0,00
X622	396772	108613	a	33556	10042	b	0,00

X632	34044860	5033974	a	0	0	b	0,00
X638	3416863	901475	a	0	0	b	0,00
X641	37508601	12247229	a	0	0	b	0,00
X642	10250868	2307260	a	0	0	b	0,00
X648	302852	75680	a	0	0	b	0,00
X650	1054	448	a	897	456	b	0,81
X653	27752823	2567082	a	0	0	b	0,00
X663	16646825	3857173	a	0	0	b	0,00
X665	3194499	509469	a	83543	16247	b	0,00
X666	0	0	b	22879	6825	a	0,00
X676	209466	194683	a	4789	1101	b	0,30
X682	7056	2791	b	2507745	382342	a	0,00
X690	196433	72178	a	0	0	b	0,01
X711	144335667	11700517	a	180097	66172	b	0,00
X714	24001081	2533036	a	21709	9063	b	0,00
X717	67996	21027	b	73058	29127	a	0,89
X720	3401	1432	b	5863	3714	a	0,54
X722	143309	39744	a	0	0	b	0,00
X725	3823122	1750746	a	198199	119484	b	0,04
X728	610894	324763	a	21986	14238	b	0,08
X729	1931697	971288	a	286044	153828	b	0,10
X731	84024	31766	a	53081	18349	b	0,40
X732	32547	19764	a	1062	692	b	0,12
X736	4344677	536202	a	3790728	570568	b	0,48
X747	17040994	5692466	a	186292	57197	b	0,00
X750	45175752	3625880	a	5266	1856	b	0,00
X751	1064575	957655	a	8718	2030	b	0,27
X752	1034	719	b	50509	15814	a	0,00
X753	8019838	1067120	a	0	0	b	0,00
X755	7068	2260	b	557652	283509	a	0,06
X756	64865	26672	a	0	0	b	0,02
X757	806	581	b	37938	22533	a	0,10
X764	18096805	1317397	a	22892	9126	b	0,00
X765	200690	56970	a	1652	733	b	0,00
X766	101256	32830	a	0	0	b	0,00
X767	28072574	7306007	a	484828	269359	b	0,00
X768	4843308	1676342	a	69551	34910	b	0,01
X769	14981625	4994102	a	615286	251142	b	0,01
X770	2543649	1033524	a	91707	36304	b	0,02
X771	1272765	1152767	a	34278	6348	b	0,29
X774	5417778	734564	b	6298645	956998	a	0,47

X775	0	0	b	2345820	673323	a	0,00
X776	7942584	812197	a	2033	1279	b	0,00
X777	920630	258823	a	0	0	b	0,00
X779	3601399	513928	a	37283	12825	b	0,00
X780	1437444	470651	a	0	0	b	0,00
X781	2047377	596415	a	830	583	b	0,00
X782	58222	23735	b	3177663	1411577	a	0,03
X783	6581	2687	b	656865	257902	a	0,01
X784	635	442	b	52376	17889	a	0,01
X785	10640	2369	b	2566550	1904385	a	0,18
X787	30623250	3672276	a	0	0	b	0,00
X788	3030803	747070	a	0	0	b	0,00
X792	3364349	916952	b	3864832	602765	a	0,65
X793	0	0	b	38735	21052	a	0,07
X796	10922116	1753771	a	41691	21118	b	0,00
X797	883113	418948	a	9442	5186	b	0,04
X798	84884512	4860135	a	44623	24509	b	0,00
X799	188079	65541	a	0	0	b	0,01
X800	16631234	1395833	a	6899	3140	b	0,00
X803	931165	282641	a	532	373	b	0,00
X804	1943435	753236	a	2149	478	b	0,01
X805	7094721	1309270	a	91622	34811	b	0,00
X806	379674896	10811158	a	3702026	1145142	b	0,00
X807	102123974	3224349	a	238504	143700	b	0,00
X808	8761771	944742	a	49613	18568	b	0,00
X810	3960628	578772	a	3485133	493707	b	0,53
X811	292630	164224	a	2235	656	b	0,08
X812	9419570	1258050	a	33776	18886	b	0,00
X813	1049211	247246	a	305	212	b	0,00
X814	5417204	1067564	a	17731	13483	b	0,00
X817	6477186	1701593	a	1165	582	b	0,00
X818	23931210	4230406	a	10286	3635	b	0,00
X819	29689079	5020234	a	17841	6248	b	0,00
X820	23050141	4318740	a	8014	2834	b	0,00
X821	11680212	2573027	a	8047	2965	b	0,00
X822	4268668	1165667	a	1300	913	b	0,00
X825	0	0	b	39790	19607	a	0,05
X826	15444686	1177940	a	0	0	b	0,00
X829	860861	324720	a	0	0	b	0,01
X830	1738200	386554	a	0	0	b	0,00
X831	2506809	708361	a	0	0	b	0,00

X835	17225098	7627154	a	0	0	b	0,03
X1000	2818989	381036	a	262385	61870	b	0,00
X1002	2486943	669127	a	245283	48811	b	0,00
X1003	21853189	3386032	a	860211	162233	b	0,00
X1004	1193998	594704	a	16387	1993	b	0,05
X1005	13418387	2507664	a	79239	24764	b	0,00
X1006	381213	58319	b	10052137	1613396	a	0,00
X1007	2187831	1593144	b	71295281	8479105	a	0,00
X1008	7603993	1915017	a	454475	96227	b	0,00
X1010	379423	105280	a	308051	88884	b	0,61
X1012	1575677	221207	a	218049	25079	b	0,00
X1013	1911895	294556	a	779149	120570	b	0,00
X1014	135938998	13426811	a	3416520	714652	b	0,00
X1015	1691753	348577	a	1423053	282801	b	0,55
X1017	6325395	540824	a	418564	172303	b	0,00
X1018	1462055	457130	a	13280	1665	b	0,00
X1019	1495916	218469	a	640560	288713	b	0,02
X1020	12997	4056	b	20304	4452	a	0,23
X1022	185885719	21205844	a	73412422	13665080	b	0,00
X1023	14590301	1631082	a	7850583	986669	b	0,00
X1024	2530204	257596	b	2934549	402350	a	0,40
X1025	7063599	1406645	a	2483975	282348	b	0,00
X1026	924558	142892	a	235229	23411	b	0,00
X1027	924642	202400	a	623490	132746	b	0,22
X1028	313903	48781	a	228908	30990	b	0,15
X1030	704702	176976	a	40420	3586	b	0,00
X1033	122539	24693	b	561230	327859	a	0,19
X1035	4957112	633822	a	366227	97072	b	0,00
X1036	2833043	593740	a	168906	35051	b	0,00
X1037	442422	82622	a	60955	16150	b	0,00
X1039	588355	132841	a	3449	3178	b	0,00
X1040	2754130	497829	a	45120	10518	b	0,00
X1041	284631	58424	a	13488	1760	b	0,00
X1043	4737633	642818	a	18642	2396	b	0,00
X1044	14242688	1675415	a	26258	6430	b	0,00
X1045	40408264	6132446	a	2543893	721346	b	0,00
X1046	2871532	386750	a	69133	7983	b	0,00
X1047	1740398	323413	a	164305	54342	b	0,00
X1048	2959662	213804	a	70685	7196	b	0,00
X1049	8883661	887950	a	761627	393953	b	0,00
X1051	3388033	557849	a	351422	40128	b	0,00

X1052	15453115	2269854	a	1099979	183029	b	0,00
X1053	25706145	882926	b	29192143	512056	a	0,00
X1056	45684	7138	a	29545	4522	b	0,06
X1057	14386	8135	a	2027	1798	b	0,14
X1058	257249	39431	b	866934	177970	a	0,00
X1059	900807	356822	a	15082	4520	b	0,02
X1061	0	0	b	7492	2802	a	0,01
X1062	120612729	7313543	a	3952817	802264	b	0,00
X1066	1927274	368251	a	24891	2789	b	0,00
X1067	32618	2761	a	26044	3440	b	0,14
X1068	3147950	396714	a	43158	5227	b	0,00
X1069	85137	11166	a	46445	4167	b	0,00
X1070	242991	80602	a	54599	6063	b	0,02
X1071	14604225	1577537	a	2295	885	b	0,00
X1072	330783	75029	a	5168	1041	b	0,00
X1074	3185	967	b	46629	7696	a	0,00
X1075	110524	20951	a	49031	30062	b	0,10
X1076	1868717	244787	a	109899	36518	b	0,00
X1077	644	461	b	222790	60568	a	0,00
X1078	101475	41179	a	47184	13875	b	0,22
X1079	1050604	317213	a	3766	675	b	0,00
X1080	2426861	737314	a	59646	5987	b	0,00
X1081	105859	15165	b	297138	98755	a	0,06
X1082	229258	93287	a	103602	28706	b	0,20
X1083	1210825	319231	a	62640	13351	b	0,00
X1084	317457	67824	a	14074	6387	b	0,00
X1085	2963230	364115	a	13297	3585	b	0,00
X1086	44134163	2052298	a	280960	43462	b	0,00
X1087	8570817	1149011	a	111999	19902	b	0,00
X1088	94746	10774	a	58736	7519	b	0,01
X1089	6800470	946912	a	478473	101225	b	0,00
X1091	80485	11737	a	24050	2376	b	0,00
X1092	57603513	4746921	a	61138	17242	b	0,00
X1093	623847	173132	a	6150	2462	b	0,00
X1095	58776	7337	b	334996	140161	a	0,05
X1096	641909	74319	a	284299	207713	b	0,11
X1097	1767550	319836	a	1362599	279255	b	0,34
X1101	51661	7184	b	426073	100329	a	0,00
X1103	3473287	1279118	a	657476	153969	b	0,03
X1106	1268708	204372	a	16300	6722	b	0,00
X1107	25786270	4856298	a	219106	29871	b	0,00

X1108	6122537	804092	a	260722	121830	b	0,00
X1110	616427	209136	a	10265	5150	b	0,01
X1113	73703	14698	a	5937	1336	b	0,00
X1114	2167128	245010	a	381985	62662	b	0,00
X1115	465057	77993	a	11298	2071	b	0,00
X1117	82417	13157	b	94811	25401	a	0,67
X1118	62820	15900	b	187493	63450	a	0,06
X1119	2598153	425964	a	9649	3643	b	0,00
X1120	0	0	b	7926	2737	a	0,01
X1121	44151	23833	a	17197	5811	b	0,28
X1122	570929	70937	b	5798849	1030164	a	0,00
X1123	15917947	1458394	a	81853	32032	b	0,00
X1124	40752822	1798796	a	433835	92881	b	0,00
X1126	29419962	3331474	a	19520	7848	b	0,00
X1128	209385	32050	a	14653	5361	b	0,00
X1131	6951729	1180929	a	69184	8963	b	0,00
X1132	346165	41572	a	67318	6622	b	0,00
X1134	18651789	2446924	a	1681362	364119	b	0,00
X1136	1370608	170057	a	18108	1832	b	0,00
X1137	1716674	260861	a	234023	42596	b	0,00
X1142	1200005	201313	a	2076	765	b	0,00
X1143	1033280	329363	b	3584736	972946	a	0,02
X1145	156350	40768	a	6557	1085	b	0,00
X1146	417735	104048	a	16193	11573	b	0,00
X1147	16377	14517	a	4106	741	b	0,40
X1148	696058	220995	a	67834	14887	b	0,01
X1149	67047464	2014487	a	24943089	2272054	b	0,00
X1150	7068633	362685	a	2273383	275253	b	0,00
X1152	7898508	387773	a	2452971	299017	b	0,00
X1153	60867	11681	a	19478	2901	b	0,00
X1156	2145149	502935	a	53631	12057	b	0,00
X1157	1029932	157503	a	75766	16464	b	0,00
X1160	9806	5307	b	459947	291920	a	0,13
X1161	1967184	148474	a	85340	16959	b	0,00
X1162	3748644	875784	a	16324	5162	b	0,00
X1166	1940650	358184	a	80548	31346	b	0,00
X1167	12789	1727	a	7870	1127	b	0,02
X1169	2021979	785504	a	287	200	b	0,01
X1170	42276010	5775802	a	38542876	4098950	b	0,60
X1171	81963	26017	b	392453	121959	a	0,02
X1175	172414	43258	b	42818789	1942143	a	0,00

X1176	10634677	1126690	a	9689850	1524208	b	0,62
X1177	9613724	1992589	a	7375453	1537989	b	0,38
X1181	736977	139110	a	33476	4621	b	0,00
X1182	2240841	923988	a	9628	7354	b	0,02
X1183	2496331	241900	a	40540	17541	b	0,00
X1184	57609	10158	b	76705	8640	a	0,16
X1185	17805630	2146138	a	80529	10021	b	0,00
X1187	1797865	254999	a	18300	4194	b	0,00
X1188	1549330	376384	a	1215	755	b	0,00
X1191	9313220	502084	b	10455017	424222	a	0,09
X1193	7656874	1174235	a	8352	3214	b	0,00
X1194	1469183	681462	a	46071	20831	b	0,04
X1197	4912277	521910	a	5493	1996	b	0,00
X1201	1049474	389513	a	0	0	b	0,01
X1203	2283153	294274	a	44976	9206	b	0,00
X1208	4461208	889429	a	295304	83031	b	0,00
X1209	226598	47509	a	15826	6145	b	0,00
X1210	1768146	491185	a	578426	115774	b	0,02
X1211	2295756	912799	a	2402	1166	b	0,01
X1212	10452739	1624993	a	0	0	b	0,00
X1213	52279613	7388878	a	4005738	1098204	b	0,00
X1214	875924	288341	a	181597	62689	b	0,02
X1217	123457	52386	b	263263	91534	a	0,19
X1218	675	383	b	10348	3014	a	0,00
X1220	48418	27336	b	55643	17425	a	0,82
X1223	2410704	719776	a	3761	1915	b	0,00
X1228	23153818	5236455	a	0	0	b	0,00
X1231	625490	107605	b	818062	291131	a	0,54
X1233	708428	132755	a	97268	62732	b	0,00
X1238	2897174	522502	a	1872288	471088	b	0,15
X1239	7865169	1023084	a	631916	229496	b	0,00
X1241	2057870	564657	a	0	0	b	0,00
X1242	1955945	344228	a	3134	1284	b	0,00
X1243	2005184	266394	a	142494	57671	b	0,00
X1246	2529068	602478	a	0	0	b	0,00
X1252	10573	2188	b	28645	9451	a	0,07
X1255	11909	9204	a	2092	606	b	0,29
X1260	550691	228152	b	1375104	706107	a	0,27
X1261	20447006	1569357	a	5314	2659	b	0,00
X1266	9244414	1640250	a	0	0	b	0,00
X1274	726285	250935	b	12412502	3098716	a	0,00

X1277	101884	23356	a	76497	19531	b	0,41
X1278	32476	11878	b	1028441	432205	a	0,02
X1279	7910388	2058602	a	0	0	b	0,00
X1281	87695	35643	a	40351	12207	b	0,21
X1282	1034851	189099	a	70248	32074	b	0,00
X1283	0	0	b	5478	1891	a	0,01
X1284	75019	19973	a	3492	2450	b	0,00
X1286	470816	132543	a	0	0	b	0,00
X1289	719273	219875	b	1978867	352260	a	0,00
X1290	2856671	879343	a	16649	3370	b	0,00
X1291	31973302	2412517	a	12692389	2422861	b	0,00
X1292	3561161	629882	a	735081	346882	b	0,00
X1295	1020742	167509	a	237631	109473	b	0,00
X1296	8812819	940938	a	6075519	1226683	b	0,08
X1297	2392	598	b	12632	6261	a	0,11
X1299	824458	410005	a	6671	930	b	0,05
X1300	163462173	13054452	a	1098452	603615	b	0,00
X1302	59832022	15475636	a	0	0	b	0,00
X1303	518543	130932	a	38027	10571	b	0,00
X1304	1343401	265118	b	3156226	681292	a	0,02
X1305	31924262	2791753	a	42540	19879	b	0,00
X1308	22964642	5299789	a	0	0	b	0,00
X1309	3032112	454763	a	1438	716	b	0,00
X1311	724420	124447	b	897585	266675	a	0,56
X1317	2880301	744745	a	1232	347	b	0,00
X1319	47726	17704	b	47817	17790	a	1,00
X1321	987706	184108	a	117955	42655	b	0,00
X1327	235908	68550	a	8734	3371	b	0,00
X1328	111599	47210	a	89076	27913	b	0,68
X1329	37256572	14456994	b	56729116	16192478	a	0,37
X1330	706964	232469	a	57722	12442	b	0,01
X1334	577181	200839	a	0	0	b	0,01
X1335	3707677	1446071	b	5459413	1680718	a	0,43
X1338	3744456	524943	a	2448135	452567	b	0,07
X1339	2026915	1878596	a	188705	52517	b	0,33
X1340	58374	15508	b	138243	58337	a	0,19
X1343	99624355	3778172	a	1262464	253819	b	0,00
X1345	415800	81866	b	1104577	583547	a	0,25
X1350	1003046	177777	a	33415	24728	b	0,00
X1351	10560806	615623	a	102799	23085	b	0,00
X1354	1654166	201609	a	77799	18250	b	0,00

X1355	396984	137952	b	8180433	1950813	a	0,00
X1358	106100285	9511431	a	35481	9881	b	0,00
X1359	973611063	71175853	a	4844769	1433261	b	0,00
X1360	205114	56395	b	919918	543803	a	0,20
X1361	459698	87709	b	774377	287556	a	0,30
X1363	11249730	1584652	a	0	0	b	0,00
X1366	0	0	b	1239544	375495	a	0,00
X1370	10665	2194	a	0	0	b	0,00
X1372	7726913	662515	a	592900	214050	b	0,00
X1374	8008	3548	b	34690	17147	a	0,13
X1375	5034	1566	a	1538	594	b	0,04
X1376	4207113	637841	a	141089	57744	b	0,00
X1377	106364	61289	a	16091	10943	b	0,15
X1380	4726545	694250	a	0	0	b	0,00
X1385	7653029	280735	b	8395283	200967	a	0,04
X1393	26496840	3233039	a	2896	1627	b	0,00
X1394	2340741	233012	a	17182	5921	b	0,00
X1400	4140110	519939	a	50768	17569	b	0,00
X1403	139276	70782	a	90643	38450	b	0,55
X1405	939530	303526	a	6164	2257	b	0,00
X1407	215262	41595	a	1062	717	b	0,00
X1408	30860937	2929446	a	182396	85154	b	0,00
X1409	147409986	10390910	a	106390	32860	b	0,00
X1411	331521	97228	a	297061	109652	b	0,81
X1412	1082767	598940	a	253526	88407	b	0,18
X1415	135527	37012	b	5280780	3451457	a	0,14
X1416	20428	4776	b	1259219	1080893	a	0,26
X1417	984638	250202	a	163777	41922	b	0,00
X1422	2761794	1249238	a	233406	65728	b	0,05
X1423	15387	10163	b	1678492	508485	a	0,00
X1424	157541	65668	b	373347	249160	a	0,41
X1425	6230459	676391	a	398795	86203	b	0,00
X1426	10465380	2525297	a	0	0	b	0,00
X1427	1714183	430481	a	326307	96113	b	0,00
X1428	2958	1468	b	3559	1099	a	0,74
X1431	0	0	b	143657	47692	a	0,00
X1433	966	493	b	162752	68055	a	0,02
X1434	52782603	2875319	a	1018369	176709	b	0,00
X1435	725	499	b	139272	58985	a	0,02
X1437	39442260	4441703	a	1661652	396755	b	0,00
X1438	7136	3211	a	1631	416	b	0,09

X1439	226232	134647	a	190202	88550	b	0,82
X1440	5345329	419806	a	109423	18779	b	0,00
X1442	3548266	395546	a	217502	46620	b	0,00
X1443	7110	2314	b	108344	49341	a	0,04
X1445	9634	6542	a	2487	1013	b	0,28
X1455	2364585	477263	a	368357	139981	b	0,00
X1457	219361	48831	a	29176	12974	b	0,00
X1462	418495	46486	a	13041	4853	b	0,00
X1467	18709	4767	a	0	0	b	0,00
X1469	7129548	1298889	a	59747	25824	b	0,00
X1470	6533354	1522784	a	0	0	b	0,00
X1477	1247719	1039119	a	132453	49997	b	0,29
X1478	890402	196395	b	3320555	1548403	a	0,12
X1479	318067	102764	a	45499	12260	b	0,01
X1480	64990	50248	a	5672	2302	b	0,24
X1484	9756435	1210196	a	0	0	b	0,00
X1486	7639	3565	a	6056	3691	b	0,76
X1487	3637979	881243	a	747846	275392	b	0,00
X1489	534565	161012	a	100000	45863	b	0,01
X1491	765894	337933	b	2801959	881463	a	0,04
X1495	5567586	966673	a	1611	1067	b	0,00
X1496	12176663	2189177	a	489441	125210	b	0,00
X1497	45221	27757	b	62170	32055	a	0,69
X1498	958670	213801	b	1258584	351977	a	0,47
X1499	9776129	2089584	a	1910341	688150	b	0,00
X1500	1136302	267604	a	32367	10388	b	0,00
X1501	104744	31847	b	211604	67364	a	0,16
X1503	8463	2473	a	7161	3136	b	0,75
X1509	9148099	792297	a	71532	18221	b	0,00
X1514	34012	8178	b	208861	99854	a	0,09
X1524	24027708	2307201	a	59748	23198	b	0,00
X1526	210186100	26836127	a	487574	118803	b	0,00
X1527	314176157	33807016	a	2962961	880453	b	0,00
X1529	19974401	2452655	a	503101	133783	b	0,00
X1530	3581451	381270	a	2596	1502	b	0,00
X1534	48437	21189	b	70315	34923	a	0,59
X1535	36076180	4386275	a	15366	6661	b	0,00
X1536	51175141	5942771	a	229325	84104	b	0,00
X1537	1219746	158012	a	20953	8138	b	0,00
X1540	11868000	1040228	a	27648	8498	b	0,00
X1541	6972096	513037	a	174062	42138	b	0,00

X1543	8829507	878928	a	9536	3320	b	0,00
X1544	2629825	1557873	a	215168	68324	b	0,13
X1547	9568084	797280	a	72567	17706	b	0,00
X1548	2093877	197531	a	11874	3853	b	0,00
X1550	221638951	13562855	a	3873596	1048421	b	0,00
X1551	14082223	3438230	a	82969	25365	b	0,00
X1554	737895	216266	a	20537	7407	b	0,00
X1555	131273	34157	b	1424207	322131	a	0,00
X1558	93927643	3516850	a	26618567	3777449	b	0,00
X1560	2279215	672923	a	2922	1267	b	0,00
X1561	59875776	8736280	a	430113	240813	b	0,00
X1562	141664	31286	a	17999	5658	b	0,00
X1563	5524434	1946537	b	62482683	13510922	a	0,00
X1564	3174	1728	b	97519	43170	a	0,03
X1565	28009173	1673818	a	5381415	997010	b	0,00
X1567	8795124	1542175	a	26852	19578	b	0,00
X1568	30524	22608	b	2430790	670782	a	0,00
X1569	24499767	1116096	a	6499499	1003626	b	0,00
X1570	15446675	1422817	a	7061	2081	b	0,00
X1573	9072036	603667	a	86502	18126	b	0,00
X1576	5990	2960	b	786893	418659	a	0,07
X1578	2974095	248565	a	136466	43382	b	0,00
X1583	16597	5551	b	62199	39745	a	0,26
X1585	871301	162659	a	6371	2211	b	0,00
X1590	14041926	2745864	a	65599	22158	b	0,00
X1592	19420	5435	b	28980	8214	a	0,34
X1597	23270	5377	a	2024	1139	b	0,00
X1601	124622	20571	a	2675	1085	b	0,00
X1602	102716	26044	b	192932	85998	a	0,32
X1603	11044786	1971827	a	0	0	b	0,00
X1604	1705673	180454	a	45209	8099	b	0,00
X1605	186778	42711	a	32475	4453	b	0,00
X1606	2569156197	102201555	a	85193820	13551760	b	0,00
X1607	2111111	346015	a	29122	9409	b	0,00
X1610	90947956	3765104	a	380607	125446	b	0,00
X1613	453508161	17884386	a	12478296	2332161	b	0,00
X1615	40978388	2162188	a	190802	39640	b	0,00
X1621	51893716	2232592	a	170955	57216	b	0,00
X1622	18909193	1667019	a	26063	11606	b	0,00
X1625	800305	361846	a	21688	9249	b	0,04
X1627	4794877	343292	a	9818	4179	b	0,00

X1631	1084598	146519	b	19164291	924012	a	0,00
X1633	787802	266625	a	82653	23776	b	0,01
X1635	2462162	221831	a	53179	17821	b	0,00
X1636	1931819	288088	a	100787	35706	b	0,00
X1638	251838	117699	a	77892	54214	b	0,18
X1639	3819964	628972	a	4728	2190	b	0,00
X1640	171073	51381	a	4038	2473	b	0,00
X1641	20609	6492	a	6936	3704	b	0,07
X1647	5209062	722665	a	5332	2574	b	0,00
X1648	4568879	485911	a	45432	16734	b	0,00
X1649	5590501	1256218	a	0	0	b	0,00
X1653	3408032	407461	a	19230	7132	b	0,00
X1656	1795257	715570	a	2866	2144	b	0,02
X1659	369408221	26615601	a	1123391	304347	b	0,00
X1660	48052397	4236907	a	140395	44648	b	0,00
X1661	40240005	3360289	a	293601	128161	b	0,00
X1662	5904808	1334733	a	69633	25502	b	0,00
X1663	811759	118863	a	13852	4337	b	0,00
X1665	20280	4086	a	0	0	b	0,00
X1668	62593254	4608134	a	96611	25287	b	0,00
X1669	6122676	861146	a	2347	1310	b	0,00
X1670	4789135	531741	a	29543	20359	b	0,00
X1671	524100	209063	a	8424	3903	b	0,02
X1675	10841	7029	a	409	296	b	0,14
X1677	34349019	6898582	a	22063480	6835550	b	0,21
X1678	23781338	1021840	a	173892	44332	b	0,00
X1683	46066335	4015560	a	49264	14608	b	0,00
X1684	10569815	1870270	a	0	0	b	0,00
X1685	4503012	1010289	a	3321784	1063675	b	0,42
X1686	82575200	11053998	a	17534315	2673311	b	0,00
X1687	13343047	1349706	a	66752	43764	b	0,00
X1688	30357	5369	a	0	0	b	0,00
X1690	32321175	2789390	a	37350	10406	b	0,00
X1692	88982	25333	a	63204	23631	b	0,46
X1693	7415936	1758302	a	0	0	b	0,00
X1694	4847838	358288	a	1109025	393353	b	0,00
X1697	2533775	1209316	a	25652	10051	b	0,04
X1700	2095563	326604	a	231303	114307	b	0,00
X1701	10115	6400	a	3091	1587	b	0,29
X1707	904069	191211	a	378959	107862	b	0,02
X1708	8299	2439	b	18397390	1661499	a	0,00

X1711	50526	10788	a	4251	1826	b	0,00
X1713	3079513	317303	a	49823	12322	b	0,00
X1715	8003464	1675465	b	24230703	2638315	a	0,00
X1716	2623535	617256	a	51450	14006	b	0,00
X1717	6786	5776	a	5336	4223	b	0,84
X1718	134032	38138	a	13181	9173	b	0,00
X1722	2908234	520620	b	6549092	841934	a	0,00
X1726	6207	2105	b	12599	5613	a	0,29
X1729	573488	81633	a	2474	715	b	0,00
X1736	19132	5869	a	9732	5495	b	0,25
X1738	5032042	745716	a	316077	71149	b	0,00
X1741	16859662	2172672	a	0	0	b	0,00
X1742	20265270	3040983	a	33197	10810	b	0,00
X1743	529466	83283	a	83691	16098	b	0,00
X1747	107368	21512	a	2295	542	b	0,00
X1750	34033	15993	b	135709	49072	a	0,05
X1751	4797165	364766	b	5673436	314168	a	0,07
X1755	10912108	1140264	a	820521	365876	b	0,00
X1761	5104936	805088	a	45758	19256	b	0,00
X1765	2001259	541296	a	0	0	b	0,00
X1766	214833	96973	b	558931	177953	a	0,09
X1768	42314	14613	a	0	0	b	0,01
X1770	205534	73958	a	71143	52600	b	0,14
X1771	84588	14006	a	1797	658	b	0,00
X1774	22863	12683	a	5237	3881	b	0,19
X1777	48016284	3777830	a	579398	109523	b	0,00
X1778	24244	13840	a	7184	3993	b	0,24
X1779	36704	8643	a	7601	1666	b	0,00
X1780	7931900	1124473	a	0	0	b	0,00
X1785	363692	73287	a	1264	633	b	0,00
X1786	6866650	663993	a	78920	15970	b	0,00
X1788	590594	87960	a	4489	2577	b	0,00
X1789	125462	16180	a	25346	12011	b	0,00
X1790	486437	65094	a	147430	58192	b	0,00
X1792	50264	11932	a	17036	8877	b	0,03
X1795	56645	16181	b	145267	47884	a	0,08
X1799	3519	1388	b	3730	3145	a	0,95
X1804	269404	83913	b	647055	369581	a	0,32
X1806	3075018	530126	b	4031467	962970	a	0,39
X1807	4214912	314739	a	55279	14430	b	0,00
X1810	14058659	1975240	a	271768	91947	b	0,00

X1811	9685964	762303	a	269417	49996	b	0,00
X1813	7399	3996	a	337	266	b	0,08
X1814	9838977	2376357	a	0	0	b	0,00
X1816	1763868	147230	a	33073	8245	b	0,00
X1819	3733187	469724	a	55305	17321	b	0,00
X1821	142143	29110	b	224095	55944	a	0,20
X1822	122764695	8958576	a	1577830	382663	b	0,00
X1823	1029720	249913	a	2013	883	b	0,00
X1824	484251	113398	a	3712	2332	b	0,00
X1826	6200752	988298	a	14013	5470	b	0,00
X1833	7602	1640	a	2707	646	b	0,01
X1837	165834	35243	a	52292	24269	b	0,01
X1839	246261	31527	a	16213	7173	b	0,00
X1843	424036	76803	a	1281	610	b	0,00
X1846	6000185	1078444	a	9271	5370	b	0,00
X1847	813988	244029	a	3695	1699	b	0,00
X1851	7644295	772841	a	969043	213245	b	0,00
X1853	50189132	5762301	a	213323	80116	b	0,00
X1854	660071	280238	a	14437	8087	b	0,02
X1856	1375824	180946	a	171649	40483	b	0,00
X1862	3097349	670842	a	0	0	b	0,00
X1866	1284822	536078	a	0	0	b	0,02
X1867	3172986	681567	a	1016	406	b	0,00
X1871	45841	21416	a	1171	1027	b	0,04
X1872	745370	242107	b	1247832	536330	a	0,40
X1875	10780572	1211137	a	23976	8318	b	0,00
X1878	8245404	623675	a	51472	12206	b	0,00
X1881	11149	3995	a	0	0	b	0,01
X1886	5831174	503447	a	261199	79587	b	0,00
X1890	8732	3774	a	563	372	b	0,04
X1894	3032100	272158	a	83240	25202	b	0,00
X1895	265093	44080	a	19607	10684	b	0,00
X1897	0	0	b	14333	4529	a	0,00
X1898	0	0	b	9204	2000	a	0,00
X1900	0	0	b	1738	555	a	0,00
X1901	21797	7540	a	6293	1297	b	0,05
X1902	1338594	404737	a	21383	7313	b	0,00
X1906	276717	76569	a	1072	292	b	0,00
X1918	737003	193033	a	8894	4269	b	0,00
X1921	51526	30617	a	4226	2569	b	0,13
X1922	745583	100602	a	18064	7500	b	0,00

X1923	57730122	5446148	a	25424	16006	b	0,00
X1924	9832444	1362825	a	31109	13668	b	0,00
X1925	16575	2558	b	323701	180185	a	0,09
X1927	11746505	1281648	a	49835	19122	b	0,00
X1930	12388290	2157264	a	9903	3781	b	0,00
X1932	19197	3726	a	0	0	b	0,00
X1933	24565569	4767608	a	278195	141361	b	0,00
X1934	47924717	4659128	a	1240451	324088	b	0,00
X1937	286516	36786	a	25827	6882	b	0,00
X1938	4534679	1053032	a	41647	28621	b	0,00
X1939	10097730	1075352	a	182281	58436	b	0,00
X1943	21749188	2682875	a	32554	12693	b	0,00
X1951	4591811	647987	a	2245	1736	b	0,00
X1953	3524988	977438	a	40451	15544	b	0,00
X1955	59118250	5279821	a	1062723	231312	b	0,00
X1956	7683253	1506755	a	0	0	b	0,00
X1958	22204625	1540324	a	93019	24670	b	0,00
X1959	2107474	1146852	a	1171	1104	b	0,07
X1960	45421	11268	b	480665	89490	a	0,00
X1961	13144046	1216539	a	253442	54414	b	0,00
X1963	3862655	407089	a	18061	5573	b	0,00
X1970	2710106	562748	a	4962	2202	b	0,00
X1974	11281	2980	b	31208	11941	a	0,11
X1978	258216	36984	a	19991	9551	b	0,00
X1987	4171198	916837	a	0	0	b	0,00
X1991	662020	169226	b	851405	285321	a	0,57
X1992	76441337	3925367	a	966254	206982	b	0,00
X1993	9845361	976977	a	56349	18467	b	0,00
X1995	6647	2455	b	2234178	1811416	a	0,22
X1996	3209	1442	b	3973799	1680275	a	0,02
X1997	24130346	7642821	a	5853	4013	b	0,00
X2002	2496	1777	b	118196	25740	a	0,00
X2003	6667765	1571591	a	1038	756	b	0,00
X2005	83565002	10746637	a	14000	8705	b	0,00
X2006	2727212	440932	a	34230	10083	b	0,00
X2007	638110	71843	a	5422	1798	b	0,00
X2010	902	291	b	200574	199360	a	0,32
X2011	38323	17601	b	2339058	765032	a	0,00
X2012	936968	536959	a	88	62	b	0,09
X2017	2173	747	b	404957	135464	a	0,00
X2018	1140844	228777	a	8549	6655	b	0,00

X2021	16391535	2306599	a	246586	70357	b	0,00
X2022	39680243	2910766	a	329591	85065	b	0,00
X2027	8832583	2608028	a	92713	47676	b	0,00
X2029	73476	15542	a	15559	4949	b	0,00
X2030	445081	108631	a	10410	3856	b	0,00
X2031	127396	51924	b	184849	78832	a	0,55
X2033	6826338	773928	a	19590	11212	b	0,00
X2034	12594080	3705993	a	21046	16745	b	0,00
X2036	1535308	487593	a	9318	8433	b	0,00
X2040	14746378	5327722	a	9965	5962	b	0,01
X2042	1909181	718403	b	2692885	1179859	a	0,57
X2046	403951	162743	b	637568	276964	a	0,47
X2047	28840557	2317858	a	253865	90133	b	0,00
X2049	4584867	525413	a	64605	20497	b	0,00
X2052	1406955	333806	a	20388	9738	b	0,00
X2053	16517103	377982	a	2064018	534261	b	0,00
X2056	413233	38778	a	5800	2460	b	0,00
X2059	13972888	1930093	a	26860	17437	b	0,00
X2061	7267943	1227957	a	100824	30736	b	0,00
X2062	2925492	415940	a	9541	5028	b	0,00
X2063	2238925	201086	a	1257	546	b	0,00
X2064	5892753	914016	a	0	0	b	0,00
X2066	209332	146549	a	6516	4464	b	0,17
X2070	43626	14100	a	212	130	b	0,00
X2071	19772	11497	a	0	0	b	0,09
X2072	3450	1638	a	243	181	b	0,06
X2076	100451037	3768128	a	1937871	446483	b	0,00
X2080	20968544	866841	a	210556	52402	b	0,00
X2082	182981415	11962495	a	3343690	665233	b	0,00
X2084	843069	108955	a	8836	3652	b	0,00
X2087	41054	15088	a	2139	1216	b	0,01
X2089	40693544	2702640	a	582936	129604	b	0,00
X2090	4639691	397262	a	51061	13253	b	0,00
X2093	825021	131084	a	7282	3790	b	0,00
X2094	11801513	1798548	a	7830951	1605413	b	0,10
X2095	442950	61918	a	22075	9885	b	0,00
X2097	118738410	8970021	a	890633	270880	b	0,00
X2099	3504563	464561	a	45150	13923	b	0,00
X2101	1103576	195050	a	862365	197468	b	0,39
X2102	54907	13314	a	0	0	b	0,00
X2103	4906	993	b	95145	31895	a	0,01

X2104	22399647	2079933	a	76241	27292	b	0,00
X2107	3352984	294342	a	41312	12191	b	0,00
X2108	56009	12482	a	16314	9581	b	0,01
X2112	3150029	226440	b	3209467	286124	a	0,87
X2114	469403	126341	a	13072	4175	b	0,00
X2123	6176214	1179806	a	52030	14858	b	0,00
X2125	588081	157705	a	2289	1025	b	0,00
X2128	3093212	509693	a	7869	4261	b	0,00
X2132	7494639	1900620	a	0	0	b	0,00
X2135	61691508	10110407	a	254170	94886	b	0,00
X2138	3594812	489783	a	15052	8301	b	0,00
X2140	15627237	1446223	a	189175	45914	b	0,00
X2141	5387029	666153	a	81551	42292	b	0,00
X2143	1602261	148555	a	29938	9685	b	0,00
X2145	71448	23307	a	5342	2410	b	0,01
X2149	1482221	193920	a	7067	2134	b	0,00
X2150	837343	142966	a	3784	2941	b	0,00
X2151	53478351	4016252	a	399510	87479	b	0,00
X2153	360482	53370	a	245574	76440	b	0,22
X2155	12140588	950018	a	76846	18697	b	0,00
X2156	5745	2057	a	1199	788	b	0,04
X2162	25629	6759	b	42562	14967	a	0,31
X2163	69186	12650	a	38038	21794	b	0,22
X2167	5624	2076	b	12116	4020	a	0,16
X2169	17029	3351	a	0	0	b	0,00
X2170	3051	808	a	0	0	b	0,00
X2173	956603	181603	a	8112	2326	b	0,00
X2174	962042	128534	a	20089	6681	b	0,00
X2178	4650	1759	b	28045	12181	a	0,06
X2180	237499	26241	a	1367	644	b	0,00
X2182	0	0	b	4729	1901	a	0,02
X2186	63200	46237	a	404	256	b	0,18
X2187	3462296	495712	a	5356	1964	b	0,00
X2189	20522	13364	b	317498	143298	a	0,04
X2194	23143453	1538993	a	2582136	554410	b	0,00
X2199	7798885	1011707	a	914680	204276	b	0,00
X2200	42337427	7840158	a	379754	149883	b	0,00
X2203	7470801	760817	a	953423	203148	b	0,00
X2204	3227	1749	b	14317	9971	a	0,28
X2211	1582309	169582	a	911	587	b	0,00
X2216	11228465	1589171	a	0	0	b	0,00

X2218	948506	195920	a	4906	2569	b	0,00
X2220	4433462	734232	a	0	0	b	0,00
X2221	1963021	410290	a	53662	16084	b	0,00
X2227	450083	95677	a	8139	2735	b	0,00
X2230	484556	134424	a	21568	8303	b	0,00
X2233	6411069	449503	a	34632	9535	b	0,00
X2234	91935	35182	a	3308	1636	b	0,01
X2237	1065	559	b	2709968	865502	a	0,00
X2240	0	0	b	509170	140764	a	0,00
X2242	1670876	432690	a	1547	948	b	0,00
X2243	437692	105577	a	65596	27907	b	0,00
X2251	1140338	120496	b	6356437	744011	a	0,00
X2254	26684862	2184362	a	77067	20489	b	0,00
X2257	1118990	389250	a	0	0	b	0,01
X2258	5332272	702239	a	10816	3570	b	0,00
X2261	19125392	1666597	a	78584	27972	b	0,00
X2262	15309533	5201287	a	162891	80066	b	0,01
X2264	109492	24002	a	879	718	b	0,00
X2267	4035266	363316	a	15217	6297	b	0,00
X2269	2452027	1055203	a	16089	6925	b	0,02
X2272	2009197	336204	a	3366	939	b	0,00
X2273	1648466	226565	a	3711	1858	b	0,00
X2274	24206445	3007291	a	28966	13555	b	0,00
X2276	1248666	446190	a	29155	13029	b	0,01
X2278	5074772	744556	a	25515	8284	b	0,00
X2279	67982	18389	a	278	206	b	0,00
X2280	146749	19038	a	0	0	b	0,00
X2281	0	0	b	33854	12718	a	0,01
X2282	246174	34335	a	10017	3629	b	0,00
X2284	10083	3793	a	5607	3909	b	0,41
X2290	7288912	1194112	a	0	0	b	0,00
X2299	6741641	1308686	a	0	0	b	0,00
X2300	11560264	1400069	a	50639	12351	b	0,00
X2301	11643	2649	b	6432380	511608	a	0,00
X2304	1631780	252679	a	13256	7421	b	0,00
X2306	2855486	441393	a	8001	2994	b	0,00
X2310	122437	25877	a	40399	19001	b	0,01
X2311	8632386	705275	a	246274	46830	b	0,00
X2312	868493	295043	a	2401	1916	b	0,00
X2313	2299135	253804	a	3037	1110	b	0,00
X2315	8659	2524	a	5752	3429	b	0,50

X2316	2237782	196456	a	54973	12913	b	0,00
X2319	45989	12792	a	357	259	b	0,00
X2320	1923246	138017	a	46954	9957	b	0,00
X2322	5353524	786622	a	49946	15659	b	0,00
X2325	1142967	170882	a	6954	3128	b	0,00
X2326	3541575	1729141	a	1190	956	b	0,05
X2327	4803537	513616	a	67411	16786	b	0,00
X2329	5760599	803706	a	90959	26544	b	0,00
X2330	93237	31842	a	0	0	b	0,00
X2332	894896	168861	a	13203	6112	b	0,00
X2338	261828	18940	a	1431	823	b	0,00
X2340	3387597	437038	a	6022	2269	b	0,00
X2348	13650227	2074736	a	0	0	b	0,00
X2349	18380356	4705760	a	0	0	b	0,00
X2355	10489832	629877	a	0	0	b	0,00
X2361	0	0	b	6039537	1050925	a	0,00
X2362	9063621	1114367	a	5445	2465	b	0,00
X2363	4551953	826328	a	0	0	b	0,00
X2366	29995	11637	a	1153	636	b	0,02
X2369	2308128	232850	a	8668	4770	b	0,00
X2372	11901915	1428424	a	35721	13368	b	0,00
X2374	9124579	1175221	a	18351	8583	b	0,00
X2378	2654536	304467	a	4663	2157	b	0,00
X2379	136060	24686	a	790	427	b	0,00
X2381	4261160	550836	a	12521	5105	b	0,00
X2382	10597439	1571811	a	31300	10369	b	0,00
X2386	265725	89280	b	6454447	1482904	a	0,00
X2388	1165906	120478	a	9536	2234	b	0,00
X2390	53862	23431	b	1522835	438740	a	0,00
X2393	4994	2315	b	171590	54785	a	0,00
X2402	9093105	3072609	a	0	0	b	0,00
X2403	1101497	399531	a	7541	4665	b	0,01
X2405	19981487	4424158	a	0	0	b	0,00
X2410	3569598	839164	a	0	0	b	0,00
X2412	18740555	4666301	a	0	0	b	0,00
X2417	103751694	8471951	a	649834	202203	b	0,00
X2419	7353806	811939	a	179451	54501	b	0,00
X2421	26481149	2303108	a	133385	47022	b	0,00
X2425	3964973	412460	a	13933	6006	b	0,00
X2428	9389	3857	a	1359	1198	b	0,05
X2432	4479423	1495544	a	86627	24904	b	0,00

X2435	2551162	696619	a	478604	258295	b	0,01
X2450	382646	66412	a	120668	52898	b	0,00
X2451	30891508	3644539	a	0	0	b	0,00
X2452	59730781	7726900	a	0	0	b	0,00
X2456	66053	17199	a	23939	12346	b	0,05
X2458	4960273	1109473	a	0	0	b	0,00
X2464	2541826	697987	a	24472	9681	b	0,00
X2466	2195080	465926	a	3878	1551	b	0,00
X2467	1130448	175584	a	11841	3186	b	0,00
X2470	200827	31373	a	5719	3398	b	0,00
X2472	11862879	968815	a	12299	6211	b	0,00
X2473	267898	28438	a	997	472	b	0,00
X2474	155349	20049	a	6829	4010	b	0,00
X2476	1167037	193736	a	5113	2243	b	0,00
X2478	47618204	2658199	a	33508	19476	b	0,00
X2479	14280074	1065951	a	4336	2749	b	0,00
X2483	8136413	2056279	a	206057	102331	b	0,00
X2485	168203	67134	b	221433	86433	a	0,63
X2488	5779314	2682739	a	5871	5235	b	0,04
X2492	36641	16121	b	58799	24502	a	0,45
X2497	20869236	1655844	a	593006	141522	b	0,00
X2498	6102228	549222	a	41754	11804	b	0,00
X2500	5841181	466661	a	156502	39987	b	0,00
X2501	1937957	307020	a	0	0	b	0,00
X2502	1454220	203063	a	6245	2495	b	0,00
X2503	3805851	312319	a	140382	31275	b	0,00
X2505	1022275	99291	a	31659	7742	b	0,00
X2507	27569	9603	a	0	0	b	0,01
X2508	943666	390488	a	216614	100064	b	0,08
X2509	6147711	629247	a	200961	43451	b	0,00
X2510	0	0	b	150344	66146	a	0,03
X2511	1741748	178257	a	44085	11237	b	0,00
X2512	1512050	189671	a	5187	2279	b	0,00
X2515	594397062	29345527	a	2058252	684533	b	0,00
X2522	26573045	1458080	a	16159	5312	b	0,00
X2526	53270646	11720219	a	0	0	b	0,00
X2528	13740280	3384298	a	0	0	b	0,00
X2529	2081615	353926	a	0	0	b	0,00
X2531	7308316	1923345	a	4882	4257	b	0,00
X2532	368441	66339	a	0	0	b	0,00
X2535	14147414	1749748	a	168844	48187	b	0,00

X2536	170121884	15887943	a	0	0	b	0,00
X2537	20860234	5573188	a	0	0	b	0,00
X2538	9018120	497510	a	51510	16750	b	0,00
X2539	7870582	1096407	a	75815	23228	b	0,00
X2540	1267752	201618	a	532	297	b	0,00
X2542	16849930	2218886	a	155293	42206	b	0,00
X2545	44903794	4477723	a	0	0	b	0,00
X2549	4227430	564305	a	23366	9730	b	0,00
X2550	3204614	915112	a	0	0	b	0,00
X2552	29662679	3837769	a	385036	101979	b	0,00
X2558	11941856	1150536	a	110093	26530	b	0,00
X2560	2524227	248742	a	21048	6011	b	0,00
X2564	11786	5031	b	90998	53679	a	0,15
X2565	2259803	665036	a	1290	656	b	0,00
X2567	19660691	2721526	a	0	0	b	0,00
X2571	3551	1128	b	12203	7152	a	0,24
X2573	729666386	16515825	a	44025670	8077526	b	0,00
X2576	215886495	4925327	a	12292397	2322025	b	0,00
X2577	4407502	814442	a	0	0	b	0,00
X2579	48081683	1168273	a	2049429	421416	b	0,00
X2580	24921419	2408740	a	386727	93815	b	0,00
X2582	5488979	250298	a	281022	64308	b	0,00
X2583	5985288	672542	a	83401	24352	b	0,00
X2593	341130	143762	b	475209	189545	a	0,58
X2597	5241605	877352	a	0	0	b	0,00
X2598	82540	36996	b	122684	50547	a	0,52
X2607	35834453	2326820	a	4148391	1573892	b	0,00
X2608	7833502	1198339	a	0	0	b	0,00
X2610	5091303	685138	a	3773	2204	b	0,00
X2611	9766337	763606	a	947195	432675	b	0,00
X2614	75251768	6878534	a	231666	70957	b	0,00
X2615	1807666	139720	a	172271	80895	b	0,00
X2617	20374201	2052503	a	34422	10798	b	0,00
X2618	2842181	1390910	a	0	0	b	0,05
X2619	6173732	281028	a	350760	92298	b	0,00
X2620	0	0	b	43915	24539	a	0,08
X2624	1274532	228243	a	0	0	b	0,00
X2629	46018	6416	a	0	0	b	0,00
X2645	0	0	b	1269962	439356	a	0,01
X2646	9597304	2251788	a	0	0	b	0,00
X2651	104120	28878	b	362783	140748	a	0,08

X2652	50458094	3342668	a	0	0	b	0,00
X2653	3380807	925786	a	0	0	b	0,00
X2655	6298818	587088	a	37130	10889	b	0,00
X2656	16619	6593	b	111230	44915	a	0,04
X2657	12588846	1074420	a	0	0	b	0,00
X2659	601	324	b	4592	2400	a	0,10
X2660	4709400	1276033	a	0	0	b	0,00
X2665	43631	19354	b	79702	35080	a	0,37
X2669	31006	22484	b	64312	40364	a	0,47
X2671	5179361	1922275	a	0	0	b	0,01
X2672	835893	89723	a	7382	4159	b	0,00
X2675	2039941	646037	a	1250	506	b	0,00
X2686	127555482	10581502	a	511789	144647	b	0,00
X2690	49172033	7071007	a	0	0	b	0,00
X2691	20589276	5334654	a	0	0	b	0,00
X2695	2713384	290913	a	10256	3575	b	0,00
X2698	61375400	6102717	a	0	0	b	0,00
X2704	635258	137100	a	309605	86723	b	0,05
X2709	824919	82957	a	24343	12222	b	0,00
X2710	9011926	867169	a	35907	11285	b	0,00
X2715	2724909	1331716	a	0	0	b	0,05
X2720	110626	44397	a	92977	45139	b	0,78
X2724	6935663	2263186	a	4816	2369	b	0,00
X2728	10563312	1629040	a	0	0	b	0,00
X2736	21347492	1897774	a	0	0	b	0,00
X2737	49269361	5131648	a	0	0	b	0,00
X2742	12363082	1526410	a	0	0	b	0,00
X2743	11148019	2659119	a	0	0	b	0,00
X2745	3043257	796012	a	0	0	b	0,00
X2750	12342662	1729075	a	0	0	b	0,00
X2751	4152177	291349	a	49393	14000	b	0,00
X2754	1033855	144536	a	5156	1706	b	0,00
X2757	0	0	b	128135	70874	a	0,08
X2763	387	272	b	65474	27706	a	0,02
X2767	18985507	4770202	a	0	0	b	0,00
X2772	6434401	758619	a	2249	1464	b	0,00
X2783	9132438	1030157	a	0	0	b	0,00
X2787	5477414	1185753	a	0	0	b	0,00
X2788	871644	68573	a	64190	35953	b	0,00
X2798	18865431	1950162	a	0	0	b	0,00
X2805	16130120	2218172	a	0	0	b	0,00

X2806	10238206	2647116	a	0	0	b	0,00
X2813	4410914	1811328	a	4250	3935	b	0,02
X2814	15733671	2508788	a	0	0	b	0,00
X2823	7656273	1145518	a	0	0	b	0,00
X2827	10278410	2437439	a	12518	5013	b	0,00
X2829	2448597	681601	a	0	0	b	0,00
X2831	13416194	3558564	a	1148	1060	b	0,00
X2834	3089493	802033	a	0	0	b	0,00
X2836	5463777	1647003	a	38282	28711	b	0,00
X2840	1558645	498050	a	11357	9923	b	0,00
X2853	358607167	14496400	a	1470342	576402	b	0,00
X2855	21876663	1072714	a	0	0	b	0,00
X2856	17556825	4111503	a	0	0	b	0,00
X2858	5898780	699911	a	25472	10199	b	0,00
X2861	23186972	1844781	a	0	0	b	0,00
X2863	4589856	722389	a	0	0	b	0,00
X2867	2901424	362040	a	10790	4711	b	0,00
X2872	310350	140645	b	833878	459515	a	0,28
X2878	138227	63375	b	258821	114437	a	0,36
X2883	13542534	1925391	a	0	0	b	0,00
X2891	3542165	993179	a	0	0	b	0,00
X2895	6624828	1500608	a	0	0	b	0,00
X2903	11631103	2517571	a	0	0	b	0,00
X2904	68020	14540	a	0	0	b	0,00
X2905	87801304	5094945	a	36381	19026	b	0,00
X2907	2323820	612403	a	0	0	b	0,00
X2908	33355792	1977612	a	11443	6507	b	0,00
X2911	8056037	894879	a	0	0	b	0,00
X2915	7353635	3556759	a	31150	16197	b	0,04
X2917	15890436	1556350	a	0	0	b	0,00
X2920	1043379	751184	a	7436	4205	b	0,17
X2923	1240288	574580	a	8065	4982	b	0,04
X2926	6293477	1833182	a	0	0	b	0,00
X2930	2867746	187919	a	16747	6930	b	0,00
X2937	113597021	43426096	a	632440	339854	b	0,01
X2941	40400535	15490144	a	209868	92873	b	0,01
X2942	9244483	3911818	a	5860	1942	b	0,02
X2943	7397013	2478888	a	0	0	b	0,00
X2945	1124110	546522	a	4215	3454	b	0,04
X2947	7014924	1143214	a	592514	165423	b	0,00
X2948	1561314	290398	a	195013	58121	b	0,00

X2953	42523	23756	a	33112	15237	b	0,74
X2960	22159180	3036900	a	24080	14859	b	0,00
X2962	1614306	250287	a	20922	6091	b	0,00
X2970	12551851	2861506	a	0	0	b	0,00
X2973	3859616	1024803	a	0	0	b	0,00
X2981	8316096	1347630	a	8710	3332	b	0,00
X2983	2190066	367896	a	1630	732	b	0,00
X2985	4885854	425343	a	3961	1350	b	0,00
X2996	4470707	693962	a	67869	25946	b	0,00

Table S3. Deconvoluted total intensities (mean ± S.D.) of all identified in flowers organ and epiphytic. The statistically significant differences between organ and epiphytic detected by Tukey's

HSD post-hoc tests are indicated by bold letters (*P*< 0.05). Metabolites as in Fig. 1 caption, the X represent the unknown metabolites.

Flowers							
**Metabolites**	**Organ**			**Epiphytic**			
	**Mean**	**S.D.**		**Mean**	**S.D.**		**Pr(>F)**
Ala	608080	88710	b	2970857	433381	a	0,00
Arg	161102	57860	b	1944535	1087024	b	0,11
Asn	11273001	4619509	b	13988279	3643229	a	0,65
Asp	6111352	378513	a	0	0	b	0,00
Gln	320989	32400	b	21842404	6118521	a	0,00
Glu	5260092	445800	a	953571	259338	b	0,00
Glup	26483866	3824373	a	23687751	4606656	a	0,64
His	103058	24218	b	518767	224263	a	0,07
HPro	1787	1499	b	42114	6554	b	0,00
Iso	66304950	5023551	a	5453917	3577516	a	0,00
Lys	137841	41414	b	809205	742535	a	0,37
Met	328729	96576	b	2449185	1484102	b	0,16
Phe	77295029	5281840	a	5421191	2880184	b	0,00
Pro	7893161	1397210	b	10945548	1224818	a	0,11
Ser	737298	258349	b	3601027	1456982	b	0,06
Thr	26293768	2820350	a	5640909	2114969	b	0,00
Try	79880069	13365419	a	562351	163524	b	0,00
Tyr	336661237	3768038	a	41668671	5063853	a	0,00
Val	31126179	2641375	b	37266142	7132504	b	0,42
Ad	55380791	14138332	a	357297	195472	a	0,00
Ade	0	0	b	491953	411917	a	0,24
AMP	218497	92587	b	678504	234121	a	0,07
Cy	109237	50042	a	38082	10814	b	0,17
Gua	1480092	911663	a	50928	15093	b	0,12
Ur	1025442	404939	a	48286	14082	b	0,02
Dis	6818610	598329	a	5229512	840120	b	0,13
Hex	27206522	3369976	a	9894239	814257	a	0,00
Pen	4725204	605542	a	433676	39361	a	0,00
Raf	37052	7295	b	22545987	5061247	a	0,00
Xyl	3498087	585723	b	3605594	743752	a	0,91
AbA	1185314	155821	a	35218	13144	b	0,00
Ani	1637618	579697	a	1251811	485065	b	0,61
AsA	9369389	1696343	a	201996	59064	b	0,00
CafA	7682645	693849	a	48221	20348	b	0,00
Car	505615	194329	b	9989737	1806789	b	0,00
Cho	394673069	18189622	a	30811481	2154768	b	0,00

Cit	60008580	5569428	a	7749005	2984084	a	0,00
JaA	83464	23742	a	8972	4056	b	0,00
Lac	9342772	1211187	b	16791384	2732532	a	0,02
Log	1861718	277892	a	38023	7554	b	0,00
Mal	318524792	20947015	a	34105105	6368660	a	0,00
OxA	176157	98098	a	52987	10282	b	0,22
PyA	7183022	849725	a	127877	36248	b	0,00
ShA	3623163	532514	a	623109	96028	b	0,00
SuA	44728936	4392685	a	8364015	1132702	b	0,00
Cat	56827081	960368	a	2581987	475190	b	0,00
CGA	773350753	36238554	a	15193314	2742079	b	0,00
CGAp	288660533	9984471	a	753128	344094	a	0,00
Chr	52708	33723	b	160925	78139	b	0,21
CoA	3492647	500600	a	240766	60184	b	0,00
FeA	45530342	3775678	a	1760266	470260	a	0,00
Fis	218592	73147	a	0	0	b	0,00
Hom	3026063	905191	a	6621	4502	b	0,00
Hom.1	2431043	222165	a	400446	121469	a	0,00
Kae	147738	41717	a	471	343	b	0,00
Pin	1927629	428222	a	224054	47859	b	0,00
Prot	595568	77405	a	39675	6606	b	0,00
Que	3766478	973974	a	1511	697	b	0,00
Rha	17569	5526	a	0	0	b	0,00
Sal	363074	70250	a	315057	78228	b	0,65
Sap	163583485	8133430	a	1616257	372038	b	0,00
SiA	3906764	708481	a	5783	830	b	0,00
VaA	3906710	1077343	a	64207	9876	b	0,00
Nic	1412571	340956	a	0	0	b	0,00
Rib	1059734	105493	a	117084	30026	b	0,00
Sec	33382987	3436009	a	5019025	1393592	b	0,00
Toc	20462346	1018747	a	14848710	1803450	a	0,01
Vi.B1	2574199	566887	a	51937	7592	b	0,00
Vi.B5	220816	36051	a	74353	33452	b	0,00
Vi.B6	558765	108248	a	36916	9282	a	0,00
Vit	20641	7310	a	0	0	b	0,01
X1	203792975	3588958	a	40768932	3121133	a	0,00
X2	11016756	893941	a	840684	126811	b	0,00
X3	14536981	845095	a	949353	136642	b	0,00
X4	17825257	797200	b	38440704	2615409	a	0,00
X5	69715	6806	b	76378	7148	b	0,50
X6	154251517	26406445	a	124532812	14842231	a	0,33

X7	416670	285828	b	179989251	11881234	b	0,00
X8	873284515	34609391	a	133145150	31590598	a	0,00
X9	239680	64282	b	509246	347736	b	0,45
X10	83387441	1208842	a	12836657	1335680	b	0,00
X11	71492348	3200703	b	74699523	3464042	a	0,50
X13	65860141	16788833	a	59709	36845	b	0,00
X14	354940	209174	a	0	0	b	0,10
X16	136073	30532	b	68391403	3362996	a	0,00
X19	1801780	384093	a	0	0	b	0,00
X20	5660916	594933	a	1889643	220107	b	0,00
X21	9403479	802729	a	2071408	267757	b	0,00
X22	2953983658	38089382	a	751848083	45622013	a	0,00
X23	23753939	1682904	a	5126195	710665	b	0,00
X24	7907574	680100	a	1918336	258064	a	0,00
X25	668570	215490	a	1702	745	b	0,00
X26	472890576	7051227	a	87629075	6282984	b	0,00
X27	191352635	9868912	b	454356609	27495259	a	0,00
X28	0	0	b	49035	8094	b	0,00
X29	406584947	5529613	a	100174657	6402949	a	0,00
X30	6881607	983261	a	657180	79823	b	0,00
X31	45435428	16751083	a	15136150	8149276	a	0,11
X32	2208936	1415406	a	844656	685030	b	0,39
X33	3117663	1854188	a	942472	849092	b	0,29
X34	57457481	3350601	a	14578662	1597969	a	0,00
X35	164600	27736	b	6593015	593799	b	0,00
X36	58872511	12157361	b	1692469399	125011659	a	0,00
X37	370388	88792	b	17480242	2041688	a	0,00
X38	5280	3147	b	2881616	251203	b	0,00
X39	23102439	830582	a	705319	83686	a	0,00
X40	297904	50457	b	117120703	5425119	b	0,00
X41	997517002	10748140	a	178864160	14392180	a	0,00
X42	110667296	1228236	a	13951707	2072539	a	0,00
X43	9074	994	a	7906	973	b	0,40
X44	20905847	1858227	b	159729826	10192648	a	0,00
X45	63433	12173	b	29458747	1535945	b	0,00
X46	134152630	1598745	a	20131748	2242676	a	0,00
X47	38084	11648	b	590172	116744	a	0,00
X48	136393	73484	b	30927598	11221195	a	0,01
X49	170802	70287	a	152912	19726	b	0,81
X50	215158	154948	b	637955	240861	b	0,15
X52	4802300	2695341	a	1283244	1011879	a	0,23

X53	165054	43137	a	143012	18738	b	0,64
X55	395490	167298	b	4417916	1134764	a	0,00
X58	2876	1657	b	41599	12153	b	0,00
X59	4428192	425112	b	4532869	390946	b	0,86
X60	117074726	16777162	a	98816247	11608260	a	0,37
X61	46745420	764750	b	51878751	1103372	b	0,00
X62	112391203	5883943	a	89965180	8475520	a	0,03
X63	80050	18341	b	30860823	1934228	a	0,00
X64	42636	10372	b	11931806	834204	a	0,00
X67	4237732	332132	a	1287606	124583	b	0,00
X68	6125356	417747	a	1819024	243120	b	0,00
X69	2494481121	33028896	a	619911717	37327494	a	0,00
X70	34824074	3261095	a	13211928	1831510	a	0,00
X71	6607227	532092	a	1834008	200997	b	0,00
X72	483904733	6411282	a	91938832	7817513	b	0,00
X73	352736972	4331740	a	81501225	5392237	a	0,00
X74	2556218	215625	a	562388	67089	a	0,00
X75	8346	7100	b	1522681	949839	b	0,12
X76	8960213	624779	a	753638	91060	a	0,00
X77	0	0	b	5728109	549437	b	0,00
X78	24464664	2751958	b	26727360	3408994	a	0,61
X79	238958	163636	a	42642	21724	b	0,24
X81	10882459	6187829	a	3661803	2693935	b	0,29
X82	11999849	5906509	a	3901463	2771242	a	0,22
X83	2100	767	b	4453	975	b	0,06
X84	28612466	6873755	b	1121620887	52063105	a	0,00
X85	9302395	2122828	b	9703491	2503551	a	0,90
X86	117481	25836	b	86261589	4357994	a	0,00
X87	25642843	1327948	a	1198726	180751	a	0,00
X88	15703	5464	b	12587536	1038202	a	0,00
X90	6650140	2358773	a	2838758	412880	b	0,12
X92	118760784	1862910	b	120854948	1710299	a	0,41
X95	10148785	815885	a	302306	32786	a	0,00
X96	260451	156280	b	45841982	16616442	a	0,01
X98	3308297	858558	a	72582	9197	a	0,00
X99	17164	4701	b	726566	516784	b	0,18
X100	129568484	16354034	a	102129334	13363753	b	0,20
X101	263419234	3100376	b	281036986	4175082	a	0,00
X102	9922	3561	a	9131	1672	b	0,84
X104	47898	13508	b	17413476	1530838	b	0,00
X105	92982254	1138559	b	100308188	1609220	a	0,00

X106	13494	9425	b	5022390	3358259	a	0,14
X107	241594	76555	a	146803	23691	a	0,24
X109	5812	2256	a	2235	1498	b	0,19
X111	1297823	462999	b	1764642	620430	a	0,55
X112	267017	118742	a	266442	128018	b	1,00
X114	307612	143401	a	57393	31777	a	0,09
X115	13761	9906	b	11767950	1405772	b	0,00
X118	15641928	2177608	a	147870	21712	b	0,00
X119	1243499	261510	a	35856	4289	b	0,00
X120	97447039	1153509	a	4917039	778874	a	0,00
X121	403521	44873	b	493579	50814	a	0,19
X122	0	0	b	130096	31825	b	0,00
X124	183028	47628	a	23486	5758	b	0,00
X125	9911394	2347575	a	3499	1363	b	0,00
X126	39330913	10351044	b	52988778	7275350	a	0,28
X127	423177	337264	a	218246	56602	b	0,55
X128	232226	38627	b	4896285	437921	a	0,00
X129	195171	87374	b	243763	112966	b	0,73
X131	17329773	5094620	a	0	0	b	0,00
X132	25984360	8485759	a	0	0	b	0,00
X133	1020413	519061	a	10471	3718	b	0,06
X135	42331365	13554766	a	52638	6899	b	0,00
X138	1346057	418882	b	4659022	1256461	a	0,02
X140	3785754	1344325	b	4199066	657567	a	0,78
X141	421064	57308	b	854801	812412	b	0,60
X143	766040384	46238012	a	0	0	b	0,00
X145	9697	2342	a	0	0	b	0,00
X147	46132652	3696857	a	0	0	b	0,00
X151	187081	70501	a	150691	27252	a	0,63
X152	1469	910	b	457984	373127	b	0,23
X153	11020674	4131279	a	0	0	b	0,01
X155	118397	30585	b	110973615	6052666	a	0,00
X157	7250501	715902	a	350272	51354	b	0,00
X158	90863384	8083780	a	247933	143865	a	0,00
X159	46840	13389	a	11858	1891	b	0,01
X160	4948914	984532	a	0	0	b	0,00
X161	2308280	451276	a	43603	8194	b	0,00
X164	182773	15485	b	223247	22671	b	0,15
X167	23318942	7038537	a	600238	186792	b	0,00
X171	700189	232123	b	835112	243656	a	0,69
X172	5197	1518	b	11993	2954	b	0,05

X180	1177016	531939	a	16742	6473	b	0,03
X182	62746877	6464652	a	265142	87025	b	0,00
X183	3263432	440942	a	0	0	b	0,00
X185	1465729	240963	a	1547	1262	b	0,00
X190	5920752	799467	a	0	0	b	0,00
X192	2906034228	164326192	a	0	0	b	0,00
X197	245158117	14597564	a	0	0	b	0,00
X198	100061	43115	b	2996163	921459	a	0,00
X201	117045	15112	a	25570	5725	b	0,00
X202	104002	30581	b	70223038	4598196	a	0,00
X209	1196273	289138	a	618666	213441	b	0,11
X211	11838973	1907251	a	2953	980	b	0,00
X212	38684409	8279433	a	25401	7293	b	0,00
X216	1022529	374247	a	12625	5072	b	0,01
X217	6279996	415946	a	688498	110013	a	0,00
X218	31275	6402	a	0	0	b	0,00
X219	5417	2101	b	1020910	540379	a	0,07
X220	304199	77146	a	4933	1235	b	0,00
X221	13917	2966	a	0	0	b	0,00
X223	387112	363076	a	1862	1615	b	0,29
X228	5078788	3659086	a	1519784	1337060	a	0,36
X229	173628	20265	a	98686	8667	b	0,00
X230	9573211	2542982	a	4793556	1434852	a	0,11
X232	5888	2264	a	0	0	b	0,01
X237	203911913	19854660	a	1332122	445228	b	0,00
X240	12614824	1557886	a	31667	10044	b	0,00
X241	1842923	894508	a	153521	92078	a	0,07
X242	25400	4338	a	7471	1775	b	0,00
X243	5227014	1589006	a	802131	489772	a	0,01
X244	58463	16787	b	44992771	2978641	a	0,00
X246	32701	6082	a	0	0	b	0,00
X248	4368508	317897	a	849959	100214	b	0,00
X249	1183045037	15600727	a	216590289	17157645	a	0,00
X250	5361240	462072	a	843223	118857	b	0,00
X252	87041079	4742416	a	3903375	539461	b	0,00
X253	121485390	1693560	a	6853746	993168	b	0,00
X254	65705460	1212772	b	66961253	1045013	a	0,44
X259	3274222	968592	a	5988	2795	b	0,00
X260	1685713	667789	a	4609	1447	b	0,01
X261	28866015	3062773	a	11597	4262	b	0,00
X263	48189321	6239274	a	25562	12199	a	0,00

X264	7623	6343	b	433861	314857	b	0,18
X265	546933	263017	a	689	396	b	0,04
X266	30472527	5348219	a	0	0	b	0,00
X267	20430369	4038424	a	192999	29420	b	0,00
X269	10889470	1713680	a	24394	4644	b	0,00
X274	13884196	3772053	a	10630597	1361313	a	0,42
X276	3082954	279262	b	3098011	381088	a	0,97
X279	148178	21921	a	6535	2305	b	0,00
X281	2036171	309743	a	22558	7528	b	0,00
X284	3425122	1053897	b	582197522	25784593	a	0,00
X285	530758	153079	b	4794575	508384	b	0,00
X287	4802149	505291	b	5639052	602671	a	0,29
X289	30542	8362	b	6877956	530086	b	0,00
X291	15078523	3290351	a	35688	7078	b	0,00
X296	78550456	1757034	a	2464752	377852	b	0,00
X298	57073349	5605465	a	2138058	322849	b	0,00
X301	72923192	9422410	a	69930	46402	b	0,00
X310	3336953	740652	a	25814	3887	b	0,00
X313	918826	226077	a	616627	305841	b	0,43
X315	28995800	3727495	a	46618	5240	b	0,00
X316	84406867	15592627	a	0	0	b	0,00
X319	20334	5379	a	0	0	b	0,00
X321	322597	64337	b	19299791	1871515	a	0,00
X330	47150	10972	a	12342	3342	b	0,00
X332	16584983	4694944	a	1121	558	b	0,00
X335	1007266	160606	a	73608	14031	b	0,00
X337	16762165	3064808	a	6904	2568	b	0,00
X339	260773	43408	b	182779384	9089373	a	0,00
X342	3211186	1420465	a	5958	2495	b	0,03
X343	448370	105273	a	38044	5103	b	0,00
X346	6533178	1260415	a	0	0	b	0,00
X347	54439254	13503284	a	0	0	b	0,00
X350	301911763	28798290	a	2179600	745361	a	0,00
X351	257122	125701	a	4363	4012	b	0,05
X354	548215184	85891846	a	0	0	b	0,00
X356	26499732	3011533	a	89817	34038	b	0,00
X357	17076465	993135	a	738145	103577	b	0,00
X358	52528195	10524441	a	0	0	b	0,00
X359	826626	271332	a	4970	1106	b	0,00
X360	11106311	1820458	b	11513024	1938544	b	0,88
X364	38235971	3735815	a	69483	31429	a	0,00

X366	54197	16843	a	0	0	b	0,00
X368	1003137	670533	a	2822	984	b	0,14
X374	2120839	1119905	a	0	0	b	0,06
X378	4304091	1801994	a	15457	13603	b	0,02
X379	32020534	12619769	a	0	0	b	0,01
X381	36189	20933	a	27947	10855	b	0,73
X384	7650802	2800979	a	1338	1156	b	0,01
X390	16989066	2925303	a	1117	628	b	0,00
X391	35588498	7800093	a	16450751	4245269	a	0,04
X392	4937529	1039817	a	0	0	b	0,00
X393	23256140	5997885	a	0	0	b	0,00
X397	763983	335165	a	0	0	b	0,03
X399	657191079	153780765	a	0	0	b	0,00
X401	96458800	24444150	a	0	0	b	0,00
X404	10758167	1237083	a	541158	82045	b	0,00
X415	16366014	4169637	a	0	0	b	0,00
X420	3281236	1493800	a	7753	2755	b	0,03
X422	238893	58856	b	1228459	378432	b	0,01
X423	11987184	1567868	b	13023597	1787703	a	0,66
X424	9879	6469	b	515832	131569	a	0,00
X426	107783	22977	b	22305535	1794553	a	0,00
X427	533627	258455	a	0	0	b	0,04
X429	0	0	b	443118	56559	a	0,00
X430	47520	7477	b	54755	7886	b	0,51
X433	13824429	2584890	a	35431	8311	b	0,00
X436	41311	9064	a	0	0	b	0,00
X437	70817	9874	b	1677340	748636	a	0,04
X439	119792	27952	a	767	443	b	0,00
X441	9832636	668180	a	466982	81480	a	0,00
X445	139901	68415	a	0	0	b	0,05
X458	3283103	1283773	a	8646	4118	a	0,01
X460	0	0	b	13742188	2260863	a	0,00
X461	15808	5476	b	4087408	1446843	b	0,01
X462	11500989	2122483	a	313754	36252	a	0,00
X463	0	0	b	259979	113885	b	0,03
X464	1949252	805431	a	0	0	b	0,02
X465	1881743	555851	a	6371	3488	b	0,00
X467	78856	15125	b	3602783	824939	a	0,00
X470	28035	7950	b	415162	238505	a	0,11
X471	95194	21303	b	199348	49084	b	0,06
X474	325355	76666	b	9812046	954495	a	0,00

X480	6599174	688913	b	11168836	504972	a	0,00
X485	5245060	1092831	a	795	553	b	0,00
X489	676054	82812	b	6938326	2204862	a	0,01
X490	6158504	1249832	a	0	0	b	0,00
X491	423516	182271	a	28599	9028	a	0,03
X495	1844	991	b	357201	283319	b	0,21
X496	632668	323137	a	11804	3480	b	0,06
X499	9013664	2200231	a	0	0	b	0,00
X500	1392	852	b	1404409	758872	a	0,07
X502	50726	10997	b	84466	30953	a	0,31
X504	45748	10037	a	21889	7910	b	0,07
X505	103962	31179	a	0	0	b	0,00
X509	41536	9711	b	134663	24440	b	0,00
X510	19113524	2478590	a	0	0	b	0,00
X511	1442619	293629	a	65273	35926	b	0,00
X513	7147478	1879266	a	0	0	b	0,00
X528	12096	6254	a	3534	2136	b	0,20
X536	484684	116438	a	0	0	b	0,00
X538	3442092	1332200	a	126707	27499	a	0,02
X540	8512	2745	a	612	432	b	0,01
X542	163474	38655	a	9711	3381	b	0,00
X543	99733	21783	a	5716	2301	b	0,00
X545	203419	38769	a	4224	1375	b	0,00
X546	22787	6206	a	5371	1375	b	0,01
X547	4751654	1195677	a	0	0	b	0,00
X552	4471888	1093280	a	1291	725	a	0,00
X567	202	115	b	7614	1304	b	0,00
X568	11477085	2075080	a	0	0	b	0,00
X569	20239892	4627230	a	0	0	b	0,00
X571	7973128	1578621	a	0	0	b	0,00
X573	443199	50700	b	1994724	559262	a	0,01
X576	34936	6090	b	119841	27130	a	0,00
X579	0	0	b	929420	354415	b	0,01
X586	9215929	1117659	a	0	0	b	0,00
X600	151399	44029	b	8284343	1134217	a	0,00
X601	724607	724358	a	7899	3111	b	0,33
X605	81482	25523	b	14558766	1935137	a	0,00
X606	351412	69752	a	7417	2037	b	0,00
X613	70467043	10828752	a	0	0	b	0,00
X614	69196	13497	b	565259	399673	b	0,22
X615	20837286	2430702	a	7926	6458	b	0,00

X617	8166122	2414977	a	0	0	b	0,00
X622	396772	108613	a	33556	10042	b	0,00
X632	34044860	5033974	a	0	0	b	0,00
X638	3416863	901475	a	0	0	b	0,00
X641	37508601	12247229	a	0	0	b	0,00
X642	10250868	2307260	a	0	0	b	0,00
X648	302852	75680	a	0	0	b	0,00
X650	1054	448	a	897	456	b	0,81
X653	27752823	2567082	a	0	0	b	0,00
X663	16646825	3857173	a	0	0	b	0,00
X665	3194499	509469	a	83543	16247	a	0,00
X666	0	0	b	22879	6825	b	0,00
X676	209466	194683	a	4789	1101	b	0,30
X682	7056	2791	b	2507745	382342	a	0,00
X690	196433	72178	a	0	0	b	0,01
X711	144335667	11700517	a	180097	66172	b	0,00
X714	24001081	2533036	a	21709	9063	b	0,00
X717	67996	21027	b	73058	29127	a	0,89
X720	3401	1432	b	5863	3714	b	0,54
X722	143309	39744	a	0	0	b	0,00
X725	3823122	1750746	a	198199	119484	b	0,04
X728	610894	324763	a	21986	14238	b	0,08
X729	1931697	971288	a	286044	153828	a	0,10
X731	84024	31766	a	53081	18349	a	0,40
X732	32547	19764	a	1062	692	b	0,12
X736	4344677	536202	a	3790728	570568	b	0,48
X747	17040994	5692466	a	186292	57197	b	0,00
X750	45175752	3625880	a	5266	1856	b	0,00
X751	1064575	957655	a	8718	2030	a	0,27
X752	1034	719	b	50509	15814	b	0,00
X753	8019838	1067120	a	0	0	b	0,00
X755	7068	2260	b	557652	283509	a	0,06
X756	64865	26672	a	0	0	b	0,02
X757	806	581	b	37938	22533	b	0,10
X764	18096805	1317397	a	22892	9126	b	0,00
X765	200690	56970	a	1652	733	b	0,00
X766	101256	32830	a	0	0	b	0,00
X767	28072574	7306007	a	484828	269359	b	0,00
X768	4843308	1676342	a	69551	34910	b	0,01
X769	14981625	4994102	a	615286	251142	b	0,01
X770	2543649	1033524	a	91707	36304	b	0,02

X771	1272765	1152767	a	34278	6348	b	0,29
X774	5417778	734564	b	6298645	956998	a	0,47
X775	0	0	b	2345820	673323	b	0,00
X776	7942584	812197	a	2033	1279	b	0,00
X777	920630	258823	a	0	0	b	0,00
X779	3601399	513928	a	37283	12825	b	0,00
X780	1437444	470651	a	0	0	b	0,00
X781	2047377	596415	a	830	583	b	0,00
X782	58222	23735	b	3177663	1411577	a	0,03
X783	6581	2687	b	656865	257902	a	0,01
X784	635	442	b	52376	17889	a	0,01
X785	10640	2369	b	2566550	1904385	b	0,18
X787	30623250	3672276	a	0	0	b	0,00
X788	3030803	747070	a	0	0	b	0,00
X792	3364349	916952	b	3864832	602765	a	0,65
X793	0	0	b	38735	21052	b	0,07
X796	10922116	1753771	a	41691	21118	b	0,00
X797	883113	418948	a	9442	5186	b	0,04
X798	84884512	4860135	a	44623	24509	b	0,00
X799	188079	65541	a	0	0	b	0,01
X800	16631234	1395833	a	6899	3140	b	0,00
X803	931165	282641	a	532	373	b	0,00
X804	1943435	753236	a	2149	478	b	0,01
X805	7094721	1309270	a	91622	34811	b	0,00
X806	379674896	10811158	a	3702026	1145142	b	0,00
X807	102123974	3224349	a	238504	143700	b	0,00
X808	8761771	944742	a	49613	18568	b	0,00
X810	3960628	578772	a	3485133	493707	a	0,53
X811	292630	164224	a	2235	656	b	0,08
X812	9419570	1258050	a	33776	18886	b	0,00
X813	1049211	247246	a	305	212	b	0,00
X814	5417204	1067564	a	17731	13483	b	0,00
X817	6477186	1701593	a	1165	582	b	0,00
X818	23931210	4230406	a	10286	3635	b	0,00
X819	29689079	5020234	a	17841	6248	b	0,00
X820	23050141	4318740	a	8014	2834	b	0,00
X821	11680212	2573027	a	8047	2965	b	0,00
X822	4268668	1165667	a	1300	913	a	0,00
X825	0	0	b	39790	19607	b	0,05
X826	15444686	1177940	a	0	0	b	0,00
X829	860861	324720	a	0	0	b	0,01

X830	1738200	386554	a	0	0	b	0,00
X831	2506809	708361	a	0	0	b	0,00
X835	17225098	7627154	a	0	0	b	0,03
X1000	2818989	381036	a	262385	61870	b	0,00
X1002	2486943	669127	a	245283	48811	b	0,00
X1003	21853189	3386032	a	860211	162233	b	0,00
X1004	1193998	594704	a	16387	1993	b	0,05
X1005	13418387	2507664	a	79239	24764	b	0,00
X1006	381213	58319	b	10052137	1613396	a	0,00
X1007	2187831	1593144	b	71295281	8479105	a	0,00
X1008	7603993	1915017	a	454475	96227	a	0,00
X1010	379423	105280	a	308051	88884	b	0,61
X1012	1575677	221207	a	218049	25079	b	0,00
X1013	1911895	294556	a	779149	120570	b	0,00
X1014	135938998	13426811	a	3416520	714652	a	0,00
X1015	1691753	348577	a	1423053	282801	b	0,55
X1017	6325395	540824	a	418564	172303	b	0,00
X1018	1462055	457130	a	13280	1665	b	0,00
X1019	1495916	218469	a	640560	288713	a	0,02
X1020	12997	4056	b	20304	4452	b	0,23
X1022	185885719	21205844	a	73412422	13665080	a	0,00
X1023	14590301	1631082	a	7850583	986669	a	0,00
X1024	2530204	257596	b	2934549	402350	b	0,40
X1025	7063599	1406645	a	2483975	282348	a	0,00
X1026	924558	142892	a	235229	23411	b	0,00
X1027	924642	202400	a	623490	132746	a	0,22
X1028	313903	48781	a	228908	30990	b	0,15
X1030	704702	176976	a	40420	3586	b	0,00
X1033	122539	24693	b	561230	327859	b	0,19
X1035	4957112	633822	a	366227	97072	b	0,00
X1036	2833043	593740	a	168906	35051	b	0,00
X1037	442422	82622	a	60955	16150	b	0,00
X1039	588355	132841	a	3449	3178	b	0,00
X1040	2754130	497829	a	45120	10518	b	0,00
X1041	284631	58424	a	13488	1760	b	0,00
X1043	4737633	642818	a	18642	2396	b	0,00
X1044	14242688	1675415	a	26258	6430	b	0,00
X1045	40408264	6132446	a	2543893	721346	b	0,00
X1046	2871532	386750	a	69133	7983	b	0,00
X1047	1740398	323413	a	164305	54342	b	0,00
X1048	2959662	213804	a	70685	7196	b	0,00

X1049	8883661	887950	a	761627	393953	b	0,00
X1051	3388033	557849	a	351422	40128	b	0,00
X1052	15453115	2269854	a	1099979	183029	b	0,00
X1053	25706145	882926	b	29192143	512056	a	0,00
X1056	45684	7138	a	29545	4522	a	0,06
X1057	14386	8135	a	2027	1798	b	0,14
X1058	257249	39431	b	866934	177970	b	0,00
X1059	900807	356822	a	15082	4520	a	0,02
X1061	0	0	b	7492	2802	b	0,01
X1062	120612729	7313543	a	3952817	802264	a	0,00
X1066	1927274	368251	a	24891	2789	b	0,00
X1067	32618	2761	a	26044	3440	b	0,14
X1068	3147950	396714	a	43158	5227	b	0,00
X1069	85137	11166	a	46445	4167	b	0,00
X1070	242991	80602	a	54599	6063	b	0,02
X1071	14604225	1577537	a	2295	885	b	0,00
X1072	330783	75029	a	5168	1041	a	0,00
X1074	3185	967	b	46629	7696	b	0,00
X1075	110524	20951	a	49031	30062	b	0,10
X1076	1868717	244787	a	109899	36518	a	0,00
X1077	644	461	b	222790	60568	a	0,00
X1078	101475	41179	a	47184	13875	b	0,22
X1079	1050604	317213	a	3766	675	b	0,00
X1080	2426861	737314	a	59646	5987	b	0,00
X1081	105859	15165	b	297138	98755	a	0,06
X1082	229258	93287	a	103602	28706	b	0,20
X1083	1210825	319231	a	62640	13351	b	0,00
X1084	317457	67824	a	14074	6387	b	0,00
X1085	2963230	364115	a	13297	3585	b	0,00
X1086	44134163	2052298	a	280960	43462	b	0,00
X1087	8570817	1149011	a	111999	19902	a	0,00
X1088	94746	10774	a	58736	7519	b	0,01
X1089	6800470	946912	a	478473	101225	a	0,00
X1091	80485	11737	a	24050	2376	b	0,00
X1092	57603513	4746921	a	61138	17242	b	0,00
X1093	623847	173132	a	6150	2462	b	0,00
X1095	58776	7337	b	334996	140161	b	0,05
X1096	641909	74319	a	284299	207713	b	0,11
X1097	1767550	319836	a	1362599	279255	a	0,34
X1101	51661	7184	b	426073	100329	b	0,00
X1103	3473287	1279118	a	657476	153969	b	0,03

X1106	1268708	204372	a	16300	6722	b	0,00
X1107	25786270	4856298	a	219106	29871	b	0,00
X1108	6122537	804092	a	260722	121830	b	0,00
X1110	616427	209136	a	10265	5150	b	0,01
X1113	73703	14698	a	5937	1336	b	0,00
X1114	2167128	245010	a	381985	62662	b	0,00
X1115	465057	77993	a	11298	2071	b	0,00
X1117	82417	13157	b	94811	25401	a	0,67
X1118	62820	15900	b	187493	63450	b	0,06
X1119	2598153	425964	a	9649	3643	a	0,00
X1120	0	0	b	7926	2737	b	0,01
X1121	44151	23833	a	17197	5811	b	0,28
X1122	570929	70937	b	5798849	1030164	b	0,00
X1123	15917947	1458394	a	81853	32032	b	0,00
X1124	40752822	1798796	a	433835	92881	b	0,00
X1126	29419962	3331474	a	19520	7848	b	0,00
X1128	209385	32050	a	14653	5361	b	0,00
X1131	6951729	1180929	a	69184	8963	b	0,00
X1132	346165	41572	a	67318	6622	b	0,00
X1134	18651789	2446924	a	1681362	364119	a	0,00
X1136	1370608	170057	a	18108	1832	b	0,00
X1137	1716674	260861	a	234023	42596	b	0,00
X1142	1200005	201313	a	2076	765	b	0,00
X1143	1033280	329363	b	3584736	972946	a	0,02
X1145	156350	40768	a	6557	1085	b	0,00
X1146	417735	104048	a	16193	11573	b	0,00
X1147	16377	14517	a	4106	741	b	0,40
X1148	696058	220995	a	67834	14887	b	0,01
X1149	67047464	2014487	a	24943089	2272054	a	0,00
X1150	7068633	362685	a	2273383	275253	b	0,00
X1152	7898508	387773	a	2452971	299017	a	0,00
X1153	60867	11681	a	19478	2901	b	0,00
X1156	2145149	502935	a	53631	12057	b	0,00
X1157	1029932	157503	a	75766	16464	a	0,00
X1160	9806	5307	b	459947	291920	b	0,13
X1161	1967184	148474	a	85340	16959	b	0,00
X1162	3748644	875784	a	16324	5162	b	0,00
X1166	1940650	358184	a	80548	31346	a	0,00
X1167	12789	1727	a	7870	1127	b	0,02
X1169	2021979	785504	a	287	200	b	0,01
X1170	42276010	5775802	a	38542876	4098950	a	0,60

X1171	81963	26017	b	392453	121959	a	0,02
X1175	172414	43258	b	42818789	1942143	a	0,00
X1176	10634677	1126690	a	9689850	1524208	a	0,62
X1177	9613724	1992589	a	7375453	1537989	a	0,38
X1181	736977	139110	a	33476	4621	b	0,00
X1182	2240841	923988	a	9628	7354	b	0,02
X1183	2496331	241900	a	40540	17541	b	0,00
X1184	57609	10158	b	76705	8640	b	0,16
X1185	17805630	2146138	a	80529	10021	b	0,00
X1187	1797865	254999	a	18300	4194	b	0,00
X1188	1549330	376384	a	1215	755	b	0,00
X1191	9313220	502084	b	10455017	424222	a	0,09
X1193	7656874	1174235	a	8352	3214	b	0,00
X1194	1469183	681462	a	46071	20831	b	0,04
X1197	4912277	521910	a	5493	1996	b	0,00
X1201	1049474	389513	a	0	0	b	0,01
X1203	2283153	294274	a	44976	9206	b	0,00
X1208	4461208	889429	a	295304	83031	a	0,00
X1209	226598	47509	a	15826	6145	b	0,00
X1210	1768146	491185	a	578426	115774	b	0,02
X1211	2295756	912799	a	2402	1166	b	0,01
X1212	10452739	1624993	a	0	0	b	0,00
X1213	52279613	7388878	a	4005738	1098204	a	0,00
X1214	875924	288341	a	181597	62689	a	0,02
X1217	123457	52386	b	263263	91534	a	0,19
X1218	675	383	b	10348	3014	b	0,00
X1220	48418	27336	b	55643	17425	b	0,82
X1223	2410704	719776	a	3761	1915	b	0,00
X1228	23153818	5236455	a	0	0	b	0,00
X1231	625490	107605	b	818062	291131	a	0,54
X1233	708428	132755	a	97268	62732	b	0,00
X1238	2897174	522502	a	1872288	471088	b	0,15
X1239	7865169	1023084	a	631916	229496	b	0,00
X1241	2057870	564657	a	0	0	b	0,00
X1242	1955945	344228	a	3134	1284	b	0,00
X1243	2005184	266394	a	142494	57671	b	0,00
X1246	2529068	602478	a	0	0	b	0,00
X1252	10573	2188	b	28645	9451	a	0,07
X1255	11909	9204	a	2092	606	b	0,29
X1260	550691	228152	b	1375104	706107	b	0,27
X1261	20447006	1569357	a	5314	2659	b	0,00

X1266	9244414	1640250	a	0	0	b	0,00
X1274	726285	250935	b	12412502	3098716	a	0,00
X1277	101884	23356	a	76497	19531	a	0,41
X1278	32476	11878	b	1028441	432205	b	0,02
X1279	7910388	2058602	a	0	0	b	0,00
X1281	87695	35643	a	40351	12207	b	0,21
X1282	1034851	189099	a	70248	32074	a	0,00
X1283	0	0	b	5478	1891	b	0,01
X1284	75019	19973	a	3492	2450	b	0,00
X1286	470816	132543	a	0	0	b	0,00
X1289	719273	219875	b	1978867	352260	b	0,00
X1290	2856671	879343	a	16649	3370	b	0,00
X1291	31973302	2412517	a	12692389	2422861	a	0,00
X1292	3561161	629882	a	735081	346882	b	0,00
X1295	1020742	167509	a	237631	109473	b	0,00
X1296	8812819	940938	a	6075519	1226683	a	0,08
X1297	2392	598	b	12632	6261	b	0,11
X1299	824458	410005	a	6671	930	b	0,05
X1300	163462173	13054452	a	1098452	603615	b	0,00
X1302	59832022	15475636	a	0	0	b	0,00
X1303	518543	130932	a	38027	10571	b	0,00
X1304	1343401	265118	b	3156226	681292	b	0,02
X1305	31924262	2791753	a	42540	19879	b	0,00
X1308	22964642	5299789	a	0	0	b	0,00
X1309	3032112	454763	a	1438	716	b	0,00
X1311	724420	124447	b	897585	266675	b	0,56
X1317	2880301	744745	a	1232	347	b	0,00
X1319	47726	17704	b	47817	17790	b	1,00
X1321	987706	184108	a	117955	42655	b	0,00
X1327	235908	68550	a	8734	3371	b	0,00
X1328	111599	47210	a	89076	27913	b	0,68
X1329	37256572	14456994	b	56729116	16192478	a	0,37
X1330	706964	232469	a	57722	12442	b	0,01
X1334	577181	200839	a	0	0	b	0,01
X1335	3707677	1446071	b	5459413	1680718	a	0,43
X1338	3744456	524943	a	2448135	452567	a	0,07
X1339	2026915	1878596	a	188705	52517	a	0,33
X1340	58374	15508	b	138243	58337	b	0,19
X1343	99624355	3778172	a	1262464	253819	a	0,00
X1345	415800	81866	b	1104577	583547	a	0,25
X1350	1003046	177777	a	33415	24728	b	0,00

X1351	10560806	615623	a	102799	23085	b	0,00
X1354	1654166	201609	a	77799	18250	b	0,00
X1355	396984	137952	b	8180433	1950813	b	0,00
X1358	106100285	9511431	a	35481	9881	b	0,00
X1359	973611063	71175853	a	4844769	1433261	a	0,00
X1360	205114	56395	b	919918	543803	a	0,20
X1361	459698	87709	b	774377	287556	b	0,30
X1363	11249730	1584652	a	0	0	b	0,00
X1366	0	0	b	1239544	375495	a	0,00
X1370	10665	2194	a	0	0	b	0,00
X1372	7726913	662515	a	592900	214050	a	0,00
X1374	8008	3548	b	34690	17147	a	0,13
X1375	5034	1566	a	1538	594	b	0,04
X1376	4207113	637841	a	141089	57744	a	0,00
X1377	106364	61289	a	16091	10943	b	0,15
X1380	4726545	694250	a	0	0	b	0,00
X1385	7653029	280735	b	8395283	200967	b	0,04
X1393	26496840	3233039	a	2896	1627	b	0,00
X1394	2340741	233012	a	17182	5921	b	0,00
X1400	4140110	519939	a	50768	17569	b	0,00
X1403	139276	70782	a	90643	38450	b	0,55
X1405	939530	303526	a	6164	2257	b	0,00
X1407	215262	41595	a	1062	717	b	0,00
X1408	30860937	2929446	a	182396	85154	b	0,00
X1409	147409986	10390910	a	106390	32860	b	0,00
X1411	331521	97228	a	297061	109652	b	0,81
X1412	1082767	598940	a	253526	88407	a	0,18
X1415	135527	37012	b	5280780	3451457	a	0,14
X1416	20428	4776	b	1259219	1080893	a	0,26
X1417	984638	250202	a	163777	41922	b	0,00
X1422	2761794	1249238	a	233406	65728	a	0,05
X1423	15387	10163	b	1678492	508485	a	0,00
X1424	157541	65668	b	373347	249160	b	0,41
X1425	6230459	676391	a	398795	86203	b	0,00
X1426	10465380	2525297	a	0	0	b	0,00
X1427	1714183	430481	a	326307	96113	a	0,00
X1428	2958	1468	b	3559	1099	a	0,74
X1431	0	0	b	143657	47692	a	0,00
X1433	966	493	b	162752	68055	b	0,02
X1434	52782603	2875319	a	1018369	176709	a	0,00
X1435	725	499	b	139272	58985	b	0,02

X1437	39442260	4441703	a	1661652	396755	a	0,00
X1438	7136	3211	a	1631	416	b	0,09
X1439	226232	134647	a	190202	88550	b	0,82
X1440	5345329	419806	a	109423	18779	b	0,00
X1442	3548266	395546	a	217502	46620	a	0,00
X1443	7110	2314	b	108344	49341	a	0,04
X1445	9634	6542	a	2487	1013	b	0,28
X1455	2364585	477263	a	368357	139981	a	0,00
X1457	219361	48831	a	29176	12974	b	0,00
X1462	418495	46486	a	13041	4853	b	0,00
X1467	18709	4767	a	0	0	b	0,00
X1469	7129548	1298889	a	59747	25824	b	0,00
X1470	6533354	1522784	a	0	0	b	0,00
X1477	1247719	1039119	a	132453	49997	b	0,29
X1478	890402	196395	b	3320555	1548403	a	0,12
X1479	318067	102764	a	45499	12260	b	0,01
X1480	64990	50248	a	5672	2302	b	0,24
X1484	9756435	1210196	a	0	0	b	0,00
X1486	7639	3565	a	6056	3691	b	0,76
X1487	3637979	881243	a	747846	275392	a	0,00
X1489	534565	161012	a	100000	45863	b	0,01
X1491	765894	337933	b	2801959	881463	b	0,04
X1495	5567586	966673	a	1611	1067	b	0,00
X1496	12176663	2189177	a	489441	125210	a	0,00
X1497	45221	27757	b	62170	32055	b	0,69
X1498	958670	213801	b	1258584	351977	b	0,47
X1499	9776129	2089584	a	1910341	688150	a	0,00
X1500	1136302	267604	a	32367	10388	b	0,00
X1501	104744	31847	b	211604	67364	a	0,16
X1503	8463	2473	a	7161	3136	b	0,75
X1509	9148099	792297	a	71532	18221	a	0,00
X1514	34012	8178	b	208861	99854	b	0,09
X1524	24027708	2307201	a	59748	23198	b	0,00
X1526	210186100	26836127	a	487574	118803	b	0,00
X1527	314176157	33807016	a	2962961	880453	b	0,00
X1529	19974401	2452655	a	503101	133783	b	0,00
X1530	3581451	381270	a	2596	1502	b	0,00
X1534	48437	21189	b	70315	34923	b	0,59
X1535	36076180	4386275	a	15366	6661	b	0,00
X1536	51175141	5942771	a	229325	84104	b	0,00
X1537	1219746	158012	a	20953	8138	b	0,00

X1540	11868000	1040228	a	27648	8498	b	0,00
X1541	6972096	513037	a	174062	42138	b	0,00
X1543	8829507	878928	a	9536	3320	b	0,00
X1544	2629825	1557873	a	215168	68324	b	0,13
X1547	9568084	797280	a	72567	17706	b	0,00
X1548	2093877	197531	a	11874	3853	b	0,00
X1550	221638951	13562855	a	3873596	1048421	b	0,00
X1551	14082223	3438230	a	82969	25365	b	0,00
X1554	737895	216266	a	20537	7407	b	0,00
X1555	131273	34157	b	1424207	322131	b	0,00
X1558	93927643	3516850	a	26618567	3777449	a	0,00
X1560	2279215	672923	a	2922	1267	b	0,00
X1561	59875776	8736280	a	430113	240813	a	0,00
X1562	141664	31286	a	17999	5658	b	0,00
X1563	5524434	1946537	b	62482683	13510922	a	0,00
X1564	3174	1728	b	97519	43170	b	0,03
X1565	28009173	1673818	a	5381415	997010	b	0,00
X1567	8795124	1542175	a	26852	19578	b	0,00
X1568	30524	22608	b	2430790	670782	b	0,00
X1569	24499767	1116096	a	6499499	1003626	b	0,00
X1570	15446675	1422817	a	7061	2081	b	0,00
X1573	9072036	603667	a	86502	18126	a	0,00
X1576	5990	2960	b	786893	418659	b	0,07
X1578	2974095	248565	a	136466	43382	a	0,00
X1583	16597	5551	b	62199	39745	b	0,26
X1585	871301	162659	a	6371	2211	b	0,00
X1590	14041926	2745864	a	65599	22158	a	0,00
X1592	19420	5435	b	28980	8214	a	0,34
X1597	23270	5377	a	2024	1139	b	0,00
X1601	124622	20571	a	2675	1085	b	0,00
X1602	102716	26044	b	192932	85998	b	0,32
X1603	11044786	1971827	a	0	0	b	0,00
X1604	1705673	180454	a	45209	8099	b	0,00
X1605	186778	42711	a	32475	4453	b	0,00
X1606	2569156197	102201555	a	85193820	13551760	a	0,00
X1607	2111111	346015	a	29122	9409	b	0,00
X1610	90947956	3765104	a	380607	125446	b	0,00
X1613	453508161	17884386	a	12478296	2332161	b	0,00
X1615	40978388	2162188	a	190802	39640	b	0,00
X1621	51893716	2232592	a	170955	57216	b	0,00
X1622	18909193	1667019	a	26063	11606	b	0,00

X1625	800305	361846	a	21688	9249	b	0,04
X1627	4794877	343292	a	9818	4179	b	0,00
X1631	1084598	146519	b	19164291	924012	a	0,00
X1633	787802	266625	a	82653	23776	b	0,01
X1635	2462162	221831	a	53179	17821	b	0,00
X1636	1931819	288088	a	100787	35706	b	0,00
X1638	251838	117699	a	77892	54214	b	0,18
X1639	3819964	628972	a	4728	2190	b	0,00
X1640	171073	51381	a	4038	2473	b	0,00
X1641	20609	6492	a	6936	3704	b	0,07
X1647	5209062	722665	a	5332	2574	b	0,00
X1648	4568879	485911	a	45432	16734	b	0,00
X1649	5590501	1256218	a	0	0	b	0,00
X1653	3408032	407461	a	19230	7132	b	0,00
X1656	1795257	715570	a	2866	2144	b	0,02
X1659	369408221	26615601	a	1123391	304347	b	0,00
X1660	48052397	4236907	a	140395	44648	b	0,00
X1661	40240005	3360289	a	293601	128161	b	0,00
X1662	5904808	1334733	a	69633	25502	b	0,00
X1663	811759	118863	a	13852	4337	b	0,00
X1665	20280	4086	a	0	0	b	0,00
X1668	62593254	4608134	a	96611	25287	b	0,00
X1669	6122676	861146	a	2347	1310	b	0,00
X1670	4789135	531741	a	29543	20359	b	0,00
X1671	524100	209063	a	8424	3903	b	0,02
X1675	10841	7029	a	409	296	b	0,14
X1677	34349019	6898582	a	22063480	6835550	b	0,21
X1678	23781338	1021840	a	173892	44332	b	0,00
X1683	46066335	4015560	a	49264	14608	b	0,00
X1684	10569815	1870270	a	0	0	b	0,00
X1685	4503012	1010289	a	3321784	1063675	b	0,42
X1686	82575200	11053998	a	17534315	2673311	a	0,00
X1687	13343047	1349706	a	66752	43764	a	0,00
X1688	30357	5369	a	0	0	b	0,00
X1690	32321175	2789390	a	37350	10406	b	0,00
X1692	88982	25333	a	63204	23631	b	0,46
X1693	7415936	1758302	a	0	0	b	0,00
X1694	4847838	358288	a	1109025	393353	b	0,00
X1697	2533775	1209316	a	25652	10051	b	0,04
X1700	2095563	326604	a	231303	114307	a	0,00
X1701	10115	6400	a	3091	1587	b	0,29

X1707	904069	191211	a	378959	107862	a	0,02
X1708	8299	2439	b	18397390	1661499	a	0,00
X1711	50526	10788	a	4251	1826	b	0,00
X1713	3079513	317303	a	49823	12322	b	0,00
X1715	8003464	1675465	b	24230703	2638315	a	0,00
X1716	2623535	617256	a	51450	14006	a	0,00
X1717	6786	5776	a	5336	4223	b	0,84
X1718	134032	38138	a	13181	9173	b	0,00
X1722	2908234	520620	b	6549092	841934	a	0,00
X1726	6207	2105	b	12599	5613	b	0,29
X1729	573488	81633	a	2474	715	b	0,00
X1736	19132	5869	a	9732	5495	b	0,25
X1738	5032042	745716	a	316077	71149	b	0,00
X1741	16859662	2172672	a	0	0	b	0,00
X1742	20265270	3040983	a	33197	10810	b	0,00
X1743	529466	83283	a	83691	16098	b	0,00
X1747	107368	21512	a	2295	542	b	0,00
X1750	34033	15993	b	135709	49072	b	0,05
X1751	4797165	364766	b	5673436	314168	b	0,07
X1755	10912108	1140264	a	820521	365876	b	0,00
X1761	5104936	805088	a	45758	19256	b	0,00
X1765	2001259	541296	a	0	0	b	0,00
X1766	214833	96973	b	558931	177953	a	0,09
X1768	42314	14613	a	0	0	b	0,01
X1770	205534	73958	a	71143	52600	b	0,14
X1771	84588	14006	a	1797	658	b	0,00
X1774	22863	12683	a	5237	3881	b	0,19
X1777	48016284	3777830	a	579398	109523	a	0,00
X1778	24244	13840	a	7184	3993	b	0,24
X1779	36704	8643	a	7601	1666	b	0,00
X1780	7931900	1124473	a	0	0	b	0,00
X1785	363692	73287	a	1264	633	b	0,00
X1786	6866650	663993	a	78920	15970	b	0,00
X1788	590594	87960	a	4489	2577	b	0,00
X1789	125462	16180	a	25346	12011	b	0,00
X1790	486437	65094	a	147430	58192	a	0,00
X1792	50264	11932	a	17036	8877	b	0,03
X1795	56645	16181	b	145267	47884	a	0,08
X1799	3519	1388	b	3730	3145	b	0,95
X1804	269404	83913	b	647055	369581	b	0,32
X1806	3075018	530126	b	4031467	962970	b	0,39

X1807	4214912	314739	a	55279	14430	b	0,00
X1810	14058659	1975240	a	271768	91947	b	0,00
X1811	9685964	762303	a	269417	49996	a	0,00
X1813	7399	3996	a	337	266	b	0,08
X1814	9838977	2376357	a	0	0	b	0,00
X1816	1763868	147230	a	33073	8245	b	0,00
X1819	3733187	469724	a	55305	17321	b	0,00
X1821	142143	29110	b	224095	55944	b	0,20
X1822	122764695	8958576	a	1577830	382663	a	0,00
X1823	1029720	249913	a	2013	883	b	0,00
X1824	484251	113398	a	3712	2332	b	0,00
X1826	6200752	988298	a	14013	5470	a	0,00
X1833	7602	1640	a	2707	646	b	0,01
X1837	165834	35243	a	52292	24269	b	0,01
X1839	246261	31527	a	16213	7173	b	0,00
X1843	424036	76803	a	1281	610	b	0,00
X1846	6000185	1078444	a	9271	5370	b	0,00
X1847	813988	244029	a	3695	1699	b	0,00
X1851	7644295	772841	a	969043	213245	b	0,00
X1853	50189132	5762301	a	213323	80116	b	0,00
X1854	660071	280238	a	14437	8087	b	0,02
X1856	1375824	180946	a	171649	40483	b	0,00
X1862	3097349	670842	a	0	0	b	0,00
X1866	1284822	536078	a	0	0	b	0,02
X1867	3172986	681567	a	1016	406	b	0,00
X1871	45841	21416	a	1171	1027	b	0,04
X1872	745370	242107	b	1247832	536330	b	0,40
X1875	10780572	1211137	a	23976	8318	b	0,00
X1878	8245404	623675	a	51472	12206	a	0,00
X1881	11149	3995	a	0	0	b	0,01
X1886	5831174	503447	a	261199	79587	a	0,00
X1890	8732	3774	a	563	372	b	0,04
X1894	3032100	272158	a	83240	25202	b	0,00
X1895	265093	44080	a	19607	10684	a	0,00
X1897	0	0	b	14333	4529	a	0,00
X1898	0	0	b	9204	2000	a	0,00
X1900	0	0	b	1738	555	b	0,00
X1901	21797	7540	a	6293	1297	b	0,05
X1902	1338594	404737	a	21383	7313	b	0,00
X1906	276717	76569	a	1072	292	b	0,00
X1918	737003	193033	a	8894	4269	b	0,00

X1921	51526	30617	a	4226	2569	b	0,13
X1922	745583	100602	a	18064	7500	b	0,00
X1923	57730122	5446148	a	25424	16006	b	0,00
X1924	9832444	1362825	a	31109	13668	a	0,00
X1925	16575	2558	b	323701	180185	b	0,09
X1927	11746505	1281648	a	49835	19122	b	0,00
X1930	12388290	2157264	a	9903	3781	b	0,00
X1932	19197	3726	a	0	0	b	0,00
X1933	24565569	4767608	a	278195	141361	b	0,00
X1934	47924717	4659128	a	1240451	324088	a	0,00
X1937	286516	36786	a	25827	6882	b	0,00
X1938	4534679	1053032	a	41647	28621	b	0,00
X1939	10097730	1075352	a	182281	58436	b	0,00
X1943	21749188	2682875	a	32554	12693	b	0,00
X1951	4591811	647987	a	2245	1736	b	0,00
X1953	3524988	977438	a	40451	15544	b	0,00
X1955	59118250	5279821	a	1062723	231312	b	0,00
X1956	7683253	1506755	a	0	0	b	0,00
X1958	22204625	1540324	a	93019	24670	b	0,00
X1959	2107474	1146852	a	1171	1104	b	0,07
X1960	45421	11268	b	480665	89490	b	0,00
X1961	13144046	1216539	a	253442	54414	b	0,00
X1963	3862655	407089	a	18061	5573	b	0,00
X1970	2710106	562748	a	4962	2202	b	0,00
X1974	11281	2980	b	31208	11941	b	0,11
X1978	258216	36984	a	19991	9551	b	0,00
X1987	4171198	916837	a	0	0	b	0,00
X1991	662020	169226	b	851405	285321	b	0,57
X1992	76441337	3925367	a	966254	206982	b	0,00
X1993	9845361	976977	a	56349	18467	a	0,00
X1995	6647	2455	b	2234178	1811416	a	0,22
X1996	3209	1442	b	3973799	1680275	b	0,02
X1997	24130346	7642821	a	5853	4013	a	0,00
X2002	2496	1777	b	118196	25740	b	0,00
X2003	6667765	1571591	a	1038	756	b	0,00
X2005	83565002	10746637	a	14000	8705	b	0,00
X2006	2727212	440932	a	34230	10083	b	0,00
X2007	638110	71843	a	5422	1798	a	0,00
X2010	902	291	b	200574	199360	a	0,32
X2011	38323	17601	b	2339058	765032	a	0,00
X2012	936968	536959	a	88	62	b	0,09

X2017	2173	747	b	404957	135464	b	0,00
X2018	1140844	228777	a	8549	6655	b	0,00
X2021	16391535	2306599	a	246586	70357	b	0,00
X2022	39680243	2910766	a	329591	85065	b	0,00
X2027	8832583	2608028	a	92713	47676	a	0,00
X2029	73476	15542	a	15559	4949	b	0,00
X2030	445081	108631	a	10410	3856	b	0,00
X2031	127396	51924	b	184849	78832	b	0,55
X2033	6826338	773928	a	19590	11212	b	0,00
X2034	12594080	3705993	a	21046	16745	b	0,00
X2036	1535308	487593	a	9318	8433	b	0,00
X2040	14746378	5327722	a	9965	5962	b	0,01
X2042	1909181	718403	b	2692885	1179859	a	0,57
X2046	403951	162743	b	637568	276964	b	0,47
X2047	28840557	2317858	a	253865	90133	b	0,00
X2049	4584867	525413	a	64605	20497	b	0,00
X2052	1406955	333806	a	20388	9738	b	0,00
X2053	16517103	377982	a	2064018	534261	a	0,00
X2056	413233	38778	a	5800	2460	b	0,00
X2059	13972888	1930093	a	26860	17437	b	0,00
X2061	7267943	1227957	a	100824	30736	b	0,00
X2062	2925492	415940	a	9541	5028	b	0,00
X2063	2238925	201086	a	1257	546	b	0,00
X2064	5892753	914016	a	0	0	b	0,00
X2066	209332	146549	a	6516	4464	b	0,17
X2070	43626	14100	a	212	130	b	0,00
X2071	19772	11497	a	0	0	b	0,09
X2072	3450	1638	a	243	181	b	0,06
X2076	100451037	3768128	a	1937871	446483	b	0,00
X2080	20968544	866841	a	210556	52402	b	0,00
X2082	182981415	11962495	a	3343690	665233	a	0,00
X2084	843069	108955	a	8836	3652	b	0,00
X2087	41054	15088	a	2139	1216	b	0,01
X2089	40693544	2702640	a	582936	129604	b	0,00
X2090	4639691	397262	a	51061	13253	b	0,00
X2093	825021	131084	a	7282	3790	b	0,00
X2094	11801513	1798548	a	7830951	1605413	a	0,10
X2095	442950	61918	a	22075	9885	b	0,00
X2097	118738410	8970021	a	890633	270880	b	0,00
X2099	3504563	464561	a	45150	13923	b	0,00
X2101	1103576	195050	a	862365	197468	a	0,39

X2102	54907	13314	a	0	0	b	0,00
X2103	4906	993	b	95145	31895	b	0,01
X2104	22399647	2079933	a	76241	27292	b	0,00
X2107	3352984	294342	a	41312	12191	b	0,00
X2108	56009	12482	a	16314	9581	b	0,01
X2112	3150029	226440	b	3209467	286124	a	0,87
X2114	469403	126341	a	13072	4175	b	0,00
X2123	6176214	1179806	a	52030	14858	b	0,00
X2125	588081	157705	a	2289	1025	b	0,00
X2128	3093212	509693	a	7869	4261	b	0,00
X2132	7494639	1900620	a	0	0	b	0,00
X2135	61691508	10110407	a	254170	94886	b	0,00
X2138	3594812	489783	a	15052	8301	b	0,00
X2140	15627237	1446223	a	189175	45914	b	0,00
X2141	5387029	666153	a	81551	42292	b	0,00
X2143	1602261	148555	a	29938	9685	b	0,00
X2145	71448	23307	a	5342	2410	b	0,01
X2149	1482221	193920	a	7067	2134	b	0,00
X2150	837343	142966	a	3784	2941	b	0,00
X2151	53478351	4016252	a	399510	87479	a	0,00
X2153	360482	53370	a	245574	76440	b	0,22
X2155	12140588	950018	a	76846	18697	a	0,00
X2156	5745	2057	a	1199	788	b	0,04
X2162	25629	6759	b	42562	14967	b	0,31
X2163	69186	12650	a	38038	21794	a	0,22
X2167	5624	2076	b	12116	4020	b	0,16
X2169	17029	3351	a	0	0	b	0,00
X2170	3051	808	a	0	0	b	0,00
X2173	956603	181603	a	8112	2326	b	0,00
X2174	962042	128534	a	20089	6681	a	0,00
X2178	4650	1759	b	28045	12181	b	0,06
X2180	237499	26241	a	1367	644	a	0,00
X2182	0	0	b	4729	1901	b	0,02
X2186	63200	46237	a	404	256	b	0,18
X2187	3462296	495712	a	5356	1964	b	0,00
X2189	20522	13364	b	317498	143298	b	0,04
X2194	23143453	1538993	a	2582136	554410	b	0,00
X2199	7798885	1011707	a	914680	204276	b	0,00
X2200	42337427	7840158	a	379754	149883	b	0,00
X2203	7470801	760817	a	953423	203148	a	0,00
X2204	3227	1749	b	14317	9971	b	0,28

X2211	1582309	169582	a	911	587	b	0,00
X2216	11228465	1589171	a	0	0	b	0,00
X2218	948506	195920	a	4906	2569	b	0,00
X2220	4433462	734232	a	0	0	b	0,00
X2221	1963021	410290	a	53662	16084	b	0,00
X2227	450083	95677	a	8139	2735	b	0,00
X2230	484556	134424	a	21568	8303	b	0,00
X2233	6411069	449503	a	34632	9535	b	0,00
X2234	91935	35182	a	3308	1636	a	0,01
X2237	1065	559	b	2709968	865502	a	0,00
X2240	0	0	b	509170	140764	b	0,00
X2242	1670876	432690	a	1547	948	b	0,00
X2243	437692	105577	a	65596	27907	b	0,00
X2251	1140338	120496	b	6356437	744011	b	0,00
X2254	26684862	2184362	a	77067	20489	b	0,00
X2257	1118990	389250	a	0	0	b	0,01
X2258	5332272	702239	a	10816	3570	b	0,00
X2261	19125392	1666597	a	78584	27972	b	0,00
X2262	15309533	5201287	a	162891	80066	a	0,01
X2264	109492	24002	a	879	718	b	0,00
X2267	4035266	363316	a	15217	6297	b	0,00
X2269	2452027	1055203	a	16089	6925	b	0,02
X2272	2009197	336204	a	3366	939	b	0,00
X2273	1648466	226565	a	3711	1858	b	0,00
X2274	24206445	3007291	a	28966	13555	b	0,00
X2276	1248666	446190	a	29155	13029	b	0,01
X2278	5074772	744556	a	25515	8284	b	0,00
X2279	67982	18389	a	278	206	b	0,00
X2280	146749	19038	a	0	0	b	0,00
X2281	0	0	b	33854	12718	b	0,01
X2282	246174	34335	a	10017	3629	b	0,00
X2284	10083	3793	a	5607	3909	b	0,41
X2290	7288912	1194112	a	0	0	b	0,00
X2299	6741641	1308686	a	0	0	b	0,00
X2300	11560264	1400069	a	50639	12351	a	0,00
X2301	11643	2649	b	6432380	511608	a	0,00
X2304	1631780	252679	a	13256	7421	b	0,00
X2306	2855486	441393	a	8001	2994	b	0,00
X2310	122437	25877	a	40399	19001	b	0,01
X2311	8632386	705275	a	246274	46830	b	0,00
X2312	868493	295043	a	2401	1916	b	0,00

X2313	2299135	253804	a	3037	1110	b	0,00
X2315	8659	2524	a	5752	3429	b	0,50
X2316	2237782	196456	a	54973	12913	a	0,00
X2319	45989	12792	a	357	259	b	0,00
X2320	1923246	138017	a	46954	9957	b	0,00
X2322	5353524	786622	a	49946	15659	b	0,00
X2325	1142967	170882	a	6954	3128	b	0,00
X2326	3541575	1729141	a	1190	956	b	0,05
X2327	4803537	513616	a	67411	16786	b	0,00
X2329	5760599	803706	a	90959	26544	b	0,00
X2330	93237	31842	a	0	0	b	0,00
X2332	894896	168861	a	13203	6112	b	0,00
X2338	261828	18940	a	1431	823	b	0,00
X2340	3387597	437038	a	6022	2269	b	0,00
X2348	13650227	2074736	a	0	0	b	0,00
X2349	18380356	4705760	a	0	0	b	0,00
X2355	10489832	629877	a	0	0	b	0,00
X2361	0	0	b	6039537	1050925	b	0,00
X2362	9063621	1114367	a	5445	2465	b	0,00
X2363	4551953	826328	a	0	0	b	0,00
X2366	29995	11637	a	1153	636	b	0,02
X2369	2308128	232850	a	8668	4770	b	0,00
X2372	11901915	1428424	a	35721	13368	b	0,00
X2374	9124579	1175221	a	18351	8583	b	0,00
X2378	2654536	304467	a	4663	2157	b	0,00
X2379	136060	24686	a	790	427	b	0,00
X2381	4261160	550836	a	12521	5105	b	0,00
X2382	10597439	1571811	a	31300	10369	b	0,00
X2386	265725	89280	b	6454447	1482904	a	0,00
X2388	1165906	120478	a	9536	2234	b	0,00
X2390	53862	23431	b	1522835	438740	a	0,00
X2393	4994	2315	b	171590	54785	b	0,00
X2402	9093105	3072609	a	0	0	b	0,00
X2403	1101497	399531	a	7541	4665	b	0,01
X2405	19981487	4424158	a	0	0	b	0,00
X2410	3569598	839164	a	0	0	b	0,00
X2412	18740555	4666301	a	0	0	b	0,00
X2417	103751694	8471951	a	649834	202203	b	0,00
X2419	7353806	811939	a	179451	54501	b	0,00
X2421	26481149	2303108	a	133385	47022	b	0,00
X2425	3964973	412460	a	13933	6006	a	0,00

X2428	9389	3857	a	1359	1198	b	0,05
X2432	4479423	1495544	a	86627	24904	b	0,00
X2435	2551162	696619	a	478604	258295	a	0,01
X2450	382646	66412	a	120668	52898	b	0,00
X2451	30891508	3644539	a	0	0	b	0,00
X2452	59730781	7726900	a	0	0	b	0,00
X2456	66053	17199	a	23939	12346	b	0,05
X2458	4960273	1109473	a	0	0	b	0,00
X2464	2541826	697987	a	24472	9681	b	0,00
X2466	2195080	465926	a	3878	1551	b	0,00
X2467	1130448	175584	a	11841	3186	b	0,00
X2470	200827	31373	a	5719	3398	b	0,00
X2472	11862879	968815	a	12299	6211	b	0,00
X2473	267898	28438	a	997	472	b	0,00
X2474	155349	20049	a	6829	4010	b	0,00
X2476	1167037	193736	a	5113	2243	b	0,00
X2478	47618204	2658199	a	33508	19476	b	0,00
X2479	14280074	1065951	a	4336	2749	b	0,00
X2483	8136413	2056279	a	206057	102331	a	0,00
X2485	168203	67134	b	221433	86433	b	0,63
X2488	5779314	2682739	a	5871	5235	b	0,04
X2492	36641	16121	b	58799	24502	b	0,45
X2497	20869236	1655844	a	593006	141522	b	0,00
X2498	6102228	549222	a	41754	11804	b	0,00
X2500	5841181	466661	a	156502	39987	b	0,00
X2501	1937957	307020	a	0	0	b	0,00
X2502	1454220	203063	a	6245	2495	b	0,00
X2503	3805851	312319	a	140382	31275	b	0,00
X2505	1022275	99291	a	31659	7742	a	0,00
X2507	27569	9603	a	0	0	b	0,01
X2508	943666	390488	a	216614	100064	b	0,08
X2509	6147711	629247	a	200961	43451	a	0,00
X2510	0	0	b	150344	66146	b	0,03
X2511	1741748	178257	a	44085	11237	b	0,00
X2512	1512050	189671	a	5187	2279	b	0,00
X2515	594397062	29345527	a	2058252	684533	b	0,00
X2522	26573045	1458080	a	16159	5312	b	0,00
X2526	53270646	11720219	a	0	0	b	0,00
X2528	13740280	3384298	a	0	0	b	0,00
X2529	2081615	353926	a	0	0	b	0,00
X2531	7308316	1923345	a	4882	4257	b	0,00

X2532	368441	66339	a	0	0	b	0,00
X2535	14147414	1749748	a	168844	48187	b	0,00
X2536	170121884	15887943	a	0	0	b	0,00
X2537	20860234	5573188	a	0	0	b	0,00
X2538	9018120	497510	a	51510	16750	b	0,00
X2539	7870582	1096407	a	75815	23228	b	0,00
X2540	1267752	201618	a	532	297	b	0,00
X2542	16849930	2218886	a	155293	42206	b	0,00
X2545	44903794	4477723	a	0	0	b	0,00
X2549	4227430	564305	a	23366	9730	b	0,00
X2550	3204614	915112	a	0	0	b	0,00
X2552	29662679	3837769	a	385036	101979	b	0,00
X2558	11941856	1150536	a	110093	26530	b	0,00
X2560	2524227	248742	a	21048	6011	a	0,00
X2564	11786	5031	b	90998	53679	b	0,15
X2565	2259803	665036	a	1290	656	b	0,00
X2567	19660691	2721526	a	0	0	b	0,00
X2571	3551	1128	b	12203	7152	b	0,24
X2573	729666386	16515825	a	44025670	8077526	b	0,00
X2576	215886495	4925327	a	12292397	2322025	a	0,00
X2577	4407502	814442	a	0	0	b	0,00
X2579	48081683	1168273	a	2049429	421416	b	0,00
X2580	24921419	2408740	a	386727	93815	b	0,00
X2582	5488979	250298	a	281022	64308	b	0,00
X2583	5985288	672542	a	83401	24352	b	0,00
X2593	341130	143762	b	475209	189545	b	0,58
X2597	5241605	877352	a	0	0	b	0,00
X2598	82540	36996	b	122684	50547	b	0,52
X2607	35834453	2326820	a	4148391	1573892	b	0,00
X2608	7833502	1198339	a	0	0	b	0,00
X2610	5091303	685138	a	3773	2204	b	0,00
X2611	9766337	763606	a	947195	432675	b	0,00
X2614	75251768	6878534	a	231666	70957	b	0,00
X2615	1807666	139720	a	172271	80895	b	0,00
X2617	20374201	2052503	a	34422	10798	b	0,00
X2618	2842181	1390910	a	0	0	b	0,05
X2619	6173732	281028	a	350760	92298	a	0,00
X2620	0	0	b	43915	24539	b	0,08
X2624	1274532	228243	a	0	0	b	0,00
X2629	46018	6416	a	0	0	b	0,00
X2645	0	0	b	1269962	439356	b	0,01

X2646	9597304	2251788	a	0	0	b	0,00
X2651	104120	28878	b	362783	140748	b	0,08
X2652	50458094	3342668	a	0	0	b	0,00
X2653	3380807	925786	a	0	0	b	0,00
X2655	6298818	587088	a	37130	10889	a	0,00
X2656	16619	6593	b	111230	44915	b	0,04
X2657	12588846	1074420	a	0	0	b	0,00
X2659	601	324	b	4592	2400	b	0,10
X2660	4709400	1276033	a	0	0	b	0,00
X2665	43631	19354	b	79702	35080	a	0,37
X2669	31006	22484	b	64312	40364	b	0,47
X2671	5179361	1922275	a	0	0	b	0,01
X2672	835893	89723	a	7382	4159	b	0,00
X2675	2039941	646037	a	1250	506	b	0,00
X2686	127555482	10581502	a	511789	144647	b	0,00
X2690	49172033	7071007	a	0	0	b	0,00
X2691	20589276	5334654	a	0	0	b	0,00
X2695	2713384	290913	a	10256	3575	b	0,00
X2698	61375400	6102717	a	0	0	b	0,00
X2704	635258	137100	a	309605	86723	b	0,05
X2709	824919	82957	a	24343	12222	b	0,00
X2710	9011926	867169	a	35907	11285	b	0,00
X2715	2724909	1331716	a	0	0	b	0,05
X2720	110626	44397	a	92977	45139	b	0,78
X2724	6935663	2263186	a	4816	2369	b	0,00
X2728	10563312	1629040	a	0	0	b	0,00
X2736	21347492	1897774	a	0	0	b	0,00
X2737	49269361	5131648	a	0	0	b	0,00
X2742	12363082	1526410	a	0	0	b	0,00
X2743	11148019	2659119	a	0	0	b	0,00
X2745	3043257	796012	a	0	0	b	0,00
X2750	12342662	1729075	a	0	0	b	0,00
X2751	4152177	291349	a	49393	14000	b	0,00
X2754	1033855	144536	a	5156	1706	a	0,00
X2757	0	0	b	128135	70874	a	0,08
X2763	387	272	b	65474	27706	b	0,02
X2767	18985507	4770202	a	0	0	b	0,00
X2772	6434401	758619	a	2249	1464	b	0,00
X2783	9132438	1030157	a	0	0	b	0,00
X2787	5477414	1185753	a	0	0	b	0,00
X2788	871644	68573	a	64190	35953	b	0,00

X2798	18865431	1950162	a	0	0	b	0,00
X2805	16130120	2218172	a	0	0	b	0,00
X2806	10238206	2647116	a	0	0	b	0,00
X2813	4410914	1811328	a	4250	3935	b	0,02
X2814	15733671	2508788	a	0	0	b	0,00
X2823	7656273	1145518	a	0	0	b	0,00
X2827	10278410	2437439	a	12518	5013	b	0,00
X2829	2448597	681601	a	0	0	b	0,00
X2831	13416194	3558564	a	1148	1060	b	0,00
X2834	3089493	802033	a	0	0	b	0,00
X2836	5463777	1647003	a	38282	28711	b	0,00
X2840	1558645	498050	a	11357	9923	b	0,00
X2853	358607167	14496400	a	1470342	576402	b	0,00
X2855	21876663	1072714	a	0	0	b	0,00
X2856	17556825	4111503	a	0	0	b	0,00
X2858	5898780	699911	a	25472	10199	b	0,00
X2861	23186972	1844781	a	0	0	b	0,00
X2863	4589856	722389	a	0	0	b	0,00
X2867	2901424	362040	a	10790	4711	b	0,00
X2872	310350	140645	b	833878	459515	a	0,28
X2878	138227	63375	b	258821	114437	b	0,36
X2883	13542534	1925391	a	0	0	b	0,00
X2891	3542165	993179	a	0	0	b	0,00
X2895	6624828	1500608	a	0	0	b	0,00
X2903	11631103	2517571	a	0	0	b	0,00
X2904	68020	14540	a	0	0	b	0,00
X2905	87801304	5094945	a	36381	19026	b	0,00
X2907	2323820	612403	a	0	0	b	0,00
X2908	33355792	1977612	a	11443	6507	b	0,00
X2911	8056037	894879	a	0	0	b	0,00
X2915	7353635	3556759	a	31150	16197	b	0,04
X2917	15890436	1556350	a	0	0	b	0,00
X2920	1043379	751184	a	7436	4205	b	0,17
X2923	1240288	574580	a	8065	4982	b	0,04
X2926	6293477	1833182	a	0	0	b	0,00
X2930	2867746	187919	a	16747	6930	b	0,00
X2937	113597021	43426096	a	632440	339854	b	0,01
X2941	40400535	15490144	a	209868	92873	b	0,01
X2942	9244483	3911818	a	5860	1942	b	0,02
X2943	7397013	2478888	a	0	0	b	0,00
X2945	1124110	546522	a	4215	3454	b	0,04

X2947	7014924	1143214	a	592514	165423	b	0,00
X2948	1561314	290398	a	195013	58121	a	0,00
X2953	42523	23756	a	33112	15237	b	0,74
X2960	22159180	3036900	a	24080	14859	b	0,00
X2962	1614306	250287	a	20922	6091	a	0,00

Table S4. Deconvoluted total intensities (mean ± S.D.) of all identified in leaf organ in different antibiotic treatment within the plants receiving control levels of water in leaf. The statistically

significant differences between treatments detected by Tukey's HSD post-hoc tests are indicated by bold letters (*P*< 0.05). Metabolites as in Fig. 1 caption, the X represent the unknown metabolites.

**Effect antibiotic in organ leaf**							
**Metabolites**	**Antibiotic**			**Control**			
	**Mean**	**S.D.**		**Mean**	**S.D.**		**F(p<0)**
Ala	158193	37948	b	590316	263844	a	0,00
Arg	86597	32766	b	121170	60235	a	0,17
Asn	9199597	4883067	a	8656529	3630604	b	0,65
Asp	2966695	726859	a	2701577	533287	b	0,93
Gln	128604	36765	b	210703	36140	a	0,76
Glu	2020792	566822	b	3255075	634066	a	0,13
Glup	18101598	5786630	a	11789524	2634393	b	0,17
His	14550	7977	b	50842	14684	a	0,29
HPro	4428123	2510473	a	8594	6740	b	0,06
Iso	27609397	5710435	b	41087151	8030726	a	0,05
Lys	87958	43970	a	70855	32204	b	0,21
Met	495343	237431	a	213355	47501	b	0,75
Phe	37909315	8759499	b	44730639	8996970	a	0,19
Pro	3802362	1641856	b	5303257	1306529	a	0,60
Ser	247746	46431	a	143382	32122	b	0,47
Thr	13433662	3073167	b	20350701	3197736	a	0,06
Try	33340088	12466228	b	34749212	8744557	a	0,14
Tyr	152234480	36007494	b	175348115	29904109	a	0,92
Val	14958045	3508979	a	14203281	3379351	b	0,62
Ad	16751853	5067275	b	33344316	15913846	a	0,88
AMP	413440	162799	a	111671	37776	b	0,39
Cy	13325	6539	b	85496	50873	a	0,05
Gua	274261	143239	b	2056587	1101493	a	0,23
Ur	528657	334737	a	474342	274164	b	0,17
Dis	3895228	839078	a	3297473	635968	b	0,90
Hex	8666964	1907903	b	11954959	3233042	a	0,57
Pen	1750368	405613	b	2988524	659638	a	0,43
Raf	8675	4164	b	21190	4908	a	0,15
Xyl	1829269	605546	b	3031969	694278	a	0,07
Cit	32112335	6203420	a	30372464	7471624	b	0,22
Lac	4582286	1387959	b	4910805	1331647	a	0,87
Mal	193722345	40110476	a	184935539	27632151	b	0,87
OxA	262197	248944	a	164059	109654	b	0,85
PyA	4162143	1398888	a	2796365	742890	b	0,70
ShA	2135670	520861	b	2692953	684619	a	0,36

AbA	698858	149166	b	1090778	199515	a	0,55
AsA	8667217	2105355	a	3554022	905123	b	0,15
CafA	4630852	1229257	a	4409889	853610	b	0,02
Cat	20850184	5459443	b	24466013	5068853	a	0,88
CGA	207500399	68271770	b	379263484	72552728	a	0,63
CGAp	142900392	32753436	b	173658549	27865317	a	0,10
Chr	153003	66971	a	5108	3152	b	0,48
CoA	860422	243431	b	1777321	418446	a	0,01
FeA	16381438	4530402	b	26207047	5341496	a	0,09
Fis	170018	101459	a	124348	53302	b	0,19
Hom	7105091	2945905	a	2018976	791928	b	0,67
Hom.1	1834475	558115	a	1516132	279582	b	0,06
Kae	47674	20483	b	116935	40321	a	0,59
Pin	499668	141519	b	1564677	475406	a	0,17
Pro.1	372659	97243	b	387614	143503	a	0,07
Que	2397547	600090	b	2936357	1092647	a	0,94
Rha	6205	3568	b	8613	2967	a	0,70
Sal	154128	58754	b	249213	62097	a	0,60
Sap	74507961	18991198	b	99097749	17120459	a	0,29
SiA	3601725	1246397	a	2292237	723620	b	0,34
SuA	28819037	5581681	a	20096626	5669848	b	0,34
Ani	832878	346918	a	577222	204862	b	0,29
Car	255369	99116	a	183226	52351	b	0,51
Cho	211034989	41909991	a	179875881	38393474	b	0,49
JaA	34617	9054	b	55122	16302	a	0,59
Log	1286180	341337	a	1262142	285697	b	0,32
VaA	968897	257716	b	1983329	866664	a	0,96
Nic	340939	138328	b	984029	332384	a	0,33
Rib	844793	173363	a	525848	106989	b	0,12
Sec	14596497	3452874	b	15702257	4644915	a	0,11
Toc	9155224	1846401	b	9540007	1960636	a	0,86
Vi.B1	1088225	361887	a	1014024	228789	b	0,89
Vi.B5	111459	43620	b	149677	30301	a	0,86
Vi.B6	694022	181475	a	354340	90459	b	0,46
Vit	31240	25830	a	8467	4125	b	0,08
X2186	31986	15012	b	68402	51369	a	0,00
X2853	199394888	35830962	a	193614199	34790264	b	0,00
X2522	13301443	3329064	b	14197488	2665919	a	0,00
X1541	5353938	852165	a	3583641	736403	b	0,00
X359	1108818	432652	a	586054	293738	b	0,00
X29	188844645	44790576	a	187515704	36981681	b	0,00

X2273	1184010	281350	a	831149	232814	b	0,00
X1308	5769156	3919340	b	8129538	2618323	a	0,00
X1153	41194	17026	b	42410	12378	a	0,00
X2140	6774184	2001892	b	9451214	1683569	a	0,00
X2027	642190	329221	b	3314914	1646010	a	0,00
X1385	4022375	925374	a	3965055	658544	b	0,00
X2474	89510	23002	b	127336	21039	a	0,00
X313	657658	204616	b	761344	258408	a	0,00
X632	33667916	8072788	a	12261092	3655898	b	0,00
X2920	4590884	2154946	a	62434	60202	b	0,01
X1958	10594650	2414963	a	7644892	1779754	b	0,01
X1688	41112	17863	a	21707	4556	b	0,01
X528	13684	6107	b	16215	7578	a	0,01
X2926	2798498	1187935	b	4289717	1662232	a	0,01
X2720	88	88	b	48177	23499	a	0,01
X2520	8286383	2156996	a	1106823	765545	b	0,01
X2417	60981735	15974989	a	52841376	11662034	b	0,01
X2269	834966	346516	b	1548520	1083824	a	0,01
X1951	1392233	546655	b	2282742	519059	a	0,01
X1110	543969	187123	b	573594	230774	a	0,01
X1231	194442	100036	b	276556	77112	a	0,01
X2151	21467713	5443069	b	23308471	5407166	a	0,01
X2503	2784389	911368	a	2438314	428923	b	0,01
X717	89976	30524	a	53969	18785	b	0,01
X638	3311309	1402599	a	1806484	664246	b	0,01
X547	1327576	871204	b	1653752	801066	a	0,01
X1576	614	454	b	6090	3272	a	0,01
X2814	5142869	1876215	b	9006871	2339072	a	0,01
X545	123106	37251	b	148948	39671	a	0,01
X1284	29624	14139	b	46088	15833	a	0,01
X167	9008883	4000772	b	25698165	11030781	a	0,01
X1991	460925	163793	a	446136	163736	b	0,01
X1409	37999755	12750556	b	78664038	14757695	a	0,01
X2338	123489	26666	b	163757	29029	a	0,01
X1477	120592	33425	b	1244681	1156649	a	0,01
X2836	4097449	1754314	a	1002873	393501	b	0,02
X1693	1769885	872396	b	2891053	1089273	a	0,02
X72	246376991	53143353	a	238546657	44000780	b	0,02
X5	27479	7263	b	48471	8546	a	0,02
X477	1514706	594548	a	983406	612011	b	0,02
X1213	22429976	6651260	b	29477957	7082133	a	0,02

X291	8552504	2583358	a	6717339	2013437	b	0,02
X622	377749	153327	a	283949	101513	b	0,02
X2921	58360	40306	b	315742	309173	a	0,02
X2282	153661	68392	b	159284	39246	a	0,02
X143	316304898	76348779	b	542980824	80960433	a	0,02
X217	2821721	795052	b	3173313	683843	a	0,02
X1946	936	396	a	3	3	b	0,02
X2895	2284947	1005587	b	4062598	1321050	a	0,02
X337	7846343	3579741	b	10958557	2991884	a	0,02
X20	2972186	765685	b	2994685	688223	a	0,02
X1636	784335	261229	b	1074777	271811	a	0,02
X779	2037318	461988	a	1988668	477597	b	0,02
X1685	5467398	2653378	a	3095189	1028318	b	0,02
X1166	1374862	346391	a	892870	202630	b	0,02
X800	11965910	2614307	a	6646691	1595517	b	0,02
X2615	761211	137704	b	862240	196861	a	0,02
X2293	1867550	929915	a	1141952	587161	b	0,02
X676	58695	56776	a	33894	22954	b	0,02
X356	15939117	4165983	a	10031300	2695961	b	0,02
X569	9875168	3073039	a	6126456	2763722	b	0,02
X1097	1327368	396986	a	821927	229096	b	0,02
X230	10846370	4583778	a	5091473	1840777	b	0,03
X1327	76837	34130	b	177109	69844	a	0,03
X1056	37828	13844	a	21934	6583	b	0,03
X2891	3337316	1112330	a	826973	361332	b	0,03
X1529	7504080	2628346	b	11352941	2214928	a	0,03
X1191	5357390	1230615	a	4129426	856068	b	0,03
X1603	10296002	2384190	a	4694715	1588257	b	0,03
X2532	107928	21600	b	202162	58029	a	0,03
X2450	148697	50982	b	189009	54841	a	0,03
X489	530846	149274	a	479140	105072	b	0,03
X1554	443205	227970	b	783523	296755	a	0,03
X1311	212980	93658	b	318870	91022	a	0,03
X1643	4931	4691	b	43743	26999	a	0,03
X1201	628234	423415	b	779159	227248	a	0,03
X2428	478	478	b	3374	1179	a	0,03
X2460	2880	1485	a	792	348	b	0,03
X467	105474	28684	a	56404	16187	b	0,03
X2515	358927118	68490143	a	249661431	58749763	b	0,03
X1188	301915	236449	b	1464832	404611	a	0,03
X1091	30983	6055	b	94655	34744	a	0,03

X2412	10735087	2714470	a	8505895	3879440	b	0,03
X1252	7036	2051	a	5839	1624	b	0,04
X1839	157904	22679	a	139258	33909	b	0,04
X2369	2807395	926127	a	1116991	230513	b	0,04
X2657	7377986	1338337	b	7675119	1405728	a	0,04
X1437	29540234	8263027	a	14016941	3852940	b	0,04
X2059	9391496	2218988	a	8118344	2166042	b	0,04
X35	118268	32801	a	70660	18749	b	0,04
X804	1836603	763310	a	1149380	583711	b	0,04
X2783	6971257	1670207	a	3350909	901366	b	0,04
X818	5654776	1617999	b	9416235	3222566	a	0,04
X615	9478533	2521713	b	11351322	2714044	a	0,04
X318	114448	114309	b	1752609	1140963	a	0,04
X1321	268781	96817	b	1287254	375606	a	0,04
X1351	5140135	1068416	b	5372391	1134899	a	0,04
X1350	333079	123311	b	995361	255770	a	0,04
X2128	1686764	546216	b	2300170	533849	a	0,04
X1044	5082787	1529086	b	9204800	1682464	a	0,04
X1470	6586525	1795424	a	2059240	781953	b	0,04
X263	30845826	8155438	a	22307297	5749539	b	0,05
X1033	86419	27217	a	75031	23037	b	0,05
X2785	505441	293906	a	30934	30934	b	0,05
X1604	1111301	348595	a	1100079	229819	b	0,05
X2432	1052910	670411	b	1572181	690114	a	0,05
X2372	5733484	2108357	b	5861270	996254	a	0,05

Table S5. Deconvoluted total intensities (mean ± S.D.) of all identified in leaf epiphytic in different antibiotic treatment within the plants receiving control levels of water in leaf. The statistically significant differences between treatments detected by Tukey's HSD post-hoc tests are indicated

by bold letters ($P < 0.05$). Metabolites as in Fig. 1 caption, the X represent the unknown metabolites.

Effect antibiotic in epiphytic leaf							
**Metabolites**	**Antibiotic**			**Control**			
	**Mean**	**D.S.**		**Mean**	**D.S.**		**Pr(>F)**
Ala	7955470	2627520	a	2041727	504033	b	0,08
Arg	3060576	1570441	a	1379732	475230	b	0,40
Asn	12965030	2084917	a	10447964	3683284	b	0,53
Gln	18309571	3598378	a	7108654	3112740	b	0,03
Glu	908580	160547	a	368394	76521	b	0,01
Glup	26294277	4134564	a	10392447	2543779	b	0,01
His	9508717	5618356	a	117113	47251	b	0,18
HPro	235828	175450	b	685358	601902	a	0,40
Iso	2495390	472758	a	1773154	623372	b	0,35
Lys	407594	198520	a	22324	9422	b	0,12
Met	2426742	1485212	a	499289	289592	b	0,30
Phe	3800704	850464	a	2780805	1413424	b	0,51
Pro	23571422	6983313	a	12101523	1443926	b	0,19
Ser	4876322	1453588	a	1555525	390984	b	0,07
Thr	3582386	626645	b	12492907	2486184	a	0,00
Try	619646	207709	b	19871539	3413903	a	0,00
Tyr	35096351	4201784	b	39519203	4463079	a	0,49
Val	33160550	3068327	b	41763979	10851159	a	0,37
Ad	957067	568661	a	174176	48575	b	0,27
Ade	198990	124669	a	38614	10621	b	0,30
AMP	342515	128256	b	576082	152235	a	0,25
Cy	40352	10782	a	33791	8764	b	0,66
Gua	47863	14256	a	35438	8634	b	0,51
Ur	43623	14105	a	14562	5432	b	0,11
Dis	5805750	794608	a	2648132	787245	b	0,01
Hex	9307000	874669	a	6086505	896169	b	0,02
Pen	392682	36282	b	536723	63977	a	0,04
Raf	18464023	3495702	a	7057704	3525185	b	0,03
Xyl	3329477	757660	b	4695042	1034307	a	0,28
AbA	32172	16020	b	42690	28854	a	0,73
AsA	121869	41544	b	160755	55098	a	0,57
CafA	19223	4510	a	10126	2297	b	0,13
Cit	3676553	757474	a	1810800	546621	b	0,08
Lac	14795588	2864936	b	19908681	3160720	a	0,25
Mal	23585497	3470339	a	23177683	7417319	b	0,96
OxA	50637	10483	a	39006	8085	b	0,43

PyA	69377	17053	b	105173	24981	a	0,23
ShA	513248	93016	a	469722	102027	b	0,76
SuA	7655080	1095928	b	12320184	2442501	a	0,06
Cat	2049628	331637	b	2186718	377105	a	0,79
CGA	10439992	1929960	a	9148274	1298924	b	0,62
CGAp	243725	62490	a	232830	96103	b	0,92
Chr	28625	25865	b	2815433	1148449	a	0,00
CoA	177515	55278	b	265731	96921	a	0,40
FeA	951378	292864	a	937374	233432	b	0,97
Hom	461660	257793	a	18882	9114	b	0,17
Hom.1	245730	86918	a	129680	32559	b	0,30
Pin	207982	48802	b	291233	80425	a	0,35
Pro.1	39673	6272	a	34703	3930	b	0,55
Sal	236501	50705	b	283363	57318	a	0,55
Sap	1028710	226835	a	824683	206439	b	0,53
SiA	5146	769	a	4769	1288	b	0,79
VaA	52718	6709	b	66995	14761	a	0,33
Ani	1106557	488546	a	1027694	351251	b	0,91
Car	9605926	1836092	b	11966062	2795429	a	0,46
Cho	48005199	8557512	a	38921478	2538576	b	0,40
JaA	5102	2190	a	4879	2195	b	0,95
Log	31704	7712	a	24105	7668	b	0,51
Rib	71450	10645	b	86854	15016	a	0,39
Sec	4085469	1183892	a	2631228	1596610	b	0,46
Toc	15599806	1727695	a	8175852	1740866	b	0,01
Vi.B1	43268	7898	b	83189	14622	a	0,01
Vi.B5	52316	12936	a	30096	10557	b	0,22
Vi.B6	22634	5171	b	31770	7231	a	0,30
X1454	76752	57055	b	5568616	845035	a	0,00
X521	3826769	3401904	b	555758619	107153220	a	0,00
X1458	811557	811557	b	71721967	14461398	a	0,00
X1451	1314136	1314136	b	112735814	22728868	a	0,00
X522	0	0	b	116315529	24655701	a	0,00
X497	0	0	b	90213690	19364204	a	0,00
X516	111940	71749	b	48073963	10480270	a	0,00
X286	96080	83609	b	9158831	2076667	a	0,00
X1260	1827530	794986	b	10519118	2210204	a	0,00
X1022	65746413	9897456	b	183112644	30980406	a	0,00
X41	160109871	12366966	b	245933061	18881757	a	0,00
X121	528573	50793	b	16006314	4804160	a	0,00
X1101	354050	100970	b	2036730	521265	a	0,00

X1077	192857	61329	b	1091312	278829	a	0,00
X46	17636496	1930597	b	29632248	2625445	a	0,00
X2466	1920	780	b	10684	2672	a	0,00
X1785	455	330	b	4971	1477	a	0,00
X1007	74920647	11368219	b	139271770	15238054	a	0,00
X42	11896074	1739259	b	21855032	2401101	a	0,00
X1028	206907	20490	b	462077	86502	a	0,00
X2320	27535	6223	b	94232	22481	a	0,00
X1069	43932	4045	b	81555	12322	a	0,00
X1095	298604	140644	b	1698508	476566	a	0,00
X1677	13032307	4201377	b	45844929	10722119	a	0,00
X1128	6738	2963	b	36086	10248	a	0,00
X92	118734049	1715351	a	110962984	1473843	b	0,00
X2558	85860	22185	b	298584	75386	a	0,00
X1411	152058	65379	b	687889	186846	a	0,00
X2149	4041	1506	b	16526	4444	a	0,00
X1026	228925	26270	b	477656	91867	a	0,00
X44	167259423	9999175	b	212223783	10033964	a	0,00
X1025	2145876	243728	b	7019296	1936655	a	0,00
X124	17968	2918	a	5885	2107	b	0,00
X254	65621512	1057682	a	61087464	914260	b	0,00
X157	271981	36837	b	565773	106565	a	0,00
X1385	8638780	172921	b	9373931	150001	a	0,00
X2272	5115	2125	b	20473	5512	a	0,00
X2476	1657	963	b	11536	3857	a	0,00
X138	3721632	539734	a	1505202	462355	b	0,01
X391	16336660	4245678	b	42675442	9243694	a	0,01
X1122	4441902	958057	b	9733697	1743249	a	0,01
X2388	6668	1932	b	28192	8780	a	0,01
X1053	29772440	432644	b	31527319	407648	a	0,01
X1851	601579	137855	b	1652558	422713	a	0,01
X211	1788	783	b	7720	2400	a	0,01
X2512	2309	1269	b	9749	2822	a	0,01
X1113	4442	1026	b	13770	4011	a	0,01
X158	160820	122951	b	935431	305409	a	0,01
X2070	177	127	b	959	306	a	0,01
X1856	103321	24595	b	286748	76830	a	0,01
X1777	367087	76856	b	759851	141675	a	0,01
X1316	1887	1113	b	22812	9652	a	0,01
X1181	32639	4519	b	66803	14462	a	0,01
X2600	778629	440043	b	10378011	4459472	a	0,01

X274	10118224	1360920	a	5434845	800973	b	0,01
X1604	31851	6806	b	74898	17521	a	0,01
X180	10468	4301	b	54372	19819	a	0,01
X1010	155375	59310	b	524481	151945	a	0,01
X1685	1924497	615502	b	6285617	1871787	a	0,01
X1750	76133	31375	b	263999	76817	a	0,01
X61	53309840	836844	b	56181535	569290	a	0,01
X161	33579	7202	b	72912	15726	a	0,01
X74	453638	48055	b	737729	118097	a	0,02
X1692	34378	14457	b	131492	42119	a	0,02
X1555	1163445	326666	a	145647	89519	b	0,02
X1049	336144	54546	b	1940215	791259	a	0,02
X34	14300600	1222345	a	9859786	1277356	b	0,02
X1006	8198914	1455699	b	14174206	2107729	a	0,02
X290	6432	5604	b	269978	134641	a	0,02
X528	1014	605	b	13853	6552	a	0,02
X1081	252411	99227	b	704253	178671	a	0,02
X5	80796	8048	a	51419	9124	b	0,02
X774	5122229	847931	a	2490175	466045	b	0,02
X792	3616216	605830	a	1747268	324593	b	0,02
X2754	2423	804	b	9225	3329	a	0,02
X1003	658422	129143	b	1399800	334565	a	0,02
X2503	103053	28057	b	256522	68300	a	0,02
X810	2733832	389838	a	1417510	358097	b	0,02
X1921	2031	1831	b	28239	13581	a	0,02
X62	89026386	8529020	a	56061631	11763499	b	0,02
X1004	16893	1923	a	10472	1809	b	0,03
X1833	2388	648	b	5763	1534	a	0,03
X2381	4860	1621	b	14606	4664	a	0,03
X1152	2004163	238872	b	2820442	255428	a	0,03
X126	61825822	9604980	a	34182426	3710260	b	0,03
X1747	1921	513	b	4389	1093	a	0,03
X357	714348	87694	b	1186276	221064	a	0,03
X1114	271460	45474	b	509700	110537	a	0,03
X433	28122	7053	b	68950	19604	a	0,03
X1567	6280	3225	b	18610	4671	a	0,03
X771	31643	6045	a	13852	3400	b	0,03
X2549	12338	5015	b	39705	13077	a	0,03
X1701	1339	359	a	310	181	b	0,03
X1960	337493	72706	b	947169	321196	a	0,03
X1014	2281227	508718	b	4318840	836524	a	0,03

X736	3115246	431240	a	1744372	406625	b	0,03
X1255	2326	623	a	563	322	b	0,03
X2099	25107	8419	b	72956	24101	a	0,04
X105	102552310	1141557	b	105901281	811311	a	0,04
X1560	1053	645	b	10705	5419	a	0,04
X125	3200	1249	b	11082	4096	a	0,04
X1097	1247994	287553	b	2579114	632230	a	0,04
X2361	6038445	1051141	a	2896060	876839	b	0,04
X1622	6980	2476	a	492	282	b	0,04
X101	287223701	2959402	b	295711617	2061514	a	0,04
X1150	1863182	216041	b	2547983	237907	a	0,04
X69	569858286	36207570	b	686318222	41842203	a	0,04
X1058	687208	160896	b	1319891	285054	a	0,04
X1008	397636	93892	a	156017	19304	b	0,04
X2007	2826	942	b	7524	2406	a	0,04
X1615	119435	28217	b	215353	38208	a	0,04
X21	1767166	242576	b	2601299	337841	a	0,04
X1631	17889567	1184102	b	21485799	1194443	a	0,05
X1184	74934	10661	a	44307	8729	b	0,05
X2080	111533	29290	b	227268	53384	a	0,05
X2619	184462	45065	a	65750	21626	b	0,05
X820	16240	6488	a	0	0	b	0,05
X55	11978598	4029466	a	1891388	551625	b	0,05
X1806	4397742	956666	a	1679762	794950	b	0,05
X2316	35130	10712	b	73476	16800	a	0,05
X543	9057	3331	a	749	537	b	0,05

Table S6. Deconvoluted total intensities (mean ± S.D.) of all identified in flowers organ in different antibiotic treatment within the plants receiving control levels of water in leaf. The statistically significant differences between treatments detected by Tukey's HSD post-hoc tests are indicated

by bold letters ($P< 0.05$). Metabolites as in Fig. 1 caption, the X represent the unknown metabolites.

Effect antibiotic in organ flowers							
**Metabolites**	**Antibiotic**			**Control**			
	**Mean**	**S.D.**		**Mean**	**S.D.**		**F(p<0)**
Ala	12554286	4222890	a	10357575	2464455	a	0,63
Arg	12442311	5657673	a	14003280	9171227	a	0,90
Asn	51367314	17884503	a	78332166	14903051	a	0,27
Asp	2354026	979582	a	5415417	1308290	a	0,11
Gln	12988760	5022682	a	10160294	2936602	a	0,61
Glu	1869132	913653	a	1849572	570973	a	0,98
Glup	21261418	9204542	a	18188280	5702406	a	0,77
His	20466757	10694040	a	32100782	6368054	a	0,33
HPro	400752	322618	a	256085	114559	a	0,62
Iso	90257759	31143126	a	135631658	33800129	a	0,37
Lys	213441	90324	a	110410	46910	a	0,27
Met	172133	69152	a	215954	158942	a	0,84
Phe	27844561	12616607	a	77141340	16561717	b	0,05
Pro	19203649	6875181	a	48000024	10672847	a	0,06
Ser	6284589	2107979	a	5020096	1431187	a	0,61
Thr	22677581	5314344	a	9984069	3186464	b	0,04
Try	15332547	7754862	a	19727936	7108512	a	0,69
Tyr	132652091	34048951	a	167475359	26577465	a	0,43
Val	119387911	37718338	a	113198210	22664995	a	0,88
Ad	49884206	17711762	a	51878497	14400200	a	0,93
AMP	5211	3519	a	9837	7680	a	0,66
Cy	59067	28851	a	129221	47351	a	0,29
Gua	640767	259849	a	614648	168321	a	0,93
Ur	52182	50042	a	204879	90556	a	0,23
Dis	496231	176829	a	971802	238483	a	0,17
Hex	20350122	7415038	a	26226136	6806038	a	0,58
Pen	766189	336339	a	2162744	357988	b	0,01
Raf	274	274	a	3156	1591	a	0,18
Xyl	2015564	659053	a	3291193	666175	a	0,21
Cit	60670879	16653699	a	27002082	8256190	a	0,05
Lac	15107475	3477865	a	20905342	5439485	a	0,45
Mal	77784355	27980783	a	165303447	34661330	a	0,09
OxA	30184	7591	a	18615	4966	a	0,19
PyA	1120620	506864	a	2810352	823064	a	0,15
ShA	605564	275767	a	662480	244158	a	0,88
AbA	799720	191883	a	1495191	321382	a	0,13

AsA	1799644	608992	a	2113973	623849	a	0,74
CafA	2113250	743593	a	2627864	916475	a	0,70
Cat	13599509	5413596	a	29077032	4775105	b	0,05
CGA	72268047	30482750	a	131619374	33409146	a	0,24
CGAp	34568052	15571016	a	64422387	14247761	a	0,19
Chr	144352	126418	a	6230	3029	a	0,16
CoA	1337284	584571	a	4439086	1282345	a	0,08
FeA	2621807	788717	a	10644383	5774172	a	0,30
Fis	3074967	1375537	a	3967602	934455	a	0,58
Hom	26429545	9971192	a	25710135	5006570	a	0,94
Hom.1	5752282	2266833	a	8516332	1809773	a	0,35
Kae	114192	48876	a	215737	50207	a	0,19
Pin	164710	51925	a	442595	121991	a	0,10
Pro.1	365758	159118	a	1223676	536089	a	0,24
Que	210314	74343	a	2842232	2041956	a	0,33
Rha	828446	338587	a	1220220	402257	a	0,51
Sal	249673	112448	a	156611	32734	a	0,34
Sap	32212001	9222193	a	67870937	11440843	b	0,04
SiA	2727	1518	a	3572	1209	a	0,67
SuA	49623074	22102666	a	50605700	15366517	a	0,97
Ani	459456	144665	a	3316836	2606077	a	0,41
Car	270137	183635	a	49798	22045	a	0,13
Cho	259708302	66884818	a	235099227	54489195	a	0,78
JaA	11429167	3066667	a	15254598	3530712	a	0,47
Log	2094916	532170	a	624523	173321	b	0,00
VaA	84727	29635	a	193288	47168	a	0,11
Nic	275404	98148	a	653569	168179	a	0,11
Rib	1613307	561972	a	911688	352316	a	0,27
Sec	4268047	2077032	a	6695398	2271181	a	0,48
Toc	6372681	1917428	a	3870228	1294775	a	0,27
Vi.B1	159109	60545	a	115988	36529	a	0,52
Vi.B5	469873	200437	a	2552486	1714648	a	0,36
Vi.B6	1357383	433315	a	4464891	802880	b	0,01
Vit	1394176	461190	a	672791	202538	a	0,11
X1007	44507888	9563324	a	19097257	4580510	b	0,01
X600	37422	14065	a	7504	3322	b	0,01
X423	13656932	2552174	a	5781340	1934195	b	0,02
X1451	13443177	7360862	a	7256	7256	b	0,02
X601	161682	83210	a	16986	5439	b	0,03
X2411	73811	41357	a	3203	1907	b	0,03
X480	6552037	1754818	a	2713788	824711	b	0,03

X2288	2984520	1509017	a	423233	157997	b	0,04
Thr	22677581	5314344	a	9984069	3186464	b	0,04
X2693	2251992	950251	a	495268	275431	b	0,04
X1260	2380367	1276983	a	264306	101394	b	0,04
X1871	1092984	600659	a	100941	56289	b	0,04
X285	316492	165965	a	42639	18161	b	0,04
X1175	2804559	1551489	a	307041	143427	b	0,04
X212	15767301	5414531	a	5384695	2235961	b	0,05
Cit	60670879	16653699	a	27002082	8256190	b	0,05
X422	178870	105952	a	15822	5203	b	0,05
X2169	749	419	a	91	66	b	0,05
X1284	1332097	711805	b	7249952	1206804	a	0,00
X1208	6041893	1893386	b	30714995	5398879	a	0,00
X2076	8626893	5442440	b	37538715	6309321	a	0,00
X2685	0	0	b	8953428	2296206	a	0,01
X2358	2599266	1021547	b	8353980	1362242	a	0,01
X2615	3338153	1156683	b	10896293	1855663	a	0,01
X2056	462959	132153	b	1787603	343946	a	0,01
X1526	30731682	11263399	b	123457723	23990685	a	0,01
Vi.B6	1357383	433315	b	4464891	802880	a	0,01
X1826	891400	388905	b	2989843	534090	a	0,01
X1300	1263619	562067	b	12175206	3127670	a	0,01
X2163	2047171	990083	b	13487988	3255952	a	0,01
X561	1528135	498223	b	6861195	1525252	a	0,01
Pen	766189	336339	b	2162744	357988	a	0,01
X1653	36106	11531	b	195774	46157	a	0,01
X2930	101176	38942	b	278383	47012	a	0,01
X1811	1085164	348565	b	3309728	624186	a	0,01
X1297	210	115	b	1674	428	a	0,01
X2378	147105	59555	b	385948	62277	a	0,02
X1527	118833806	46763672	b	389334677	76404186	a	0,02
X50	535872	200226	b	3837203	985030	a	0,02
X1282	17199433	7171476	b	55920407	11038634	a	0,02
X2021	2554737	1109838	b	11543951	2663015	a	0,02
X2244	3411224	2139173	b	14933911	3286380	a	0,02
X1922	723223	226153	b	2629096	573361	a	0,02
X2348	0	0	b	6818714	2105645	a	0,02
X2328	351141	272177	b	3986887	1117383	a	0,02
X2661	506442	361040	b	5886270	1665476	a	0,02
X1669	28556	22795	b	1261136	385034	a	0,02
X1862	71869	41475	b	767692	217340	a	0,02

X1624	659672	652405	b	6143224	1685602	a	0,02
X2082	37200177	16928813	b	96516815	15899493	a	0,02
X2867	200835	55103	b	480638	82516	a	0,02
X119	401634	266041	b	3247406	890488	a	0,02
X1788	1132101	543954	b	3667152	741335	a	0,02
X1093	1047867	413076	b	4182706	970034	a	0,02
X2125	23768	9822	b	4939	1970	a	0,02
X2280	194832	55399	b	1459015	405815	a	0,02
X2626	362705	181434	b	8267894	2545203	a	0,02
X2996	87947	31493	b	281618	60172	a	0,02
X2204	658142	229308	b	2219262	489616	a	0,02
X2414	1653435	931523	b	13136840	3699850	a	0,03
X1536	28774100	8832830	b	74733984	14159186	a	0,03
X2063	100844	41621	b	251826	43249	a	0,03
X1822	6806113	1688712	b	13972669	2141331	a	0,03
X1305	77066	27530	b	1204407	374664	a	0,03
X72	122985581	50743109	b	268838075	39050732	a	0,03
X1136	876674	277245	b	2506100	522977	a	0,03
X2466	91094	28115	b	449611	119472	a	0,03
X612	158026	60660	b	2473442	780740	a	0,03
X1671	2624331	961076	b	7597561	1588431	a	0,03
X2921	97759	66333	b	3870629	1278245	a	0,03
X2917	6830467	2531318	b	22157291	5020875	a	0,03
X2610	52336	18234	b	156531	33988	a	0,03
X1262	1428015	1140991	b	9062230	2530361	a	0,03
X736	1646061	437512	b	4077428	796674	a	0,03
X1945	448818	335939	b	2588250	714840	a	0,03
X1062	35821573	12938967	b	83210020	14762685	a	0,04
X1778	2978738	1343783	b	10160286	2395244	a	0,04
X274	1415984	454187	b	4361895	1000508	a	0,04
X2934	760900	578377	b	6929819	2143158	a	0,04
Sap	32212001	9222193	b	67870937	11440843	a	0,04
X807	22624254	8938246	b	53363589	9641457	a	0,04
X2005	1661923	1153445	b	10069905	2924024	a	0,04
X1131	519070	166209	b	3412819	1033903	a	0,04
X1934	4109473	1494296	b	13116220	3144055	a	0,04
X2751	195547	80537	b	678277	168798	a	0,04
X2107	196439	48246	b	514199	112175	a	0,04
X2329	3281950	1643433	b	14663635	4039703	a	0,04
X465	94575263	33449835	b	265762155	59556831	a	0,04
X1306	257262	139297	b	3312548	1117946	a	0,05

X2243	19063306	7754979	b	75146855	20100613	a	0,05
Phe	27844561	12616607	b	77141340	16561717	a	0,05
X1218	2043152	1376749	b	7972151	2027393	a	0,05
X2049	8254711	2712392	b	18626870	3473892	a	0,05
Cat	13599509	5413596	b	29077032	4775105	a	0,05
X1963	192574	84274	b	556879	125630	a	0,05
X2767	1812989	1314418	b	6770212	1696696	a	0,05
X1635	245102	106202	b	737004	174061	a	0,05
X505	920718	265874	b	3510607	960005	a	0,05

Table S7. Deconvoluted total intensities (mean ± S.D.) of all identified in flowers epiphytic in different antibiotic treatment within the plants receiving control levels of water in leaf. The statistically significant differences between treatments detected by Tukey's HSD post-hoc tests are indicated by bold letters (*P*< 0.05). Metabolites as in Fig. 1 caption, the X represent the unknown metabolites.

Effect antibiotic flowers in Epiphytic							
**Metabolites**	**Antibiotic**			**Control**			
	**Mean**	**D.S.**		**Mean**	**D.S.**		**Pr(>F)**
Ala	40233186	5212770	b	54587672	5753834	a	0,08
Arg	8646919	2988864	b	17726940	5020482	a	0,11
Asn	34382042	5301718	b	57129513	8206901	a	0,02
Gln	23540604	2431811	b	32340609	10404911	a	0,32
Glu	2026463	384332	b	2720925	591369	a	0,31
Glup	37501243	5270710	b	55998286	7914950	a	0,05
His	64876503	10615633	a	61132477	16776864	b	0,84
HPro	889976	635949	b	2744292	1414497	a	0,19
Iso	12579926	2630277	b	24585721	6923510	a	0,07
Lys	2232643	588441	b	7452538	2347532	a	0,01
Met	247559	81741	b	880692	377744	a	0,05
Phe	19111809	3255570	b	25634938	5394259	a	0,28
Pro	81425338	12101443	b	106541448	15053695	a	0,20
Ser	14983197	1740160	b	20364998	2562049	a	0,08
Thr	13459220	4578330	b	18626147	7173158	a	0,53
Try	2472901	602837	b	7187654	1481749	a	0,00
Tyr	73468281	5616017	b	86747526	7212408	a	0,15
Val	60340515	9496890	b	86295734	12816484	a	0,11
Ad	2112981	707220	b	7771432	1632213	a	0,00
Ade	850246	394619	b	1052207	880057	a	0,81
AMP	498386	288902	a	212917	100127	b	0,45
Cy	67426	19011	b	137680	41877	a	0,09
Gua	148103	38436	b	267618	85240	a	0,16
Ur	18403	8110	a	17314	9867	b	0,93
Dis	3068096	569718	a	3036375	808466	b	0,97
Hex	7164943	620560	b	9914967	1754756	a	0,09
Pen	1040180	146953	b	1550692	400764	a	0,17
Raf	844757	603956	b	9286481	9230506	a	0,26
Xyl	2031923	345432	b	4724610	939655	a	0,00
AbA	813475	268843	b	1335340	405028	a	0,27
AsA	531416	156499	b	880028	342996	a	0,31
CafA	156218	69111	b	431591	154100	a	0,07
Cit	27541016	3215042	b	29652428	6197671	a	0,74

Lac	7895167	1472966	a	6796028	1278008	b	0,61
Mal	151518621	19532854	a	136784226	23259803	b	0,63
OxA	58484	16536	a	39939	7823	b	0,40
PyA	262581	55893	b	328006	87239	a	0,51
ShA	396121	80963	a	293314	126289	b	0,48
SuA	24993846	3206066	b	30784798	6587252	a	0,39
Cat	5437423	1106683	b	8629681	1517278	a	0,09
CGA	39523304	7980079	a	32720474	8507939	b	0,58
CGAp	6736699	1574693	b	10844446	2478175	a	0,15
Chr	156081	81711	b	305968	181995	a	0,40
CoA	407189	97885	b	1017748	319070	a	0,04
FeA	2386794	634971	a	1632326	424775	b	0,39
Fis	1167525	405953	b	2834424	1091470	a	0,11
Hom	11834369	3025075	b	24102318	7630151	a	0,09
Hom.1	8698794	1598818	b	11112751	3414509	a	0,48
Kae	739637	360902	b	1000388	340438	a	0,62
Pin	129449	33666	b	153211	58934	a	0,71
Pro.1	1099857	517628	b	1128365	494683	a	0,97
Que	206330	131171	a	53165	33065	b	0,37
Rha	2314876	1179908	a	889844	579645	b	0,37
Sal	372728	109012	a	226997	38859	b	0,31
Sap	13747811	2803210	b	15018805	4642431	a	0,80
SiA	7122	1402	a	4085	1193	b	0,14
VaA	60290	10573	b	65503	16720	a	0,78
Ani	1849169	1085971	b	8890685	4516366	a	0,07
Car	550110	522630	a	157630	124216	b	0,56
Cho	134295492	11798414	b	165267063	16177523	a	0,12
JaA	28672	11123	a	21199	7159	b	0,62
Log	49195	13062	b	91234	29331	a	0,15
Nic	150270	55673	a	114965	66831	b	0,69
Rib	174263	57018	b	426959	141234	a	0,07
Sec	2289826	555431	b	4119689	1954506	a	0,29
Toc	9847319	1034095	b	10998516	1255513	a	0,49
Vi.B1	31602	8208	b	54166	16775	a	0,19
Vi.B5	440870	131743	a	418334	105125	b	0,90
Vi.B6	350257	84120	b	400004	121818	a	0,73
Vit	1945181	500845	b	3276606	981113	a	0,19
X286	0	0	b	300199	84843	a	0,00
X1015	919513	153703	b	2421734	437571	a	0,00
X1765	1985	938	b	7592	1333	a	0,00
X542	46962	8093	b	121798	24519	a	0,00

X259	12223	4807	b	53365	13612	a	0,00
X250	741636	116561	b	1876381	392358	a	0,00
X1143	2467862	1065000	b	12083837	3416165	a	0,00
X2719	25732	16266	b	222875	75942	a	0,00
X516	6173	3563	b	4402456	1836839	a	0,00
X378	16210	4402	b	89576	30700	a	0,01
X1295	664979	179669	b	2296688	643517	a	0,01
X254	69249502	1170455	a	63721654	1501521	b	0,01
X1501	714810	231957	b	2547050	702401	a	0,01
X2539	1737071	563319	b	7322183	2269350	a	0,01
X92	123829965	1880917	a	115297995	2410731	b	0,01
X2716	112549	56628	b	618599	212078	a	0,01
X1106	30935	7242	b	163194	59609	a	0,01
X2435	177881	120911	b	1823253	734886	a	0,01
X109	227990	121271	b	1094554	352193	a	0,01
X78	16514512	2087620	b	28498170	4460062	a	0,01
X1007	75839880	14466393	b	166835192	35852202	a	0,01
X269	5828349	1574417	b	14939814	3550638	a	0,01
X227	8944	2062	b	28976	9244	a	0,01
X217	1027257	161500	b	1781225	271755	a	0,02
X1454	775	540	b	459937	233557	a	0,02
X42	26969237	1797442	b	34851117	2819520	a	0,02
X1405	361593	132713	b	2615570	1131521	a	0,02
X1026	201043	29110	b	335018	51194	a	0,02
X49	1978715	476224	b	4312352	934589	a	0,02
X87	1737953	326603	b	3408190	690130	a	0,02
X1338	3082625	537393	a	1227232	436592	b	0,02
X41	258645785	15466193	b	326324782	25103614	a	0,02
X781	5179	2011	b	22134	8352	a	0,02
X2330	17850	8195	b	102956	43235	a	0,02
X543	18405	5285	b	53591	16713	a	0,02
X1489	447470	164711	b	1492880	494427	a	0,02
X79	60638	14474	b	3043598	1609057	a	0,03
X46	33364247	2103558	b	42190470	3399114	a	0,03
X23	6608541	1062104	b	10843163	1566465	a	0,03
X2813	41891	18823	b	177929	68330	a	0,03
X1498	7977371	1923472	b	16896155	3824319	a	0,03
X2227	37303	23334	b	145327	46489	a	0,03
X2322	92813	28210	b	293145	101855	a	0,03
X2221	198038	89491	b	634917	197761	a	0,03
X2178	3727904	1588037	b	18270496	7760381	a	0,03

X606	21267	6586	b	56140	16562	a	0,03
X18	3041587	1155369	b	9370526	3064605	a	0,03
X90	29625625	4874813	b	51026123	9325767	a	0,03
X834	6553	2424	b	50285	24750	a	0,03
X538	555107	150058	b	1297846	354064	a	0,03
X350	1132616	325946	b	4229483	1714557	a	0,04
X1576	185788	53953	a	35266	14205	b	0,04
X2149	8576	2319	b	28763	11359	a	0,04
X1329	78799829	23661325	a	14132121	7715724	b	0,04
X13	63096855	11622290	b	127199558	33452447	a	0,04
X158	147175	111117	b	671020	257996	a	0,04
X3	1516944	310211	b	2592658	417325	a	0,04
X1040	144218	42661	b	405995	142248	a	0,04
X342	9805	3152	b	36573	15327	a	0,04
X368	36239	10120	b	95960	32370	a	0,04
X121	633804	98599	b	1196432	302580	a	0,04
X1146	43077	19620	b	128840	41518	a	0,04
X240	22079	7660	b	99064	45028	a	0,04
X766	25362	9315	b	105245	46162	a	0,04
X552	119011	26699	b	227085	49951	a	0,04
X10	20389587	1431612	b	25441447	2104068	a	0,05
X1171	654152	168603	b	1354756	338857	a	0,05
X1068	119849	30764	a	39882	7117	b	0,05

Table S8 Abbreviation, family and the real name of the metabolites detected.

Abreviation	Family	Metabolite
Glu	AA	Glutamic acid
Asp	AA	Aspartic acid
Ala	AA	Alanine
Arg	AA	Arginine
Asn	AA	Asparagine
Gln	AA	Glutamine
His	AA	Histidine
HPro	AA	Hydroxy-Proline
Iso	AA	Isoleucine
Lys	AA	Lysine
Met	AA	Methionine
Phe	AA	Phenilalanine
Pro	AA	Proline
Ser	AA	Serine
Thr	AA	Threonine
Try	AA	Tryptofan
Tyr	AA	Tyrosine
Glup	AA	Glutamic acid
Val	AA	Valine
Ad	BN	Adenine
Ur	BN	Uracil
Ade	BN	Adenosine
Cy	BN	Cytidine
Gua	BN	Guanosine
Ur	NS	Uridine
AMP	NT	AMP
Cit	OA	Citric acid
Lac	OA	Lactic acid
Mal	OA	Malic acid
OxA	OA	Oxaloacetic acid
PyA	OA	Pyruvic acid
ShA	OA	Shikimic acid
SuA	OA	Succinic acid
AbA	OA	Abscisic acid (ABA)
AsA	OA	Ascorbic acid (Vit C)
Cat	OA	Catechin
CGAp	OA	Chlorogenic acid
Ani	OT	Adonitol (Ribitol)
Toc	OT	d-tocopherol
JaA	OT	Jasmonic acid

Vi.B6	OT	Pyridoxine (Vit B6)
Rib	OT	Riboflavin (Vit B2 - Vit G)
Vit	OT	Vitexin
Car	OT	Carvone
Sec	OT	Secologanin
Log	OT	Loganin
Cho	OT	Choline
Nic	OT	Nicotine
Vi.B5	OT	Pantothenic acid (Vit B5)
Vit.B6p	OT	Pyridoxine (Vit B6)
Vi.B1	OT	Thiamine (Vit B1)
CafA	PH	Caffeic acid
CGA	PH	Chlorogenic acid
Chr	PH	Chrysin
CoA	PH	Coumaric acid
Pin	PH	D-Pinitol
FeA	PH	Ferulic acid
Hom	PH	Homoorientin
Kae	PH	Kaempferol
Pro	PH	Protocatechuic acid
Que	PH	Quercetin
Rha	PH	Rhamnetin
Sap	PH	Saponarin
SiA	PH	Sinapinic acid
Sal	PH	Sodium salicylate
VaA	PH	Vanillic acid
Fis	PH	Fisetin
Hom	PH	Homoorientin
Rhap	PH	Rhamnetin
Dis	S	Disaccharides
Hex	S	Hexoses
Pen	S	Pentoses
Raf	S	Raffinosepentahydrate - Maltotriose
Xyl	S	Xylitol - Arabitol
